# “A combination of everything”: a mixed-methods approach to the factors which autistic people consider important in suicidality

**DOI:** 10.1177/25739581251371393

**Published:** 2025-09-04

**Authors:** R.L. Moseley, S.J. Marsden, C.L. Allison, T.A. Parsons, S. Cassidy, T. Procyshyn, M. Pelton, E.M. Weir, T. Chikaura, D. Mosse, I. Hall, L. Owens, J. Cheyette, D. Crichton, J. Rodgers, H. Hodges, S. Baron-Cohen

**Affiliations:** 1 Department of Psychology, Bournemouth University, Dorset, UK; 2 Department of Haematology, University of Cambridge, UK; 3 Autism Research Centre, Department of Psychiatry, University of Cambridge, Cambridge, UK; 4 Autism Centre of Excellence at Cambridge (now Autism Action), Cambridge, UK; 5 School of Psychology, University of Nottingham, Nottingham, UK; 6 Department of Anthropology and Sociology, SOAS University of London, London, UK; 7 East London NHS Foundation Trust, London, UK; 8 Ex GP research lead for East Dorset, UK; 9 Population Health Sciences Institute, Faculty of Medical Sciences Newcastle University, Newcastle, UK

**Keywords:** Suicidal thoughts, suicide attempts, qualitative, risk factors, gender, age, autism

## Abstract

**Background:**

Suicide is a leading cause of death for autistic people, but inadequately explained by theories derived in non-autistic populations. Autistic people’s perceptions of the factors underpinning suicidal experiences are vital for guiding conceptual understanding, risk assessment, and policy and clinical practice towards preventing suicide.

**Methods:**

We recruited 1369 autistic participants for an online survey designed through consultation with autistic people. Participants were 326 cisgender men, 718 cisgender women, and 325 transgender or gender-divergent individuals, ranging from 16-89 years old. We asked them to rate the importance of 19 contributing factors to their suicidal thoughts and feelings, and enter their own explanations of additional factors if desired. Alongside thematically analysing this qualitative data, we examined whether ratings of contributing factors differed by age and gender, and whether ratings statistically predicted levels of lifetime suicidality.

**Results:**

Loneliness, feelings of worthlessness/failure, hopelessness and mental illness were the highest rated contributing factors to suicidal thoughts and feelings, particularly by autistic women and sex/gender minorities; ratings also differed by age. Qualitative responses indicated the complexity of suicidality, wherein autistic status influenced both the nature of the stressors (e.g. societal stigma) and cognitive-emotional states (e.g. feeling disconnected through feeling different to others) that participants identified. Greater perceived importance of bullying, difficulties accessing support and past trauma characterised participants with experience of suicide plans or attempts.

**Conclusion:**

While some of the experiences and mental states identified by participants resembled those identified in non-autistic groups, the psychological profile of autistic participants and their experiences of marginalisation appeared to heavily contextualise expressions of hopelessness, burdensomeness, worthlessness, loneliness and entrapment. Autistic people vary with regards the factors perceived to underpin suicidality. However, associations between suicidality and the perceived importance of bullying, trauma, and inability to access support highlight the necessity of societal and systemic change to prevent suicide.

## Community brief

### Why is this an important issue?

Autistic people are more likely to die by suicide than are non-autistic people. To change this, we need to understand why suicidal thoughts and feelings are relatively common in autistic people, and why some end their lives.

### What was the purpose of this study?

As part of a larger project about suicide prevention, we wanted to hear from autistic people themselves about the factors which contributed to their suicidal thoughts and feelings. Secondly, we wanted to see if these contributing factors differed across autistic people of different ages, genders, and age groups. Thirdly, we wanted to see if these contributing factors were different in autistic people who had attempted suicide.

### What did the researchers do?

We designed an online survey, drawing on feedback from a large group of autistic people. The survey included 19 factors that might contribute to suicidal thoughts and feelings, which were based on existing research and suggestions from autistic people in the design phase. We asked survey participants to rate the importance of these factors and describe additional factors in their own words. Altogether, 1369 autistic people took part.

### What were the results and conclusions of the study?

We found that loneliness, hopelessness, feelings of worthlessness or failure, and mental illness were rated as the most important factors contributing to suicidal thoughts and feelings. In their own words, participants said that suicidal thoughts and feelings were caused by the stress of major life events; by the strain of everyday living without support; by instances where they were victimised by others, including professionals; and by societal stigma and constant pressure to mask. Autistic people of different genders and ages differed in the factors which contributed to their suicidal thoughts and feelings. For example, cisgender women and gender-divergent participants rated being unable to access support, difficulties with family/friends, past trauma and mental illness as more important than did cisgender men. Autistic people who had attempted suicide tended to highlight the importance of past trauma and inability to access support.

### What is new or controversial about these findings?

We believe ours is the first study to directly ask autistic people about the factors which contributed to their suicidal experiences. Having a large sample meant we could look for differences related to age and gender. Our findings place responsibility for change on society and systems, rather than viewing suicidal thoughts as a kind of sickness or abnormality in autistic people.

### What are potential weaknesses in the study?

We cannot confirm that the contributing factors identified by autistic people really did cause their suicidal experiences. Our findings might not be relevant to autistic people with learning disabilities, people of colour or people from other countries.

### How will these findings help autistic adults now or in the future?

The findings highlight that society needs to change in order to reduce suicide in autistic people. Specifically, governments must tackle the stigma and abuse that can traumatise autistic people, and ensure they have sufficient and appropriate support.

**Figure 1:**
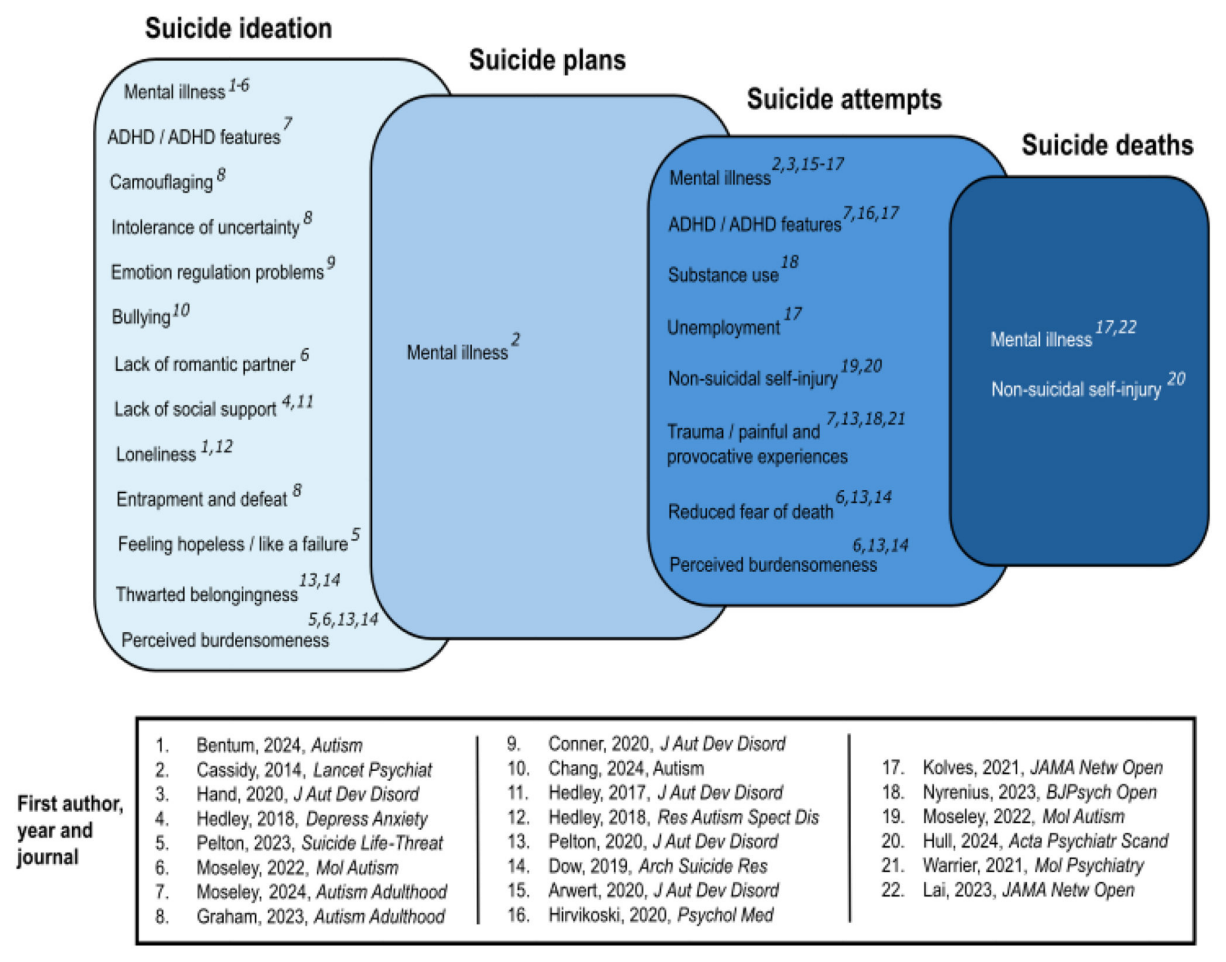
Factors linked to suicide ideation, plans, attempts and deaths in autistic people *Note*. Reported correlates of suicide ideation, plans, attempts and deaths in autistic people. We have not depicted correlates linked to ‘suicidality’ broadly without distinctions between thoughts and behaviour, or correlates whose effects, in the literature, are contradictory: greater cognitive flexibility has, for instance, been linked both with lower risk of suicide ideation,^1^ and with greater suicide ideation and plans^2,3^. Similarly, though some find elevated attempts^4,5^ and greater relative risk of suicide death associated with female sex assigned at birth,^6^ but overall more autistic males than females die by suicide.^6–8^

Suicide is a leading cause of death for autistic people without learning disabilities (LD^a^).^1,2^ Across countries of varying incomes, lifetime prevalence of suicide ideation and attempts is approximately 1 in 10 and 1 in 37 of the general population^3^; in autistic people without LD their lifetime prevalence is estimated at between 1 in 3 and 1 in 5.^4^ While researchers have identified correlates of suicide thoughts and behaviour (STB^b^) in autistic people (Figure 1), few factors differentiate those who act on suicidal thoughts. This distinction is vital for preventing early, avoidable deaths, since different factors underpin the transition from ideation to action.^5–7^

Psychological theory has guided efforts to understand, predict and prevent suicide in non-autistic populations.^8^ The interpersonal theory of suicide (ITS),^6^ for example, has inspired interventions addressing the factors hypothesised to drive suicide ideation (‘thwarted belongingness’, meaning loneliness and lack of belonging, and ‘perceived burdensomeness’, meaning beliefs that one is a burden to others).^9^ Critically, it first postulated that different factors, such as reduced fear of death, enable transition from suicidal thoughts to attempts.^6^ Suicide prevention in autistic people, however, is hampered by the fact that well-established theories do not necessarily apply or operate equivalently.^10–14^ There is some evidence that reduced fear of death does, as originally suggested, distinguish autistic people who have attempted suicide.^10,12^ However, thwarted belongingness only sometimes predicts suicide ideation in autistic people,^10,12,15^ and may operate indirectly through burdensomeness and low mood.^11^ Moreover, where perceived burdensomeness was originally linked to suicide ideation,^6^ in autistic people it also seems important for suicide attempts.^10–12^ Beyond theory-derived constructs, other intrapersonal and autism-related factors, such as temperament and cognitive flexibility^1,16–19^ likewise fail to consistently predict different degrees of STB in autistic people.

Autistic people’s own perceptions of the causal factors for STB are a vital source of information which is surprisingly absent from present literature. The majority of studies on suicide in autistic people have examined social-psychological constructs defined in non-autistic people.^10–15,19–25^. This can however be problematic, as shown in the case of the Interpersonal Needs Questionnaire (assessing thwarted belongingness and perceived burdensomeness),^26^ because measures may not capture constructs as originally conceptualised in non-autistic populations. Autistic people allude indirectly to STB in the context of life changes and adversity^27–30^, trauma,^31^ barriers to support,^32^ meltdowns,^33^ masking,^34^ burnout,^35^ and living undiagnosed^36^ – but we lack qualitative approaches that demonstrate how they think about STB and the causal factors underpinning it. Directly exploring the factors that autistic people endorse as important to STB may aid risk assessment and guide intervention, while facilitating conceptual understanding of whether contributing factors differ from those defined in neurotypical populations.

Herein, in a three-part analysis involving autistic participants with varying degrees of STB, we explored factors that these individuals themselves report as important to STB. In our first quantitative and qualitative analyses, we examined how participants rated the importance of pre-specified factors that might contribute to STB, and which factors they themselves identified as important.

In the second, quantitative part of our analyses, we examined whether, much like research and support priorities,^37,38^ perceived contributing factors to STB differ statistically by sex, gender and age. Based on extent literature, we predicted that mental and physical illness, trauma, victimisation and unmet needs might be particularly important factors for cisgender women, transgender and gender-divergent participants^39–43^; that academic strains and gender and/or sexual identity difficulties might be more important for younger participants,^44^ and employment, housing, legal and financial issues, physical health problems, and bereavement more important for older participants. We expected victimisation and loneliness to be equally important across age and gender groups, given their ubiquity.^10,45–47^

In the third quantitative part of our analyses, we returned to the distinction between suicidal thoughts and behaviour by examining whether differences in the perceived importance of contributing factors could statistically predict varying levels of lifetime STB, and particularly distinguish autistic people with experience of suicide attempts. In a data-driven approach without prior hypotheses, we focused on the pre-specified contributing factors that had been identified as most important in the first part of these analyses.

## Methods

### Participants

We advertised our online survey as a call for ideas for suicide prevention (reported elsewhere^48^), promoting it on Facebook, YouTube, Twitter/X, Instagram, and the website of the charity the Autism Centre of Excellence at Cambridge (ACE: now Autism Action). We set eligibility criteria that required autistic participants to be aged 16 or over. Following the data cleaning process (see Supplementary Materials 1), we retained datasets from 1,369 participants, all of whom had experience of STB^c^, and 1216 of whom completed the whole survey. Almost 94% resided in the UK (see Table 1 and Supplementary Materials 1).

To better understand our participant group, we conducted several initial comparisons. These showed that cisgender women and transgender, gender-divergent or gender-questioning participants were more likely to have greater lifetime STB; so too were formally diagnosed autistic participants, and those with each kind of co-occurring condition. Differences in the distribution of employment and education categories had less clearly discernible effects.

### Materials and procedure

We incorporated suggestions from autistic people and their families in development of our Qualtrics survey, and followed guidance for safely conducting research on sensitive topics.^12^ The Cambridge University Psychology Research Ethics Committee approved this survey (PRE.2022.097), and the University of Cambridge sponsored it. Most participants completed the survey within 25 minutes (median completion time was 21.6); unfortunately, we could not reimburse their time.

Through the survey, we began by asking participants demographic questions (e.g. age and gender) and “what [their] mood is like right now” (from 0=“My mood is very bad”, to 10=“My mood is very good”). We then asked about lifetime experience of STB (our principal dependent variable). Where a small minority of participants reported never experiencing STB, we forwarded these individuals to a later part in the survey which focused on ideas for suicide prevention (reported elsewhere^48,49^). Where participants indicated experience with STB, we asked several follow-up questions to assess the extent of that experience (e.g. number of suicide attempts if relevant), as well as the factors which contributed to suicidal thoughts and feelings (our principal independent variable). Subsequently, we asked participants about their experiences of help-seeking (reported elsewhere^50^), psychological interventions for STB, app use, and their ideas for suicide prevention. Finally, we asked participants to rate their mood again (with an average difference of -.03 between the start and end of the study, present mood did not deteriorate significantly for most participants); subsequently, we debriefed participants and provided them with support resources.^12^

We derived our key variable of interest, contributing factors to STB, from the question, “Which were the most important factors that contributed to your suicidal thoughts and feelings?”. On a scale from “Not at all important/relevant” (0), “Slightly important” (1), “Moderately important” (2), to “Very important” (3), we asked participants to rate 19 pre-specified items based on prior research and suggestions from autistic people during the design stage (see Supplementary Materials, 2). The list included concrete life events or experiences and mental states, some akin to constructs from theories of suicide. Optionally, we invited participants to enter additional factors via free-text.

We predicted that contributing factors would differ as a function of gender (categorised as per Table 1) and age. In the second part of our analysis, we operationalised age as a categorical variable within a factorial design (with levels as per Table 1); in the third, regression-based part of the analysis, we treated it as continuous. To examine the effects of these independent variables, we controlled for several possibly confounding variables including ethnicity (coded as white [0] or ethnic minority [1]); autism diagnostic status (formally diagnosed [1] or possibly autistic [0]); highest educational attainment (no formal qualifications above GCSE level or equivalent [1], AS/A-Level or equivalent [2], diploma, certificate of higher education or degree level [3], postgraduate level [4], or undisclosed [0]); and employment status (employed or a student [1]; caregiving or doing voluntary work [2]; unemployed [3]; retired or undisclosed [4]).

For our principal dependent variable of interest, we used a measure of lifetime STB. We assessed this as per one question from the Suicide Behaviours Questionnaire-Autism Spectrum Conditions (SBQ-ASC^51^): “Have you ever thought about or attempted to end your life?” We coded response options accordingly: “Never” (excluded from analyses); “I have thought about it briefly, e.g. a passing thought” (1); “I have seriously thought about ending my life, but did not plan how or try to do it” (2); “I have planned to end my life, but did not try to do it” (3); “I have made at least one suicide attempt which I planned in advance” (4); “I have made at least one suicide attempt which was not planned in advance” (also coded 4). The SBQ-ASC was created with and for autistic people.^51^ During the validation process, the authors checked that autistic people interpreted these response options as the authors intended (in accordance with the definitions previously provided).

### Analysis

We implemented a three-part analysis addressing the following research questions:

#### Part 1: What do autistic people self-report as the most important factors in suicidal thoughts and feelings?

Firstly, we plotted average importance of the 19 pre-specified contributing factors. We also analysed qualitative data from participants who provided free-text responses to the question about contributing factors for suicidal thoughts and feelings (n=510: 111 cisgender men, 278 cisgender women, and 121 transgender, gender-divergent or gender-questioning participants). We used logistic regression to briefly understand who, among our participants, was most likely to provide qualitative data (see Supplementary Materials, 3): these analyses indicated that older participants and people with higher levels of lifetime suicidality were more likely to have provided qualitative data. Autistic men were less likely than other gender groups to have provided qualitative data.

RLM and SJM analysed the qualitative data in an inductive thematic approach,^52^ consistently reflexive to their positionality as autistic with experience of STB. Having familiarised themselves with the data, both coded the first 100 quotations and generated agreed codes which were iteratively refined through coding the remaining data. The authors discussed, refined and agreed their interpretations of higher-order themes through development of the thematic table. The whole research team reviewed the analysis and written narrative.

#### Part 2: Do ratings of importance for contributing factors differ by sex and gender, or age?

Secondly, we modelled the 19 contributing factors as a within-subject variable in a mixed ANOVA (n=1323). For this analysis only, listwise deletion meant that we needed to remove 46 participants who did not respond to every pre-specified contributing factor. Some ignored all factors except those deemed “very important/relevant”; although this could indicate that the other, ignored factors were unimportant, we were reluctant to presume this, and so removed these participants from this analysis. We modelled age (treated categorically within this factorial analysis, with levels as per Table 1) and gender identity (3 levels as per Table 1) as between-subject factors. We controlled for ethnicity, autistic status, educational attainment and current employment.

We interpret main effects and interactions with respect to a standard alpha level of p <.05. Because of sphericity violations for our within-subject variable, we report Greenhouse-Geisser values; otherwise, we found that the data satisfied assumptions of normality and homogeneity of variance. We investigated significant two-way interactions^6^ between contributing factors and age group or gender with planned univariate tests (using the youngest group and cisgender men as reference categories in simple contrasts). For these, we used all participants who had rated that contributing factor, and corrected alpha levels to a false discovery rate of p <.05.

#### Part 3: Do ratings of the most important factors in suicidal thoughts and feelings differentiate between individuals with different degrees of STB?

We adopted a broad approach to contributing factors to STB in the first two parts of our analysis. In the third part, we narrowed our focus to only those pre-specified factors which were rated as most important in the first stage, and examined whether these could predict different degrees of STB in all 1369 participants. For the 9 (of 19) contributing factors with highest average importance (all over 1.5), we created binary variables indicating whether (1) or not (0) participants had rated that factor as ‘very important’ (a threshold we chose for specificity, because these items were frequently endorsed with at least some degree of importance). These factors were: ‘Academic difficulties/stress’, ‘Bullying, abuse, harassment or assault’ (henceforth ‘Bullying’), ‘Difficulties in relationships with family/friends’, ‘Loneliness or feeling disconnected/alienated from others’ (henceforth ‘Loneliness’), ‘Feeling worthless or like a failure’ (henceforth ‘Worthlessness’), ‘Hopelessness’, ‘Being unable to access support you needed’, ‘Mental health problems’, and ‘Trauma from past events’ (henceforth ‘Past trauma’). We confirmed these 9 binary variables were independent (with the Lambda statistic, indicating the absence of multicollinearity) and then included them as predictors in three regression analyses (alpha levels corrected to p =.017).

In the first multinomial regression we included just these 9 binary variables as predictors of group membership in relation to lifetime STB (a dependent variable with four levels: passing thoughts, suicide ideation, suicide plans, and suicide attempts [reference category]). Second, we repeated this analysis including gender, age (treated continuously), and our four covariates. We performed a third, binary logistic regression to corroborate and more clearly delineate participants with and without lifetime suicide attempts. Here, we entered contributing factors in the first block of the model, gender and age in the second block, and covariates in the third. Before each analysis, we ensured the data met the assumptions of multinomial or logistic regression with regards the absence of outliers and multicollinearity in continuous variables, and linearity between these and the logit transformation of the dependent variable (lifetime STB).

## Results

### Part 1: Factors contributing to suicidal thoughts and feelings

**Figure 2:**
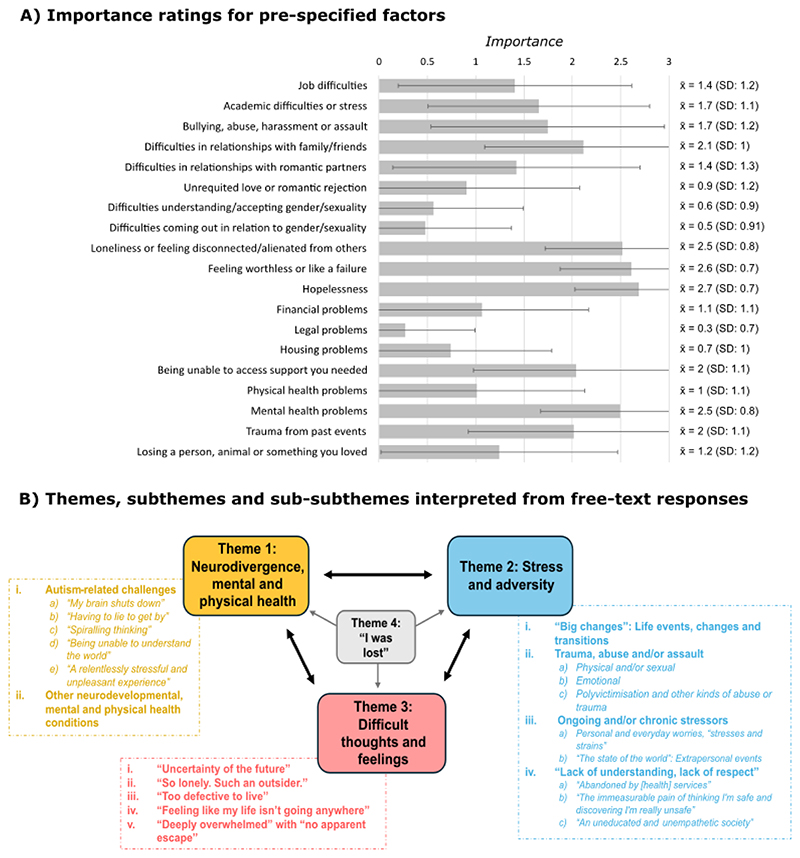
Factors which contributed to suicidal thoughts and feelings: quantitative and qualitative data *Note*. Part A shows average ratings of the importance of 19 factors pre-specified in the survey (listed in order of presentation), where 0 indicated not important/relevant at all, and 1, 2 and 3 indicated slightly, moderately and very important respectively. We also present average importance ratings for each item to the right of the graph, with standard deviations in brackets. Part B depicts the four themes (coloured boxes) interpreted in free-text responses, along with subthemes (bold text, roman numerals) and sub-subthemes (italic text, alphabetical numerals) which were interpreted for Themes 1-3.

Of pre-specified factors, participants rated Mental health problems, Loneliness, Worthlessness, and Hopelessness as most important (Figure 2, Part A). These and other factors were reflected and enriched in the qualitative data, which demonstrated the multifactorial and complex nature of STB. We interpreted four interrelated themes, some with subthemes and sub-subthemes, in the data (Figure 2, Part B; thematic table in Supplementary Materials 4).

#### Theme 1: Neurodivergence, mental and physical health

Participants frequently alluded to neurodivergence and to mental and physical health conditions in relation to suicidal experiences, as per two subthemes.

##### Autism-related challenges

Frequent references to specific aspects of being autistic were reflected in five sub-subthemes. In the first, ^7^*“My brain shuts down”*, participants described instances of sensory and emotional challenges, meltdowns, shutdowns and burnout. Burnout severely affected daily functioning, and some had insufficient support to recover. Sensory pain/discomfort was described in terms of “assault”, “affliction” and “overload”, and linked to navigating complex, inaccessible environments. Participants described “big”, “intense” and unmanageable emotions, and sensitivity to other people’s emotions. For some participants, emotion non-acceptance was critical:

“Even if times are hard, it is not life events, but being angry at myself for not being able to control or manage my low mood that causes suicidal feelings”.

In the second sub-subtheme, *“Having to lie to get by”*, participants pointed out the “emotional exhaustion” and identity-confusion associated with masking. “Playing a character” sometimes resulted in others’ underestimating their needs and/or disbelieving their suicidal feelings. Negative social evaluation and resultant pressure to mask led, for one participant, to a “spiral of despair”.

In the third sub-subtheme, *“Spiralling thinking”*, participants described thinking patterns which could augment the impact of adversity. These included “worst-case scenario reasoning”, dichotomous thinking in the context of moral unambiguity (“breached my own ethical code”), and “obsessive and intrusive” thoughts, whether focused on self-evaluation or even a parental suicide. Some participants explained that suicidal thoughts could be “a background noise… a rhythm to rumination and loop thinking”, not necessarily related to current events. One participant mentioned suicidal thoughts as a hyper-fixation, while others linked them to being unable to access their interests.

The fourth sub-subtheme, *“Being unable to understand the world”*, constituted incidents where participants cited their own difficulties communicating, initiating and sustaining contact, and understanding “what is happening in important situations” or “the world in general”. They spoke of resulting estrangement from family, inability to verbalise abuse, and having services “taken away… for ‘not engaging’”.

The fifth sub-subtheme, *“A relentlessly stressful and unpleasant experience”*, described instances where participants linked suicidal thoughts and feelings to being autistic in a general sense.

##### Other neurodevelopmental, mental and physical health conditions

Participants mentioned ADHD and a range of mental (e.g. depression, PTSD, OCD, psychosis) and physical health conditions (e.g. chronic pain and fatigue) which threatened sense of self and made life “more and more difficult to endure”. Other health-related challenges included sleep disruption, gender dysphoria, self-harm, medication side-effects and withdrawal effects, and body image distress and disturbance. Some participants linked substance addiction to feelings of loneliness, hopelessness, and suicide attempts.

#### Theme 2: Stress and adversity

This theme described different stressors related to suicidal thoughts and feelings. While some were rateable pre-specified factors in the survey, participants often expanded on the complexity and specificity of events, which were often interrelated with aspects of neurodivergence and/or health (Theme 1), mental states (Theme 3), or being undiagnosed (Theme 4). We interpreted four subthemes as follows:

##### “Big changes”: life events, changes and transitions

This subtheme describes major life events or transitions, some biological (childbirth, menopause), others social (e.g., leaving home, relationship breakdowns, bereavement). Many described complex, multiple life changes co-occurring.

##### Trauma, abuse and/or assault

This subtheme encapsulated three sub-subthemes reflecting different forms of trauma, abuse and/or assault. The first described instances *that were physical and/or (more commonly) sexual*. The second described *emotional abuse*, from “constant criticism” to emotional neglect. The third described instances of *poly-victimisation, other kinds of abuse and trauma*, such as traumatic deaths of loved ones through murder or suicide. One participant expressed that the pre-specified item about ‘relationship difficulties’ was not representative of the “trauma” and “sudden collapse of mental and physical health” following a partner’s infidelity, indicating that autistic people may experience trauma from a wider range of factors than non-autistic people.

##### Ongoing and/or chronic stressors

This subtheme reflected impacts of different kinds of stressors than those listed in the survey. Within it, we interpreted two sub-subthemes:

In *Personal and everyday worries, “stresses and strains”*, autistic people described feelings of “stress”, “exhaustion” and “overwhelm” associated with everyday stressors which impacted them particularly severely, such as being a carer or parent; managing multiple roles; conflict with family; financial insecurity; working long hours; and navigating inaccessible systems and environments. The burden of everyday demands was reflected in quotes such as “the weight [of] day-to-day activities or chores such as cooking meals or doing the dishes”.

In *“The state of the world”: extra-personal events*, participants described feelings of “despair” and “utter powerlessness” at local, national and global events, including the Covid-19 pandemic, politics, the economy, animal welfare and endangerment, climate change, and “injustice”.

##### “Lack of understanding, lack of respect”

Many participants cited instances of being misunderstood, dismissed, invalidated, victimised and/or ostracised, attributing these to *lack* of understanding and/or *disinterest* in understanding. We interpreted three common experiences as sub-subthemes:

In the first, *“Abandoned by [health] services”*, participants described suicidal feelings generated by the absence, inadequacy and inconsistency of healthcare, social care and educational support. Professionals and services were described as underfunded and unequipped to understand, accommodate and support autistic people. While one participant cited the absence of support while “waiting years” for autism assessment, another expressed the disillusionment of thinking that a diagnosis “would unlock help/support/understanding (it didn’t)”.

In the second, *“The immeasurable pain of thinking I’m safe and discovering I’m really unsafe”*, participants described experiences where professionals or services were uncaring or actively antagonistic. The “demeaning” and “cruel” UK benefits system was frequently mentioned, with one participant feeling “interrogat[ed]” and expressing “sadness for… the way you’re looked upon by others”. Others described being disbelieved or “gaslit” by UK healthcare services who did not supply the help they wanted. Some participants described instances where victimisation was condoned or perpetrated by those in positions of authority.

In the third, *“An uneducated and unempathetic society”*, participants described “omnipresent stigma”, “constant negative assumptions”, rejection and victimisation from peers, family, colleagues and society broadly. “Not being believed” or “listened to” were frequently mentioned, as were demanding standards that participants felt they could never meet.

#### Theme 3: Difficult thoughts and feelings

This theme described the mental states underpinning suicidal thoughts and feelings. Some, like guilt, disappointment/regret and dissociation, were relatively infrequent, but we interpreted as subthemes five distinct mental states, sometimes co-occurring and accompanied by hopelessness. Interrelatedness between themes was again strongly evident: for example, participants linked sensory pain (Theme 1) to feelings of “massive disconnect”, being “from another world” and being “trapped”; lack of support (Theme 2) to feelings of burdensomeness; trauma, abuse/assault, daily stressors, and antagonistic relationships with services (Theme 2) to feelings of helplessness and entrapment; being late- or undiagnosed (Theme 4) to feeling broken and different.

##### “Uncertainty of the future”

Some participants suggested fear of future events contributed to their suicidal thoughts. These events included certain eventualities, such as leaving school, parents’ death, fears of their own death, illness or old age, and also less certain eventualities, like job loss due to “being unable to keep up with demands”.

##### “So lonely. Such an outsider.”

Expressing something beyond the pre-specified ‘Loneliness’ item, many comments reflected a deeper and pervasive sense of unbelonging, of being unwanted and different to others. While some comments reflected the perceived absence of relationships and people who cared about them (“I’ve…never been loved by an adult human since my nan died”), others expressed feeling disconnected by (or rejected due to) the fundamental, unending and unsurpassable difference of their neurotype. Feeling like “an alien” and “not fit[ing] into the world” were common expressions.

##### “Too defective to live”

Similarly expressing something beyond the ‘Worthlessness’ survey item, this subtheme captured “all-pervasive” feelings of worthlessness and self-hatred, expressed as being “broken”, “inadequate”, “redundant”, “stupid”, “useless”, “bad”, “a failure at life”. Some participants explicitly expressed being burdensome to others, or that they negatively impacted those they loved (“I seemed to hurt people when I was only trying to be good. It felt better to go”). These statements often co-occurred with expressions of loneliness and disconnection (“I remember each time feeling very alone and as though the world was an alien world I could not understand or navigate and I was only being a burden to others or getting myself into pickles”).

##### “Feeling like my life isn’t going anywhere”

Some participants expressed a lack of meaning or purpose in their lives (“I have no goal and I am useless”), which was sometimes weighed up against the difficulties of living (“The prospect of having to live my life barely scraping by, with no energy or will left to enjoy anything, seems so completely unappealing”).

##### “Deeply overwhelmed” with “no apparent escape”

Participants in this subtheme linked suicidal thoughts, and indeed attempts, to feelings of “overwhelm”, “exhaustion”, and/or being “unable to cope”, a sense of total defeat and entrapment in unbearable circumstances. Suicide was situated by some participants as an (/the only) escape, as a means of “switch[ing] off”, “a reprieve”, “ongoing relief”, or getting “things to stop”. These feelings also occurred in conjunction with the other states (“Feeling as though I don’t fit into this world but knowing I have no choice. I can’t continue to do what’s expected. Feeling that I can’t talk to anybody. Hopelessness”).

#### Theme 4: “I was lost”

Participants frequently referred to being late- or undiagnosed, sometimes implicitly (e.g. expressing confusion about their differences or difficulties), sometimes by making explicit connections between being undiagnosed and feeling different, “broken” and confused, lacking support or receiving inappropriate support, and/or being victimized. Some participants cited the turmoil of receiving their diagnosis (which, for instance, they felt vindicated the cruelty of others) without any post-diagnostic support.

### Part 2: Gender and age differences in factors contributing to suicidal thoughts and feelings

**Figure 3:**
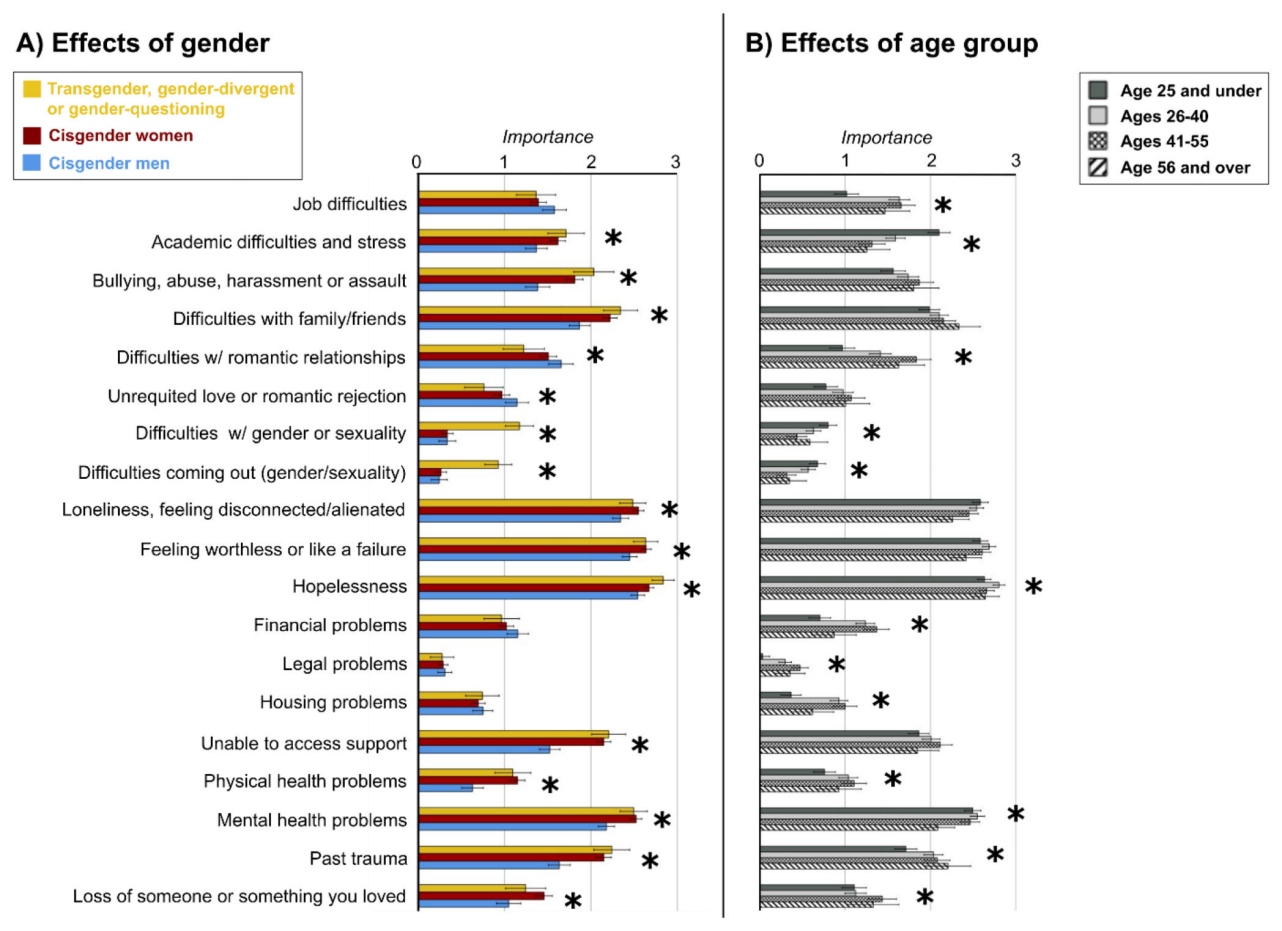
Main effects of gender (A) and age group (B) across contributing factors to suicidal thoughts/feelings *Note*. Part A depicts main effects of gender with asterisks indicating those significant at FDR-corrected p <.05. Part B reflects main effects of age group with asterisks likewise signifying effects significant at FDR-corrected p <.05. Error bars reflect 95% confidence intervals.

Autistic people rated contributing factors as differentially important in their suicidal experiences (F [13.47, 17608.42] = 32.03, p <.001, partial η^2^ =.02). Main effects^8^ showed that autistic people of different genders (F [2, 1307] = 17.29, p <.001, partial η^2^ =.03) and different ages (F [3, 1307] = 9.34, p <.001, partial η^2^ =.02) responded to these items differently. Two-way interactions between contributing factors and Gender (F [26.95, 17608.42] = 12.44, p <.001, partial η^2^ =.02), and factors and Age group (F [40.42, 17608.42] = 9.94, p <.001, partial η^2^ =.02), reflected that the magnitude of group differences differed across items. A significant three-way interaction involving Gender and Age group, due to low numbers, should be cautiously interpreted (see Supplementary Materials, 5). We display planned comparisons for Gender and Age in Figure 3, with statistical notations provided in Supplementary Materials (6).

In summary, cisgender female and transgender groups perceived Academic difficulties and stress, Bullying, Difficulties with friends/family, Hopelessness, Being unable to access support; Physical health issues; Mental health issues, and Past trauma as more important to their STB than did cisgender men. The transgender group was also differentiated from cisgender men by the importance of items related to gender/sexuality, while cisgender women differed from men in the importance of Loneliness, Worthlessness, and Loss/bereavement. Cisgender men rated Difficulties with romantic relationships and Unrequited love/rejection as more important than the other groups. For age, younger participants tended to rate Academic difficulties and gender/sexuality items as *more* important than older groups; in contrast, they tended to rate as *less* important Job difficulties, Difficulties in romantic relationships, Financial, Legal and Housing problems, Physical health problems, and Past trauma. The 26-40 age group rated Hopelessness as more important than other groups.

### Part 3: Contributing factors as predictors of lifetime STB

In the first model, the 9 contributing factors predicted 14% of the variance in lifetime STB (χ^2^(27) = 193.30, p < 0.001; Nagelkerke R^2^ =.14). Variables which significantly differentiated between levels of lifetime STB were high importance of Bullying, Hopelessness, Being unable to get support, Mental health problems, and Past trauma (see Table 2 and Supplementary Materials, 7, for full statistical notations). Participants who had attempted suicide were distinguished from other groups by their greater likelihood of rating Past trauma and Being unable to get support as very important. The importance of Bullying significantly differentiated between those who attempted suicide vs. those with suicide ideation and brief passing thoughts of suicide, but not between individuals with suicide plans and those who had attempted suicide. The importance of Hopelessness and Mental health problems only differentiated between those who had attempted suicide vs. those with only passing thoughts of suicide. These associations were unaffected by the inclusion of gender, age and covariates in the second, adjusted model (Supplementary Materials, 7).

Confirmatory logistic regression corroborated that only the importance of Being unable to access support and Past trauma differentiated between those with and without lifetime suicide attempts (see Supplementary Materials, 8).

## Discussion

Through exploring the self-reported factors underpinning STB, their divergence across age and gender groups, and their ability to discriminate between autistic people with different degrees of lifetime STB, we hoped to advance conceptual understanding of suicide in an autism-specific context. We discuss our findings in light of clinical and policy implications for suicide prevention.

### Contributing factors resemble yet apparently diverge from theory-derived constructs

The centrality of mental ill-health, loneliness/social disconnection, feelings of worthlessness/failure, and hopelessness to STB was consistent with previous accounts from autistic people^32^ and studies using standardised measures of these constructs in autistic populations.^11,12,22,23,53–55^ These items also appear supportive of constructs from psychological theory, including thwarted belongingness and perceived burdensomeness,^6^ conceptually akin to ‘loneliness’ and ‘worthlessness’ in our quantitative data. Both thwarted belongingness and perceived burdensomeness have been linked to STB in autistic people,^10–12,14^ but standardised measures to assess them operate differently in autistic and non-autistic people.^26^ In that we did not directly assess participant perceptions of thwarted belongingness and perceived burdensomeness (or other constructs from psychological theory), we have limited ability to identify differences in their conceptualisation across autistic and non-autistic people. This said, there are clues in our qualitative data which may explain the aforementioned measurement invariance^26^ and indicate differential manifestations and experiences of these two constructs.

Thwarted belongingness and perceived burdensomeness^6^ appear to be reflected in qualitative subthemes, “‘So lonely…’” and “‘Too defective…’” – albeit with additional elements not apparent in the original constructs and tests designed to measure them. A sense of ‘disconnection by difference’ (where our participants felt that their “alienness” and features such as sensory sensitivities caused a “massive disconnect” from others) is not obvious in the original thwarted belongingness construct. Similarly, a sense of chronic, unfavourable comparison with non-autistic others (“feeling ‘less than’ other people”; “incompatible with the expectations of being alive as a human being”), sometimes occurring in the context of being undiagnosed, is not immediately consistent with typical descriptions of perceived burdensomeness as an acute state. The fact that these states were almost always connected by participants to intrapersonal and/or interpersonal experiences *associated with being autistic* is also suggestive of differences in the way these states develop and manifest.

Since previous studies cast doubt on the validity of ITS constructs and their assessment tools in autistic populations,^12^ these findings are useful in suggesting that these constructs *are* highly relevant for autistic people, but would benefit from recontextualization through an autism-informed, trauma-informed and minority stress^9^ framework. Where autism might, for instance, be interpretated within an ITS framework as one of many “risk factors” (like mental illness) for development of these states,^6^ this perspective narrows focus to the pathologized individual, making no demand for social and systemic change. Similarly, the original emphasis on *perceptions* of unbelonging and burdensomeness fails to engage with the reality of implicit and explicit stigmatization, discrimination, victimisation and ostracism of autistic people.^45,56,57^ Given the highlighting of societal and systemic gaps, inequities and injustices as driving factors for STB in both the quantitative and qualitative data, theories and suicide prevention efforts limited to intrapersonal factors and the person’s own interpersonal connections will be less than fully helpful for autistic people.

Beyond the ITS, the qualitative data was supportive of other theoretical approaches relevant for interventions and support. The subtheme ‘“Deeply overwhelmed’…” appeared consistent with the integrated motivational-volitional [IVM] model of suicide,^7^ which situates suicidal thoughts and intentions in feelings of inescapable defeat and/or humiliation, such as might arise from social rejection and social comparison. There is support for this model in autistic adults,^20^ and external entrapment (e.g. pressure to mask) was accompanied and sometimes augmented by internal entrapment (e.g. “sticky” autistic thought processes).^7^ Also compatible with the IVM was subtheme ‘“Feeling like my life isn’t going anywhere”‘, where participants spoke about the *absence* of meaning, enjoyment and purpose to balance out the “difficulty” of living – protective factors, through an IVM lens, which decrease the likelihood of entrapment progressing to suicide ideation.^7^ The subtheme is equally consistent with the three step theory of suicide, which suggests that connections (e.g. to people, goals) tether individuals to living despite psychological pain and hopelessness.^5^ Both models widen our perspective beyond the *presence* of suicidogenic states to the *absence* of protective factors, such as peer support,^58^ which present targets for intervention.

These findings have clinical utility for risk formulation and safety planning with autistic people, highlighting that STB are fluid, complex, and underpinned by inter- and intra-personal factors co-occurring with external stressors. The *interaction* of these elements is essential, where, for instance, traumatic and stressful life experiences common to autistic people might be compounded and augmented by their cognitive, emotional and sensory profile (e.g. ruminative thinking, emotion regulation difficulties), and by isolation. Current UK best practice guidance stresses the importance of full psychosocial assessment to identify risk and protective factors to inform a collaborative safety plan,^59^ with additional guidance available for safety planning with autistic people.^60^ Clinical intervention for STB in autistic people should be similarly informed by the autistic profile and common experiences of this group, including complex trauma and polyvictimization. Understanding and respecting autistic culture^61^ may reduce instances where participants felt unheard or disbelieved in clinical settings.

### Contributing factors to suicide differ by gender and age

We were unable to examine intersectional differences in our qualitative data, but these were evident in responses to pre-specified contributing factors. Mental and physical illness, victimisation, trauma and inability to access support loomed larger in ratings of autistic women and transgender, gender-divergent or gender-questioning participants, corroborating poorer health and greater victimisation and support barriers in these groups.^39–43^ The importance of hopelessness, in the transgender group, corroborated their greater levels of lifetime suicidality, and greater importance of conflicts with friends/family might be linked to the stigma these individuals experience across their lives.^40^ Autistic women similarly highlighted friend/family differences, corroborating previous suggestions that these stressors might be more numerous and impactful for these individuals.^62^ Their greater emphasis on loneliness is consistent with studies reporting greater loneliness in autistic women than men.^46,63^ Notably, other studies find autistic men equally vulnerable to loneliness,^47,64,65^ albeit, perhaps, at different points in their lives.^46^ The focus of our male sample on romantic relationships may indicate greater distress associated with certain kinds of relationships.

While effects of age on academic difficulties, gender/sexuality difficulties, employment, financial, legal and housing concerns, physical health, trauma and bereavement were as expected, the *absence* of differences in ratings for ‘bullying’ and loneliness confirmed the ubiquity of these issues across the lifespan.^45–47^ An unexpected finding, the greater importance of hopelessness among the 26-40 age group might reflect the decline of autism support and services into adulthood,^38^ and/or the continued unfolding of social, economic, and health-related repercussions of earlier life adversity.^66^ Our findings underscore the need for care to be age-, gender- *and* autism-informed, mindful of additional intersectional identities which confer additional suicide risk.

### Differentiating factors between suicidal thoughts and behaviours

Our analysis highlighted five factors which discriminated between participants with different levels of STB. *Lower* perceived importance of hopelessness and mental illness differentiated those with fleeting suicidal thoughts from all other groups, supporting studies linking mental illness to suicidal thoughts *and* behaviour in autistic people.^11,12,22,25,53–55,67–69^ Greater perceived importance of ‘bullying’, difficulties accessing support, and past trauma, however, differentiated participants with higher degrees of lifetime suicidality. While we measured *perceived importance* of life events, thoughts and feelings which participants *might* have experienced rather than the extent of exposure to these factors themselves, the findings suggest these factors may have particular significance in the ideation-to-action trajectory for autistic people.

In neurotypical people, suicide plans are important in eroding fear of death and facilitating the transition to action.^70^ While participants might have had different interpretations of what constitutes a “plan”,^70^ at very least these reflect a progression from passive to active, intentional suicidal ideation,^70^ and possibly greater degrees of mental rehearsal and/or preparatory action. That the importance of bullying distinguished participants with suicide plans and attempts appears supportive of the link between bullying and STB in autistic people (as indeed does our qualitative data).^30,71,72^ Altogether, this highlights victimisation as an essential target for preventing suicidal trajectories.

Greater perceived importance of past trauma and inability to access support were the only variables which predicted suicide attempts. Trauma has been previously linked to suicide attempts in autistic people,^10,14,18,73^ and was a prominent feature of our qualitative data, wherein it was frequently complex, chronic, interpersonal and perpetrated by apparent sources of support. Relatedly, in our qualitative data, inability to access support was mentioned recurrently in expressions of hopelessness, isolation, burdensomeness, and helpless entrapment in intolerable circumstances, as mirrored in other studies.^32,50,74^ While we cannot ascertain participants’ actual experiences, the findings indicate at least that *perceiving* oneself as unable to access help, and possibly feeling more distressed about this, was associated with higher lifetime suicidality. Given the *many* diagnosed and undiagnosed autistic adults not in receipt of services or support,^38,75^ this finding is a urgent call to action for policymakers, corroborating previous recognition that autistic people are a high-risk group who continue to receive insufficient support.^76–78^

### Limitations and future directions for research and policy

Methodological weaknesses include our cross-sectional design: we cannot ratify that “contributing factors”, framed as “predictors” in statistical parlance, causally contributed to or even preceded STB. These analyses show non-directional associations between varying levels of STB and *perceptions* of, rather than exposure to, contributing factors. For example, we cannot ascertain if those who did not rate bullying as highly important never experienced it, were less distressed by the experience, and/or perceived other factors to supersede lasting injuries related to bullying.

While we attempted to protect against fraudulent responses, we were unable to validate autism diagnoses or any information provided. We used a single question about lifetime experiences of suicidal thoughts/plans/attempts, asking participants to reflect on their lifetime history with STB. While autistic participants were presumed to interpret the SBQ-ASC similarly to the validation sample in the original study,^51^ our use of a single item in this retrospective way, generalising across the lifecourse, makes it impossible to link contributing factors to specific instances within the ideation to action trajectory. Since our question about contributing factors was unstandardised, participants may have had different interpretations of the pre-specified factors and/or response options (e.g. what it meant for factors to be “important” vs. “relevant”).

Self-report surveys are unlikely to recruit those individuals who do not want to disclose or seek support for STB. Our highly qualified, female-skewed and white sample is not necessarily representative of autistic men (underrepresented here), people with LD, and/or ethnic minorities. Our findings are culture-bound: since nationality, culture and ethnicity shape perceptions of STB,^79^ autistic people from other countries might respond differently. We emphasise the importance of longitudinal, lifespan approaches to risk and protective factors in diverse autistic samples, and the value of first-hand accounts for our understanding of STB.

Our final reflections concern the policy implications of this work. In the UK, recent recognition of suicide risk in autistic people^77^ has been accompanied by developing guidance for mental health services^76^ and a review of legislation from 2009 which aimed to improve outcomes for autistic people.^78^ We welcome these efforts and their focus on broader outcomes across education, employment and health- and social care, since many of the contributing factors our participants highlighted would be completely preventable by strategies such as providing effective support before and after diagnosis, tackling social isolation and societal stigma, and access barriers to employment and education. Despite promising co-developed approaches in healthcare, employment and other sectors,^80,81^ further research is needed to inform national and international policy efforts to prevent the perfect storm of risk factors and ensure that autistic people have equal access to meaningful connections, autonomy, success, enjoyment and purpose in life.

## Conclusions

Affording a window into how autistic people think about suicide, our findings indicate that the self-reported factors underpinning STB in autistic people, while resembling constructs from psychological theory, are heavily contextualised by autistic experiences and the autistic profile. Perceived contributing factors differed by age and gender, but certain factors, like mental illness, loneliness, hopelessness, and feelings of worthlessness and failure, appeared important to all participants. Over and above this, though, we found that the importance of bullying, trauma and inability to access support appeared to distinguish those with greater degrees of lifetime STB. These findings highlight multiple avenues through which preventative action could prevent the development of suicidal crises, most notably around addressing societal stigma, victimisation, and barriers to support.

## Supplementary Material

Supplementary Materials

## Figures and Tables

**Table 1:** Sample characteristics in relation to lifetime STB

	Only passing thoughts of suicide (n=139)	Suicide ideation without plans or attempts (n=310)	Suicide plans but no attempts (n=399)	At least one suicide attempt (n=521)	Group differences (ANOVA, chi squared)	Overall sample (n=1369)
Average age (SD, range)	37.36 (14.66, 16-74)	35.67 (14.40, 16-79)	37.67 (14.33, 16-89)	35.51 (14.53, 16-79)	*p* =.104	36.36 (14.77, 16-89)
Age groups						
% *25 and under*	30.22	28.71	29.32	32.63	*p* =.221	30.53
% *26 to 40*	26.62	35.81	28.82	33.01		31.78
% *41 to 55*	31.65	23.87	28.32	22.26		25.35
% *56 and above*	11.51	11.61	13.53	12.09		12.34
Gender^d^						
% *Cisgender men*	28.06	28.06	28.07	16.89	***p* <.001**	23.81
% *Cisgender women*	60.43	52.26	49.87	52.40		52.45
% *Transgender, gender- divergent or gender-questioning*	11.51	19.68	22.06	30.71		23.74
Ethnicity						
% *White*	88.49	89.68	88.72	90.40	*p* =.965	89.55
% *With a minority ethnic background or undisclosed*	11.51	10.32	11.28	9.6		10.45
Highest educational attainment						
% *No formal qualifications above GCSEs, high-school diploma or equivalent.*	17.99	24.84	23.06	26.49	***p* =.011**	24.25
% *AS Levels, A Levels,*	9.35	12.26	12.53	18.23		14.32
*Access to Higher*						
*Education or equivalent.*						
% *Diplomas, certificate of higher education, degrees.*	48.20	35.48	35.84	34.55		36.52
% *Postgraduate qualifications.*	22.30	25.16	26.07	18.81		22.72
% *Prefer not to say or did not respond.*	2.16	2.26	2.51	1.92		2.19
Employment status						
% *Any kind of employment or student*	67.63	72.58	72.43	59.31	***p* <.001**	66.98
% *Caregiver or voluntary work*	9.35	4.84	3.51	7.29		5.84
% *Unemployed/unable to work*	17.27	19.03	16.79	28.79		21.91
% *Retired or did not disclose*	5.76	3.55	7.27	4.61		5.26
Autistic status						
% *Formally diagnosed*	58.27	60.65	58.90	70.44	***p* <.001**	63.62
% *Possibly autistic^e^*	41.73	39.35	41.10	29.56		36.38

*Note*. Bolded group differences were significant at a false discovery rate of p <.05.

**Table 2:** Multinomial regression: significant relationships between perceived importance of contributing factors and lifetime suicidality

Contributing factors as predictors of lifetime suicidality						
Contrast with suicide attempts (reference category)	Variables	B (SE)	Wald χ^2^	*p*	OR of being in the suicide attempts group	95% CI for OR
**1. Passing thoughts**	Importance of bullying	.61 (.25)	6.04	.014	1.84	1.13, 2.98
	Importance of hopelessness	1.08 (.26)	16.91	<.001	2.95	1.76, 4.94
	Importance of mental health problems	1.10 (.22)	23.92	<.001	3.00	1.93, 4.66
**2. Suicide ideation** **without plans or** **attempts**	Importance of bullying	.56 (.17)	10.73	.001	1.76	1.25, 2.47
	Importance of being unable to access support	.54 (.16)	10.98	.001	1.72	1.25, 2.38
	Importance of past trauma	.52 (.17)	9.05	.003	1.68	1.20, 2.35
**3. Suicide plans**	Importance of being unable to access support	.56 (.15)	14.32	<.001	1.76	1.31, 2.35
	Importance of past trauma	.47 (.16)	9.25	.002	1.61	1.18, 2.18
**Main effects**	Importance of bullying: χ^2^(3) = 16.43, p <.001					
	Importance of hopelessness: χ^2^(3) = 17.70, p <.001					
	Importance of being unable to access support: χ^2^(3) = 18.88, p <.001					
	Importance of mental health problems: χ^2^(3) = 25.04, p <.001					
	Importance of past trauma: χ^2^(3) = 13.12, p =.004					

*Note*. For brevity, Table 2 displays only significant associations between the perceived importance of some contributing factors to suicidal thoughts and feelings. We present full statistical notations in Supplementary Materials (7).

## Data Availability

The datasets used and/or analysed during the current study are available from the corresponding author on reasonable request.

## Declarations

a “Learning disability” is also known as “intellectual disability” in other countries: reduced ability to understand new or complex information, learn new skills and live independently, characterised by an IQ of 70 or below (DSM-5).

b STB encompasses *suicide ideation*, meaning thoughts about suicide which may be ‘active’ or ‘passive’ (the desire to kill oneself, vs. having the desire to be dead); *suicide plans*, meaning considering the means of attempting and dying by suicide, sometimes including preparatory actions; *suicide attempts*, meaning actions intended to cause death which do not result in the same; and *suicide deaths*, meaning actions intended to cause death which have that fatal result.

c Of all the datasets we received, there were 23 datasets from autistic participants who had never experienced STB. These participants did not therefore complete the questions relevant to this analysis, so we excluded them from the analyses in this paper

6 Unfortunately, we were unable to perform a priori power analyses due to lack of appropriate comparisons in previous literature.

7 Double quotation marks in themes, subthemes and sub-subtheme titles indicate instances where we named these using participants’ own words.

8 There were also significant main effects of educational attainment, current employment, and ethnicity as covariates – see Supplementary Materials 5.

9 By a ‘minority stress framework’, we refer to work conceptualising neurodivergent status as another way in which individuals can experience adversity, inequality and marginalisation related to being a minority in society.^57^ It highlights that autistic people, like other minority groups (e.g. people of colour, individuals with transgender or non-heterosexual identities), experience stigma and stress related to being minorities, and have poorer health in consequence.

## List of abbreviations

STB: Suicidal thoughts and behaviours
LD: Learning (/intellectual) disabilities
ITS: Interpersonal theory of suicide
IVM: Integrated motivational-volitional model of suicide
