# Supplementary material for: “A combination of everything”: a mixed-methods approach to the factors which autistic people consider important in suicidality
Source: Autism Adulthood. Author manuscript; Available in PMC 2025 Oct 31. (PMC7618309; doi:10.1177/25739581251371393)
Supplement: Supplementary Materials [file EMS208783-supplement-Supplementary_Materials.pdf]

**Supplementary materials for**

***"A combination of everything": a mixed-methods approach to the factors which autistic people consider important in suicidality'***

Moseley, R. L., Marsden, S. J., Allison, C. L., Parsons, T. A., Cassidy, S., Procyshyn, T., Pelton, M., Weir, E. M., Chikaura, T., Mosse, D., Hall, I., Owens, L., Cheyette, J. , Crichton, D., Rodgers, J., Hodges, H., & Baron-Cohen, S.

1. Data cleaning process and additional demographic information about the sample
2. Contributing factors to suicidal thoughts and feelings pre-included in the survey: wording, missing data and correlations
3. Statistical notations for logistic regression predicting whether participants provided qualitative data
4. Thematic table for qualitative analysis
5. ANOVA of contributing factors to suicidal thoughts and feelings: statistical notations for covariate effects and three-way interaction
6. Statistical notations for planned comparisons of gender and age effects on contributing factors to suicidal thoughts and feelings
7. Multinomial regression: full statistical notations and replication with covariates
8. Corroborating logistic regression differentiating between participants with and without lifetime suicide attempts

**Supplementary item 1: Data cleaning process and additional demographic information about the sample**

To guard against the threat of fraudulent data, we utilised Qualtrics' fraud detection measures as well as visually examining suspect datasets for meaningful qualitative responses. Qualtrics metrics designed to identify duplicate responses and bots included "Q_BallotBoxStuffing", "Q_RecaptchaScore", "Q_RelevantIDDuplicate", "Q_RelevantIDDuplicateScore", "Q_RelevantIDFraudScore", and "Q_RelevantIDLastStartDate" – the latter four use meta data from participants including their IP addresses and approximate locations (deleted once checked). The survey contained numerous opportunities for free-text responses, including the question about contributing factors to suicidal thoughts and feelings, questions about healthcare experiences and psychological interventions when seeking help for suicidality, questions about ideas for suicide prevention, and opportunity to leave feedback at the end of the survey. Participants who were flagged up by Qualtrics' measures were retained if visual inspection revealed meaningful, sensible responses to one or more of these free-text questions (for instance, recounting their experiences of seeking help for suicidality).

With these measures in place, although 1,525 people began the survey, 156 responses were discarded from analysis. These included datasets from non-autistic people (n=41); datasets which were duplicates or contained abusive or apparently facetious responses indicative of trolling (n=18); datasets deemed to be bots (5 excluded; 16 visually inspected and retained); datasets from participants who completed the survey but reported never experiencing suicidal thoughts, who hence did not answer the relevant questions in the survey (n=23); and datasets from participants who either did not complete question 17, assessing the contributing factors to suicidal thoughts and feelings (n=68), or who did not respond to necessary demographic questions (n=1). The final sample comprised 1,369 participants (almost 94% residing in the

UK), 1216 of whom completed the whole survey. Additional details to those reported in the main text are as follows:

| | Only passing thoughts of suicide (n=139) | Suicide ideation without plans or attempts (n=310) | Suicide plans but no attempts (n=399) | At least one suicide attempt (n=521) | Group differences (ANOVA, chi squared) | Overall sample (n=1369) |
|---|---|---|---|---|---|---|
| Average age (SD, range) | 37.36 (14.66, 16-74) | 35.67 (14.40, 16-79) | 37.67 (14.33, 16-89) | 35.51 (14.53, 16-79) | *p* = .104 | 36.36 (14.77, 16-89) |
| Age groups | | | | | | |
| *% 25 and under* | 30.22 | 28.71 | 29.32 | 32.63 | *p* = .221 | 30.53 |
| *% 26 to 40* | 26.62 | 35.81 | 28.82 | 33.01 | | 31.78 |
| *% 41 to 55* | 31.65 | 23.87 | 28.32 | 22.26 | | 25.35 |
| *% 56 and above* | 11.51 | 11.61 | 13.53 | 12.09 | | 12.34 |
| Gender[1] | | | | | | |
| *% Cisgender men* | 28.06 | 28.06 | 28.07 | 16.89 | ***p* < .001** | 23.81 |
| *% Cisgender women* | 60.43 | 52.26 | 49.87 | 52.40 | | 52.45 |
| *% Transgender, gender-divergent or gender-questioning* | 11.51 | 19.68 | 22.06 | 30.71 | | 23.74 |
| Country of residence | | | | | | |
| *% UK* | 97.84 | 94.52 | 93.98 | 91.55 | *p* = .489 | 93.57 |
| *% USA* | 0.72 | 3.23 | 4.01 | 6.14 | | 4.31 |
| *% Europe* | 0.72 | 1.61 | 1.00 | 1.15 | | 1.17 |
| *% South America* | 0.72 | 0.32 | 0.75 | 0.19 | | 0.44 |
| *% Canada* | 0 | 0.32 | 0.25 | 0.38 | | 0.29 |

[1] Over two questions, participants were asked about their sex assigned at birth (‘male’ [28.05%], ‘female’ [71.66%] or ‘intersex other’ [0.29%]) and their gender identity (where they could choose multiple terms to describe their gender or enter their own response). For analysis purposes, to create sizeable groups within variables, we used these two questions to create a single item, displayed here and referred to hereafter as ‘gender’, categorising participants as cisgender men, cisgender women, or as transgender, gender-divergent or gender-questioning. The transgender, gender-divergent and gender-questioning group included participants who were transgender men (16.62%), transgender women (3.60%), participants who were currently unsure of their gender (29.85%), and those who expressed a range of identities outside the binary (49.85%).

| | | | | | | |
|---|---|---|---|---|---|---|
| *% Australia and Africa* | 0 | 0 | 0.25 | 0.38 | | 0.22 |
| Ethnicity | | | | | | |
| *% White* | 88.49 | 89.68 | 88.72 | 90.40 | *p* = .965 | 89.55 |
| *% Black* | 0 | 0.65 | 0.50 | 0.00 | | 0.29 |
| *% Mixed or multiethnic* | 7.19 | 5.81 | 6.52 | 6.14 | | 6.28 |
| *% Asian* | 2.16 | 1.29 | 1.25 | 1.15 | | 1.31 |
| *% Other* | 3.60 | 1.94 | 1.25 | 0.96 | | 1.53 |
| *% Undisclosed* | 2.16 | 1.29 | 1.00 | 0.58 | | 1.02 |
| Highest educational attainment | | | | | | |
| *% No formal qualifications above GCSEs, high-school diploma or equivalent.* | 17.99 | 24.84 | 23.06 | 26.49 | ***p* = .011** | 24.25 |
| *% AS Levels, A Levels, Access to Higher Education or equivalent.* | 9.35 | 12.26 | 12.53 | 18.23 | | 14.32 |
| *% Diplomas, certificate of higher education, degrees.* | 48.20 | 35.48 | 35.84 | 34.55 | | 36.52 |
| *% Postgraduate qualifications.* | 22.30 | 25.16 | 26.07 | 18.81 | | 22.72 |
| *% Prefer not to say or did not respond.* | 2.16 | 2.26 | 2.51 | 1.92 | | 2.19 |
| Employment status | | | | | | |
| *% Any kind of employment or student* | 67.63 | 72.58 | 72.43 | 59.31 | ***p* < .001** | 66.98 |
| *% Caregiver or voluntary work* | 9.35 | 4.84 | 3.51 | 7.29 | | 5.84 |
| *% Unemployed/unable to work* | 17.27 | 19.03 | 16.79 | 28.79 | | 21.91 |
| *% Retired or did not disclose* | 5.76 | 3.55 | 7.27 | 4.61 | | 5.26 |
| Autistic status | | | | | | |

| | | | | | | |
|---|---|---|---|---|---|---|
| *% Formally diagnosed* | 58.27 | 60.65 | 58.90 | 70.44 | ***p* < .001** | 63.62 |
| *% Possibly autistic*[2] | 41.73 | 39.35 | 41.10 | 29.56 | | 36.38 |
| Co-occurring conditions | | | | | | |
| *% ADHD* | 20.14 | 17.10 | 16.29 | 24.76 | ***p* = .006** | 20.09 |
| *% Anxiety* | 59.71 | 62.58 | 67.92 | 74.28 | ***p* < .001** | 68.30 |
| *% Depression* | 46.04 | 59.03 | 68.67 | 81.00 | ***p* < .001** | 68.88 |
| *% Eating disorder* | 7.91 | 11.61 | 13.53 | 24.76 | ***p* < .001** | 16.80 |
| *% OCD* | 6.47 | 8.39 | 10.03 | 14.20 | ***p* = .012** | 10.88 |
| *% Personality disorder* | 0.72 | 5.16 | 4.76 | 21.11 | ***p* < .001** | 10.66 |
| *% PTSD or complex PTSD* | 8.63 | 13.87 | 17.54 | 37.04 | ***p* < .001** | 23.23 |
| *% Sensory processing disorder* | 7.91 | 4.52 | 8.52 | 14.01 | ***p* < .001** | 9.64 |
| *% Specific learning difficulty* | 10.07 | 10.65 | 14.79 | 20.15 | ***p* < .001** | 15.41 |

*Note*. Bolded group differences were significant at a false discovery rate of $p < .05$.

Additional details for the suicide attempt group were that the majority had attempted suicide more than once: 24.5% reported two attempts; 41.5% had attempted three or more times. Asked about the recency of their last suicide attempt, 32.1% had last attempted suicide within the past 2 years; 18.1% had last attempted between 2-5 years ago; 19.1% had last attempted between 5-10 years ago; the remainder reported their last suicide attempt as more than 10 years ago.

[2] The possibly autistic group included those who identified as autistic (37.15%) and those awaiting assessment (62.85%).

**Supplementary item 2: Contributing factors pre-specified in the survey: wording, missing data and correlations**

Contributing factors were based on suggestions from an advisory panel of autistic people (n=13) and family members of autistic people (n=9), and by existing literature on autism and suicide broadly (which highlighted employment problems and unmet support needs[1]; financial hardships and changing life circumstances, such as in housing[2]; interpersonal conflict or misunderstandings[2]; bullying[3]; trauma[4]; feeling like a failure[5], feelings of worthlessness[6,7], loneliness[8] and relatedly, thwarted belongingness[6]). Below is the precise wording of contributing factors listed in the survey and, in red, the number of participants (out of 1369) who rated each one.

1. Job difficulties (e.g. unemployment, job loss, job stress) *(Rated by 1359 participants)*
2. Academic difficulties or stress (e.g., at school, college or university) *(Rated by 1360 participants)*
3. Bullying, abuse, harassment or assault (physical and/or verbal) *(Rated by 1358 participants)*
4. Difficulties in relationships with family/friends *(Rated by 1359 participants)*
5. Difficulties in relationships with romantic partners *(Rated by 1358 participants)*
6. Unrequited love or romantic rejection *(Rated by 1356 participants)*
7. Difficulties understanding or accepting your gender identity and/or sexuality *(Rated by 1355 participants)*
8. Difficulties coming out to others in relation to your gender identity and/or sexuality *(Rated by 1355 participants)*

9. Loneliness or feeling disconnected/alienated from others *(Rated by 1365 participants)*
10. Feeling worthless or like a failure *(Rated by 1362 participants)*
11. Hopelessness *(Rated by 1360 participants)*
12. Financial problems (e.g., poverty, debt) *(Rated by 1353 participants)*
13. Legal problems (e.g., being taken to court) *(Rated by 1351 participants)*
14. Housing problems (e.g., worrying about or being evicted, homelessness) *(Rated by 1357 participants)*
15. Being unable to access support you needed *(Rated by 1355 participants)*
16. Physical health problems *(Rated by 1359 participants)*
17. Mental health problems *(Rated by 1362 participants)*
18. Trauma from past events *(Rated by 1357 participants)*
19. Losing a person, animal or something you loved (e.g., through bereavement or separation) *(Rated by 1357 participants)*

See below for nonparametric associations between importance ratings for each contributing factor.

*Correlation key:*

-1 0 1

**Nonparametric correlations between importance ratings ($r_s$, $p$):**

| | 1 | 2 | 3 | 4 | 5 | 6 | 7 | 8 | 9 | 10 | 11 | 12 | 13 | 14 | 15 | 16 | 17 | 18 |
|---|---|---|---|---|---|---|---|---|---|---|---|---|---|---|---|---|---|---|
| 1. Job difficulties | | | | | | | | | | | | | | | | | | |
| 2. Academic difficulties/stress | .15, < .001 | | | | | | | | | | | | | | | | | |
| 3. Bullying | .14, <.001 | .23, <.001 | | | | | | | | | | | | | | | | |
| 4. Friend/family relationships | .06, .032 | .13, <.001 | .33, <.001 | | | | | | | | | | | | | | | |
| 5. Romantic relationships | .17, <.001 | -.07, .009 | .17, <.001 | .34, <.001 | | | | | | | | | | | | | | |
| 6. Unrequited love | .12, <.001 | .01 .831 | .11, <.001 | .23, <.001 | .52, <.001 | | | | | | | | | | | | | |
| 7. Accepting gender/sexuality | -.07, .008 | .18, <.001 | .12, <.001 | .11, <.001 | -.02, .437 | .02, .415 | | | | | | | | | | | | |
| 8. Coming out to others | -.06, .023 | .16, <.001 | .15, <.001 | .12, <.001 | -.02, .545 | .01, .861 | .71, <.001 | | | | | | | | | | | |
| 9. Loneliness | .00 .986 | .15, <.001 | .16, <.001 | .31, <.001 | .13, <.001 | .20, <.001 | .17, <.001 | .14, <.001 | | | | | | | | | | |
| 10. Worthlessness | .18, <.001 | .19, <.001 | .16, <.001 | .25, <.001 | .13, <.001 | .13, <.001 | .10, <.001 | .04, .105 | .40, <.001 | | | | | | | | | |
| 11. Hopelessness | .12, <.001 | .12, <.001 | .15, <.001 | .20, <.001 | .11, <.001 | .10, <.001 | .07, .008 | .07, .015 | .37, <.001 | .50, <.001 | | | | | | | | |
| 12. Financial problems | .43, <.001 | .11, <.001 | .18, <.001 | .20, <.001 | .26, <.001 | .17, <.001 | .03, .285 | .05, .047 | .10, <.001 | .17, <.001 | .17, <.001 | | | | | | | |
| 13. Legal problems | .13, <.001 | .07, .017 | .14, <.001 | .16, <.001 | .24, <.001 | .13, <.001 | -.04, .179 | -.02, .444 | -.03, .303 | .08, .006 | .06, .045 | .28, <.001 | | | | | | |
| 14. Housing problems | .34, <.001 | .07, .006 | .22, <.001 | .21, <.001 | .19, <.001 | .14, <.001 | .02, .554 | .06, .041 | .04, .147 | .09, <.001 | .12, <.001 | .59, <.001 | .34, <.001 | | | | | |
| 15. Being unable to get support | .12, <.001 | .24, <.001 | .29, <.001 | .23, <.001 | .03, .217 | -.03, .296 | .11, <.001 | .14, <.001 | .19, <.001 | .21, <.001 | .28, <.001 | .27, <.001 | .20, <.001 | .31, <.001 | | | | |
| 16. Physical health problems | .21, <.001 | .15, <.001 | .25, <.001 | .18, <.001 | .15, <.001 | .07, .010 | .09, <.001 | .11, <.001 | .08, .003 | .10, <.001 | .11, <.001 | .32, <.001 | .22, <.001 | .30, <.001 | .34, <.001 | | | |
| 17. Mental health problems | .08, .002 | .19, <.001 | .12, <.001 | .14, <.001 | .06, .031 | .05, .076 | .10, <.001 | .11, <.001 | .22, <.001 | .26, <.001 | .32, <.001 | .12, <.001 | .05, .054 | .07, .017 | .34, <.001 | .21, <.001 | | |
| 18. Past trauma | .08, .003 | .09, .002 | .43, <.001 | .37, <.001 | .22, <.001 | .14, <.001 | .10, <.001 | .15, <.001 | .18, <.001 | .20, <.001 | .23, <.001 | .23, <.001 | .19, <.001 | .24, <.001 | .36, <.001 | .30, <.001 | .30, <.001 | |
| 19. Loss or bereavement | .11, <.001 | .10, <.001 | .23, <.001 | .21, <.001 | .25, <.001 | .25, <.001 | .03, .217 | .05, .065 | .10, <.001 | .12, <.001 | .12, <.001 | .19, <.001 | .18, <.001 | .20, <.001 | .24, <.001 | .30, <.001 | .19, <.001 | .34, <.001 |

**Supplementary item 3: Statistical notations for logistic regression predicting whether participants provided qualitative data**

To understand who among our sample provided qualitative data on the factors contributing to their suicidal thoughts and feelings, we performed a logistic regression predicting whether (1) or not (0) participants provided qualitative data. Predictors were gender (with cisgender men as reference category); age, treated herein as a continuous variable; lifetime suicidality, treated as an ordinal variable; as well as covariates ethnicity (white status as the reference category), autistic status (possibly autistic as the reference category), educational attainment (postgraduate qualifications as the reference category), and current employment (retired/undisclosed as reference category).

These predictors explained 6% of the variance in the dependent variable ($\chi^2(13) = 60.75$, $p < 0.001$; Nagelkerke $R^2 = .06$). Although there were no significant effects of autistic status ($p = .078$), ethnicity ($p = .123$), highest educational attainment ($p = .425$) or current employment ($p = .328$), older participants were more likely to provide qualitative input ($B = .02$ [SE: .01], $\chi^2 = 28.52$, $p <.001$; OR = 1.03 [CI: 1.02, 1.03]). For gender, transgender, gender-divergent or gender-questioning participants were more likely than cisgender men to provide qualitative input ($B = .43$ [SE: .18], $\chi^2 = 5.59$, $p = .018$; OR = 1.54 [CI: 1.08, 2.20]). Women tended towards greater likelihood of providing qualitative input than cisgender men ($B = .28$ [SE: .15], $\chi^2 = 3.74$, $p = .053$; OR = 1.33 [CI: 1.00, 1.76]). Higher levels of lifetime suicidality (where 1 = passing thoughts, 2 = suicide ideation without plans, 3 = suicide plans but no attempts, 4 = suicide attempts) were associated with greater likelihood of providing qualitative input ($B = .18$ [SE: .06], $\chi^2 = 9.20$, $p = .002$; OR = 1.20 [CI: 1.07, 1.35]).

## Supplementary item 4: Thematic table

Quotations for each theme and subtheme are included below, with any possibly identifying individual details/information are removed.

Theme 1, Neurodivergence, mental and physical health. > Subthemes 'Autism-related challenges', 'Other neurodevelopmental, mental and physical health conditions'
Theme 2, Stress and adversity > Subthemes 'Big changes', 'Trauma, abuse and/or assault', 'Ongoing and/or chronic stressors', 'Lack of understanding, lack of respect'
Theme 3, 'Difficult thoughts and feelings' > Subthemes 'Uncertainty of the future', 'So lonely. Such an outsider', 'Too defective to live', 'Feeling like my life isn't going anywhere', 'Deeply overwhelmed' with 'no apparent escape', 'Other difficult thoughts and feelings'
Theme 4: 'I was lost'

| Themes | Subthemes | Sub-subthemes | Quotations |
|---|---|---|---|
| Theme 1: Neurodivergence, mental and physical health | Autism-related challenges<br><br>*This subtheme reflects instances where participants explicitly named or illuded to aspects of being autistic which contributed to their suicidal thoughts and feelings.* | "My brain shuts down"<br><br>*As factors contributing to suicidal thoughts and feelings, this sub-subtheme describes sensory and emotional challenges, meltdowns, shutdowns and burnout.* | • "A lot of uncertainty, moving homes, schools, being separated from family for a long time, feeling unloved by family, ***feeling confused over low feelings***"<br>• "Sexual assault, peers didn't believe me and abandoned me. ***Severe meltdown***/flashback"<br>• "When I become ***overwhelmed with anxiety for too long a period of time it feels like it flips a switch in my brain and my brain shuts down*** for long periods of time. This makes it hard to function."<br>• "***Primarily sensory overwhelm***. I needed noise cancelling headphones and assistance but my parents did not know I was autistic. They tried their best but their attempts at helping nearly killed me."<br>• "Not taking care of my health (e.g. not sleeping or eating properly), which led to ***massive problems with my emotional regulation."*** |

| | | | |
|---|---|---|---|
| | | | • "Having no actionable plan to escape ***relentless overwhelm of sensory*** and social experience which would also ensure ongoing relief. As a teenager not knowing how to unmask - suicide only option."<br>• "Recurrent exposure to ***poor sensory environments***"<br>• "Masking and ***Delayed emotional processing***. People asking me if I'm ok including doctors and saying yes, but ***the emotions hitting me 45 mins to hours later***."<br>• "***Burnout*** and feeling unable to deal with the complicated world I live in."<br>• "Also, ***Experiencing/ having Acute sensory afflictions brings with it challenges beyond anyone's understanding,*** causing a massive disconnect within society, but not nature. I feel I'm from another world"<br>• "***Burn out*** and decrease in functioning/ trying to carry out daily activities."<br>• ***"Having lots of intense emotions. Having Big emotions.*** Needing things to stop. Needing a brake from me. Injustice's. Feeling unfixable."<br>• "***Becoming overwhelmed particularly emotionally*** often following contact with services (cmht, camhs, social services) when they do r understand my needs/how to communicate" |

| | | | |
|---|---|---|---|
| | | | • "Wanting the feeling of constant ***burn out*** to just stop."<br>• "Helplessness: ***under constant sensory assault*** by several other degraded people, being 5 years set aside in a residence for the concentration of discarded by-products ("supported living")"<br>• "***no physical space to escape sensory overload: perfume and laundry fragrances of neighbours pushing through closed doors, traffic noise,*** constant seizure triggering, no sleep, trapped inside."<br>• "***Prolonged Autistic Burnout*** and lack of social/emotional support have played a huge part."<br>• "Autistic burnout"<br>• "Life not really having time to pause / stop and feeling left behind due to ***burnout***"<br>• "Burn out"<br>• "Exhaustion"<br>• "Chronic exhaustion"<br>• "***Not understanding why i was feeling what i was feeling. (Very important).*** Constent stress and overwhelm over many years. (Very important). Getting the wrong help. (Very important)"<br>• "***Burnout*** / exhaustion, alcoholism, forced drug use"<br>• " Not being able to live to society standards in many ways ***not being able to tolerate the world sensory***" |

| | | | |
|---|---|---|---|
| | | | • "Spending my whole life masking my autistic traits to survive but then not being believed when I ask for help later on after ***burn out***. Not getting help, being ignored, nowhere to go."<br>• "Side effect and withdrawal associated with psychiatric medication. ***Being in a meltdown or a shutdown. Burnout"***<br>• "Over-using alcohol to try to cope with feelings."<br>• "Not knowing I was autistic/had autism. No interest in future. ***not make sense of emotion's, identify or explain. Made sense via a positive / negative boundary of feeling sick vs not feeling sick."***<br>• "Sensory overload"<br>• "Finding society ***overwhelming sensory wise***: too loud, bright, etc and having trouble keeping up with the fast pace of life."<br>• "***Being unable to deal with heat, smells, noises, lack of peace, lack of head space, and low energy***. Awareness of consequences in old age of not being able to have children."<br>• "Being forced into environments which trigger ***sensory processing issues"***<br>• "Miscommunication, loneliness, ***unprocessed feelings***, no help"<br>• "Even if times are hard, it is not life events, but ***being angry at myself for not being*** |

| | | | |
|---|---|---|---|
| | | | ***able to control or manage my low mood*** that causes suicidal feelings”<br>• “Sexual abuse from age 5. Parents unaware. Parents therefore thought I was dramatic/hysterical. I ***didn’t understand my feelings*** or what the abuse was so thought I was indeed “misbehaving”. Confusion.”<br>• “***Being so sensitive to the energy around me I can feel every mood in the room***. Especially negative and it is a lot to process when I feel like the negative moods people are in is because of me somehow”<br>• “Having emotions that I didn't understand and not knowing how to make them go away.”<br>• “Inability to identify my own emotions.”<br>• “Inability to get out of autistic burnout.” |
| | | “Having to lie to get by”<br><br>*As a factor contributing to suicidal thoughts and feelings, this sub-subtheme reflects masking and its psychological and social impacts on how participants feel and how other people treat them.*<br><br>Back to the top | • “Breakdown in family communication due to my failure to respond. Moved away from home, felt stranded and as if I had abandoned family. ***Partner’s family high expectations due to masking.”***<br>• ***“Having to mask, identity crisis, feeling like I always have to lie to get by*** - lack of understanding of autism and people constantly thinking it's the autistic person that has to change”<br>• “***Spending my whole life masking my autistic traits to survive*** but then not being believed when I ask for help later on after burn out. Not getting help, being ignored, nowhere to go.” |

| | | | |
|---|---|---|---|
| | | | • "Having no actionable plan to escape relentless overwhelm of sensory and social experience which would also ensure ongoing relief. As a teenager ***not knowing how to unmask -*** suicide only option."<br>• "We have a society uneducated and unempathic towards autistic people. ***Consistently masking everytime I go out in public is exhausting and makes you feel like you're consistently playing a character."***<br>• "***Constant emotional exhaustion from having to mask my autistic traits every waking moment*** and still being viewed negativity despite all my efforts. I just wanted a reprieve."<br>• "Trying to live up to other peoples expectations, who I am was not acceptable when I was growing up so ***I learned very quickly to mask to fit in."***<br>• "***Masking*** and Delayed emotional processing. People asking me if I'm ok including doctors and saying yes, but the emotions hitting me 45 mins to hours later."<br>• "People in my life who will not accept diagnosis if I did get one, causing me to ***mask more*** and be in a spiral of despair"<br>• "Masking all day long" |
| | | "Spiralling thinking":<br><br>*This sub-subtheme describes how dichotomous, perseverative and* | • ""Large life change, moving job. Moving house, moving in with partner. ***Losing special interest in the transition***" |

| | | | |
|---|---|---|---|
| | | *intrusive thoughts and executive dysfunction contributed to suicidal thoughts and feelings. Quotes included in this subtheme also indicate inflexible adherence to moral standards; suicide as a logically reasoned decision; fixated interests on death and suicide; and conversely, the importance of being able to engage with passions.*<br><br>Back to the top | • "***Obsessive and intrusive thoughts***, difficult changes in life"<br>• "Unexplained breakup led to complete loss of purpose & ***worst case scenario reasoning*** strained relationships with everyone else. Couldn't speak to her so didn't want to speak to anyone else."<br>• "My father's suicide at age 47 unlocked these possibilities. Earlier I've never visualised suicides so vividly but with his death I started to have ***intrusive thoughts*** of various scenes."<br>• "basic daily tasks/ ***executive challenges***"<br>• "***Going against my values*** (breaking a strike), due to financial worry."<br>• "I breached my own ethical code"<br>• "Feeling as though I am generally a failure in social settings. ***Replaying conversations obsessively*** to try and understand why I don't fit in."<br>• "Being ***Denied access to my special interest***"<br>• "***Can just come on, almost unrelated to life experiences at the time***. Thought everyone felt this way. checked in with myself and thought not to worry as not carefully planned. Feeling passes."<br>• "the first time i experienced it it seems that i had a ***special interest on the afterlife that developed into a hyperfixation***. i wanted to see what a people would experience after death" |

| | | | |
|---|---|---|---|
| | | | <ul><li>"Having problems that I can see the solution to but can't influence that decision to be made. ***Spiraling thinking chaotic thoughts."***</li><li>"Non-romantic ***obsession*** with a person."</li><li>"A sense that no longer existing might be a ***rational solution*** to the problems inherent in existing"</li><li>"I have got used to a ***background noise of suucidal thought, it is almost a rhythm to rumination and loop thinking***. I learned how to put in a circuit break and live with it, I mostly feel good now."</li><li>"Wanting to go to Heaven"</li><li>"My most frequent wish is to die before I get much older."</li></ul> |
| | | "Being unable to understand the world"<br><br>*This sub-subtheme describes contributions of difficulties autistic people experience in trying to communicate with others: initiating or maintaining contact, verbalising their thoughts, emotions and experiences; and understanding the (social) world around them.*<br><br>Back to the top | <ul><li>"Breakdown in family communication due to ***my failure to respond***. Moved away from home, felt stranded and as if I had abandoned family. Partner's family high expectations due to masking."</li><li>"***Inability to verbalise*** abuse that was happening. Not being aware it was important to tell someone or that it was wrong."</li><li>"Relating to other people - Very important"</li><li>"Not knowing what is happening in important situations. That can have serious outcomes."</li></ul> |

| | | | |
|---|---|---|---|
| | | | • “Social anxiety: ***unable to communicate properly***, social isolation, being made fun of”<br>• “Not being able to differentiate if someone has my best interests at heart or what they are thinking about me.”<br>• "being unable to understand the world in general”<br>• “Complete lack of support and support being taken away from me for ‘not engaging’ but they don’t have any understanding of autism at all. ***I am non verbal*** and they don’t take that into account” |
| | | “A relentlessly stressful and unpleasant experience”:<br><br>*This sub-subtheme denotes other instances where participants linked their suicidal thoughts and feelings to other or unnamed aspects of being autistic.*<br><br>Back to the top | • “The lived experience of autism is to feel like you are being gaslit by society, constantly questioning your sense and perspectives. ***The trauma of my brain injury combined with autism reinforces this.”***<br>• ***“Having autism*** and individuals with autism usually have mental health issues because they (including me) have a very serious problem relating to DNA and genetics.”<br>• “Feeling as if I want to re-start my life as a non autistic person.”<br>• “Life with autism has been a relentlessly stressful and unpleasant experience. I only feel ok when I have quiet and solitude.” |

| | | | |
|---|---|---|---|
| | Other neurodevelopmental, mental and physical health conditions<br><br>*As contributing factors to suicidal thoughts and feelings, participants referred to co-occurring neurodivergence, mental and physical health conditions or related difficulties (such as medication side effects, sleep disturbance, substance addiction, self-harm and body image disturbance).*<br><br>Back to the top | | • "Undiagnosed AuDHD pubescent child starting at elite private school on scholarship, no accommodations, with undiagnosed neurodivergent single mother on low income constantly criticising ***(& chronic pain)"***<br>• ***"Social anxiety:*** unable to communicate properly, social isolation, being made fun of"<br>• "High social and general anxiety"<br>• "no physical space to escape sensory overload: perfume and laundry fragrances of neighbours pushing through closed doors, traffic noise, ***constant seizure triggering, no sleep***, trapped inside."<br>• "Side effects from medication"<br>• "***Side effect and withdrawal associated with psychiatric medication***. Being in a meltdown or a shutdown. Burnout"<br>• "General struggles especially socially, and dealing with ***mental health . I have drug resistant depression which I have been diagnosed with since 14***."<br>• ***"I had trouble sleeping***. People weren't listening to me when I tried to explain the issues I was having difficulty with."<br>• "parents divorce, parents mental health, ***the depression genes***, seasonal, stress/overwhelmed. i constantly feel like im in a rush just  to keep up"<br>• "I had a full mental breakdown in 2018 following stress and recurrent of my ***PTSD***. |

| | | | |
|---|---|---|---|
| | | | It led to a month of being sure I would kill myself. Previous situations were only ideations but at that time I was sure”<br>• “Gender dysphoria, and having to wait years to get gender affirming care”<br>• “***Psychosis***, teenage hormones and trauma”<br>• “Domestic abuse, partner with mental health, exhaustion, ***self harm”***<br>• ***“PMDD”***<br>• “ Gender dysphoria. Inability to manage or treat fairly severe ADHD (medically or otherwise)”<br>• ***“Being academically intelligent but have a learning difficulty with Maths.*** No support with bullying at work. Had reprisals due to reporting it.”<br>• “I believe ***drugs*** enhanced my feelings of loneliness and hopelessness as well as suicidal thoughts.”<br>• “difficulty managing day to day life, ***issues around food***, medical and psychiatric abuse, ***gender dysphoria***”<br>• “***ADHD Medication Shortages***. No control over stressful big changes esp. sudden change. Uncertainty anxiety inc. waiting for important decisions to be made by others, inconsistent/unpredictable support”<br>• “***Gender Dysphoria making existing difficult***. Constantly being uncomfortable. Not being able to get help to be happy.” |

| | | | |
|---|---|---|---|
| | | | • "Feeling of unable to cope and overwhelm and ***feeling unwell with fatigue"***<br>• "Being falsely accused of intending to access obscene material via corporate internet access - the material was erotic but had already been broadcast on C4! ***Having suspected prostate cancer."***<br>• "Partner infidelity over decades suddenly coming to light- this was extremely traumatising after a shared trauma, not just "difficulty with romantic partner". ***SUDDEN collapse of mental &bodily health"***<br>• "The lived experience of autism is to feel like you are being gaslit by society, constantly questioning your sense and perspectives. ***The trauma of my brain injury combined with autism reinforces this."***<br>• "Covid climate change ***pmdd***"<br>• "I am writing in this just to say I couldn't think of anything outside of those categories, although quite a few of those questions intersect, for example my ***mental health issues*** stem from trauma."<br>• "2005 Following a friend's suicide. 2015 ***During episode of severe depression***. 2020 In abusive relationship during lockdown"<br>• "***Chronic nightmares*** (trauma-related) meaning ***I never got a rest/break***" |

| | | | |
|---|---|---|---|
| | | | • "People (NHS) not believing anything I said + ***very bad reaction to medication especially SSRIs and anti psychotics"***<br>• " just complete overwhelm from life. Especially after an ***adhd high"***<br>• "My concerns around the climate crisis and governments inability to do anything about it have significantly affected my ***mental health***. This is now something I think about everyday. Also ***PMDD***!"<br>• "In particular, my ***severe OCD*** which I had to get intensive care for. Also, trauma from being exposed to something I should never have seen as child (child porn)"<br>• "***Addiction to self harm***, loosing my independence due to ***debilitating OCD*** for a time and needing to depend on others"<br>• "***Chronic pain/declining health***/fear of getting ill"<br>• "I didn't have difficulties accepting my gender or sexuality, but it was the ***crippling dysphoria*** mixed with the child abuse that was most influential."<br>• "Anxiety based eating disorder which I now believe to be ARFID."<br>• "Feeling like I wasn't a real person. This is ***mental health*** but also people's prejudice."<br>• "These factors stack up. I don't remember being truly happy since very early childhood. I'm certain I have ***complex PTSD*** from trauma but I don't have a diagnosis or support with it." |

| | | | |
|---|---|---|---|
| | | | • "***I have severe OCD***. I also had to leave school very early due to my ASD & physical health because the school didn't know how to accommodate me. One parent was abusing my other parent."<br>• "The effects of child abuse by birth family that used my autism as an excuse to scapegoate me leading to a serious ***dissociative disorder***."<br>• "Not being able to fully understand my ***mental health symptoms and being scared by psychosis***. Trouble explaining to proffessionals. Being dismissed/gaslit. Child abuse being minimised/overlooked."<br>• "coming to terms with sexual abuse, trying to cope with ***dysphoria and eating/self harm issues***"<br>• "***Chronic fatigue***, stress, bullying at school and then work, no access to support for mental health and waiting years for autism assessment"<br>• ***" Health decline over many years making life more and more difficult to endure. Pain and suffering is constant. There is continued torment in every moment of every day.*** Loss of 'self' also."<br>• "Pain from migraines"<br>• "Genetic cardiac problems"<br>• "the birth of my first child may have triggered my first ***psychotic episode***"<br>• "Withdrawal from Venlafaxine" |

| | | | |
|---|---|---|---|
| | | | • "PTSD"<br>• "***Delusion*** that I was living in hell"<br>• "I didn't fit in at school and I was in an ***immense amount of physical pain*** due to growing too quickly and not being given the proper diagnosis/meds by doctors as they just thought it was growing pains"<br>• "Before ASD diagnosis the ***ever increasing doses of mental health medication - horrendous side effects and worse withdrawal.*** Not acknowledged by relevant medical professionals who just kept prescribing."<br>• "***Was during taking fluoxetine, had massive paradoxical effect on me***, turned me into a zombie I couldn't deal with anything. Lost the will to live, no emotion. Plus other side effects."<br>• "Having incorrect medical advice pushed on me by family because my physio missed that I had ***more issues*** than they realized and the exercises I was given made the ***issues worse***."<br>• "Not understanding how ***PTSD effects your whole life*** not just ***nightmares*** at night"<br>• "im ***borderline*** so i can pretty much go from perfectly fine to trying to burn a hole through my chest in a day. it's a nightmare but its better now"<br>• "Managing ***ADHD*** alongside autism (while not knowing I had the condition until about |

| | | | |
|---|---|---|---|
| | | | 22). The feeling like I'm behind my peers in terms of mental”<br>• “***Depression*** kept coming back every time I thought I'd solved it and moved on. It made me feel hopeless about the future.”<br>• “Social anxiety with non-relatives/ peers”<br>• “***Undiagnosed PTSD*** - treatments for other disorders not helping or making me feel worse (due to triggers) and feeling like a failure and its my fault”<br>• “***Depression*** was the dominant factor. Feeling that everyone would be better off if I wasn't around.”<br>• “ Not having any 'trigger' or cause for my ***depression*** until being late diagnosed with autism and, sometime after, that that was the primary cause.”<br>• “Severe eating disorder, didn't know how to stop”<br>• “ Not understanding my neurodivergence, not recognizing that my ***mental health was in crisis*** until I hit the bottom”<br>• “Severe OCD”<br>• “I was treated for ***severe depression, developing into anxiety.*** I was on long term sick leave and had been subjected to unfair treatment. My daughter had just been diagnosed ASD. I was but unaware of it”<br>• “Having autism and individuals with autism usually have ***mental health issues because they (including me) have a very serious problem relating to DNA and genetics***.” |

| | | | |
|---|---|---|---|
| | | | • “Insomnia and agoraphobia”<br>• “disability”<br>• “psychosis”<br>• “Difficulties with acceptance of mental health diagnosis.”<br>• “Not taking care of my health (e.g. not sleeping or eating properly), which led to massive problems with my emotional regulation.”<br>• “Over-using alcohol to try to cope with feelings.”<br>• “Addiction both drugs and alcohol”<br>• “Addiction issues are very important”<br>• “Being noticeably much more intelligent than the other humans but not being able to use it to achieve anything, and ***not being able to stop smoking cannabis (addicted for 20 years)”***<br>• “Burnout / exhaustion, ***alcoholism***, forced drug use”<br>• “Alcohlism/addiction although I have been sober a long time and became suicidal in my sobriety although the attempts were when I was not sober”<br>• “I was addicted to alcohol in a couple of times I attempted suicide”<br>• “unsatisfaction to the physical appearence ( e.g. weight, skin, face looking)”<br>• "The way I look”<br>• “Body dysmorphia, bulimia” |

| | | | |
|---|---|---|---|
| | | | • "In addition to the above, simply the physical sensation of being alive, of having a physical existence in this world"<br>• "Feeling like I hated my body and it didn't represent who I am- feeling disconnected from my physical form."<br>• "Symptoms of hopelessness got worse as I got older and I kept going through ***breakdowns***. That was before my diagnosis at the age of 51" |
| Theme 2: Stress and adversity | "Big changes": Life events, changes and transitions<br><br>*This subtheme reflects major life events and transitions (social and biological) which contributed to suicidal thoughts and feelings.*<br><br>Back to the top | | • "***the birth of my first child*** may have triggered my first psychotic episode"<br>• "***parents divorce,*** parents mental health, the depression genes, seasonal, stress/overwhelmed. i constantly feel like im in a rush just to keep up"<br>• "Parents splitting up"<br>• "The points where I have attempted suicide were usually when ***big changes going on*** and I just couldn't cope. I still get very low thoughts and consider it but don't action, this is when too overwhelmed"<br>• "Psychosis, ***teenage hormones*** and trauma"<br>• "Change"<br>• "Menopause"<br>• "Obsessive and intrusive thoughts, ***difficult changes in life***"<br>• "***Large life change, moving job. Moving house, moving in with partner.*** Losing special interest in the transition" |

| | | | |
|---|---|---|---|
| | | | • "Parenthood"<br>• "***A lot of uncertainty, moving homes, schools, being separated from family*** for a long time, feeling unloved by family, feeling confused over low feelings"<br>• "Thinking everything would get better after school/bullying and I would be a new happy and confident person ***once I went to university***. But I was still having problems and didn't understand why."<br>• "being overwhelmed! I had j***ust left home, studying for A levels,*** very little money, no idea why I couldn't do the things other people did. socially isolated. childhood physical abuse."<br>• "Undiagnosed AuDHD ***pubescent child starting at elite private school on scholarship***, no accommodations, with undiagnosed neurodivergent single mother on low income constantly criticising (& chronic pain)"<br>• "Problems with visa application and being separated from husband while he was in hospital"<br>• "Relocation aged 16"<br>• "Change of school and house"<br>• "Specifically the DWP universal credit people bludgeoning their agenda and not caring to listen. Year of extra stress adder a decade of repeated work stress and ***job loss***." |

| | | | |
|---|---|---|---|
| | | | • "Breakdown in family communication due to my failure to respond. ***Moved away from home,*** felt stranded and as if I had abandoned family. Partner's family high expectations due to masking."<br>• "***A lot of uncertainty, moving homes, schools, being separated from family for a long time,*** feeling unloved by family, feeling confused over low feelings"<br>• "wife broke up with me while i was recovering from surgery. couldn't afford to move out of my ex-wife's apartment after breaking up. then she moved in two girlfriends."<br>• " My housemates moved out to live with other friends during the lockdown and I was home alone and on furlough for the whole time"<br>• "***Moving to home*** to a place where me and my family knew nobody and no one liked us or wanted us there"<br>• "discovering a family member had cancer."<br>• "***My dad was diagnosed with terminal cancer when I was 19***, and my boss, who knew this, raped me"<br>• "Probably caused by ***childhood bereavement,*** which I had not begun to process."<br>• "***Unexplained breakup*** led to complete loss of purpose & worst case scenario reasoning strained relationships with everyone else. |

| | | | |
|---|---|---|---|
| | | | Couldn't speak to her so didn't want to speak to anyone else.”<br>• “***I watched my dad wither and die from leukaemia age 6-9***, I was parentifie and tortured by my sister when mum wasn't there (she's diagnosed bipolar in her20s, now ASD going for ADHDat40). Bad therapists”<br>• “Family struggling with mental health and living with separated parents”<br>• “Not fitting in, family rejection, ***homelessness and the death of one of my few friends.”***<br>• “***Losing my dad to breast cancer then finding out I had the BRCA2 gene. Getting married too quickly then divorced a year later***, plus feeling like a failure and an outsider my whole life.”<br>• “***Father went missing for several years***. Best friend got murdered age 34. ***Good friend died of cancer age 37. Gran died*** 2 months before BF murdered and I was ***6 months pregnant***. Rape coercive control”<br>• “childhood trauma which was never processed in part due my age (I lost my mum at 5years and my dad at 6 years)”<br>• “Partner splitting up with me because he didn't want to adopt (after telling me for years he did) then me believing my chance of ever adopting was over permanently because of mental health.” |

| | | | |
|---|---|---|---|
| | | | • "Family and romantic partners abuse or separation"<br>• "2020 was widowed after 46yrs, my husband was lost to dementia and I've been unable to resolve my grief in a healthier manner"<br>• "Systemic: only between ages 13 and 15, I was ***homeless*** (couch surfing), unable to get social service input b.c not abused enough. Highly intelligent (now PhD), unable to go to school b.c systemic factors" |
| | Trauma, abuse and/or assault<br><br>*This subtheme reflects contributions of traumatic experiences, abuse or assault to suicidal thoughts and feelings.* | Physical and/or sexual<br><br>*This sub-subtheme describes instances of physical and/or sexual abuse or assault.*<br>Back to the top | • "being overwhelmed! I had just left home, studying for A levels, very little money, no idea why I couldn't do the things other people did. socially isolated. ***childhood physical abuse***."<br>• "sexual abuse"<br>• "Sexual abuse"<br>• "My dad was diagnosed with terminal cancer when I was 19, and ***my boss, who knew this, raped me"***<br>• "***Sexual abuse until 26.*** Loss of two year old daughter in drowning accident in 2012. Both very important"<br>• "Father went missing for several years. Best friend got murdered age 34. Good friend died of cancer age 37. Gran died 2 months before BF murdered and I was 6 months pregnant. ***Rape coercive control"***<br>• "My Dad commited suicide when I was 16 and ***I was raped at 15*** and I didn't have |

| | | | |
|---|---|---|---|
| | | | adequate help to cope - I wasn't diagnosed with autism until I was almost 30 and trying to get help with my mental health”<br>• “***Sexual abuse from age 5***. Parents unaware. Parents therefore thought I was dramatic/hysterical. I didn’t understand my feelings or what the abuse was so thought I was indeed “misbehaving”. Confusion.”<br>• “I was ***sexually abused by my ex boyfriend*** and wasnt able to access support i was also being bullied by students and teachers at my school my autism and adhd wasnt diagnosed at the time i was spiralling”<br>• “At aged 7, I was bullied for bing posh and ***digitally raped by a bully.*** He gathered 20 boys to gut punch me often. Headmaster lied about me to my parents.Kept rape a secret from them.i cared for dad re”<br>• "***Sexual Assault*** earlier in my life”<br>• “***Sexual abuse (sister)*** but forgiven-  it was not innocent to me - ***only 8-9 at the time***.”<br>• “***Sexual abuse in early teenage years***”<br>• “I was ***raped as a child***. In addition, I was undiagnosed autistic and struggled hugely understanding why I felt so different to everyone else. I felt stupid, clumsy, awkward, couldn’t communicate.”<br>• “coming to terms with ***sexual abuse***, trying to cope with dysphoria and eating/self harm issues” |

| | | | |
|---|---|---|---|
| | | | • "***Sexual assault***, peers didn't believe me and abandoned me. Severe meltdown/flashback"<br>• "Being a victim of ***domestic violence***, very important."<br>• "When younger not understanding my undiagnosed condition and thinking I was mental because of my thoughts and behaviours. Later due to ***domestic violence***."<br>• "Sexually assaulted by middle school teacher"<br>• "Sexual abuse"<br>• "Pervasive sexual abuse as a disabled child."<br>• "Really abusive therapy. I cannot even call a ***domestic abuse hotline when I was raped*** because I could not mask during a panic attack" |
| | | Emotional<br><br>*This sub-subtheme describes instances of emotional abuse.*<br><br>Back to the top | • "Undiagnosed AuDHD pubescent child starting at elite private school on scholarship, no accommodations, with undiagnosed neurodivergent single mother on low income ***constantly criticising*** (& chronic pain)"<br>• "Very isolated childhood ***my mother was emotionally abusive*** and I learned to cope from being a very young child suppressing emotions I was very shy and unable to connect or build relationships" |

| | | | |
|---|---|---|---|
| | | | • "Consistent verbal, emotional and mental abuse from family."<br>• "Childhood trauma and emotional neglect."<br>• "In combination with the above factors, a step-father, who wanted me out of the way, persuaded me that I was a complete failure and that I would be better off dead"<br>• "Parental emotional abuse"<br>• "Narcissistic mother"<br>• "Emotional and mental abuse from parents"<br>• "Narcissistic abuse family and boyfriend"<br>• "Emotionally neglected as a child" |
| | | Polyvictimisation and other kinds of abuse or trauma<br><br>*Sometimes participants described multiple types of abuse or did not specify the nature of abuse. They also described traumatic experiences such as unexpected, traumatic losses, including by suicide.*<br><br>Back to the top | • "***My Dad commited suicide when I was 16*** and I was raped at 15 and I didn't have adequate help to cope - I wasn't diagnosed with autism until I was almost 30 and trying to get help with my mental health"<br>• "Physical violence in the family home (I was hit across the face or head approx. 1x month) and general parental disappointment, which was no longer an active factor once I lived independently."<br>• "Sexual abuse until 26. ***Loss of two year old daughter in drowning accident in 2012.*** Both very important"<br>• "My dog, was beaten v badly by several maniacs, in 1994.took me years to come to terms, with, the extremely vicious attack.my dog had to be put down." |

| | | | |
|---|---|---|---|
| | | | • "***Brother was murdered a few weeks ago***. Wife is disabled. Eldest son is autistic. Youngest son is in and out of hospital with ongoing health issues. Work 60 hours a week to make ends meet."<br>• "Domestic violence and psychological abuse"<br>• "Father went missing for several years. ***Best friend got murdered age 34.*** Good friend died of cancer age 37. Gran died 2 months before ***BF murdered*** and I was 6 months pregnant. Rape coercive control"<br>• "***Partner infidelity over decades suddenly coming to light- this was extremely traumatising after a shared trauma, not just "difficulty with romantic partner".*** SUDDEN collapse of mental &bodily health"<br>• "Psychosis, teenage hormones and ***trauma***"<br>• "***Domestic abuse,*** partner with mental health, exhaustion, self harm"<br>• "Family and romantic partners abuse or separation"<br>• "Being sectioned and admitted to a mental health ward."<br>• "I am writing in this just to say I couldn't think of anything outside of those categories, although quite a few of those questions intersect, for example my mental health issues stem from ***trauma***." |

| | | | |
|---|---|---|---|
| | | | • “2005 Following a ***friend's suicide.*** 2015 During episode of severe depression. 2020 In ***abusive relationship*** during lockdown”<br>• “Son died by suicide”<br>• “I have a ***close friend who has suicidal thoughts*** and was deemed a risk to others, although she was eventually able to recover before being sectioned. ***I was there on a few occasions.”***<br>• “Witness to relative’s suicide attempt”<br>• “***My father's suicide at age 47 unlocked these possibilities.*** Earlier I've never visualised suicides so vividly but with his death I started to have intrusive thoughts of various scenes.”<br>• “Abusive relationship which I didn’t understand was”<br>• “An inability to escape difficult circumstances and no prospects to be able to find an escape route. ***And domestic abuse”***<br>• “Having had abuse as a Child and also abusive husband.”<br>• “I was in a relationship where I was being abused. I am no longer in that situation anymore and even though that is the case I still feel trauma from that.”<br>• “forced transvestism, public spanking, solitary confinement at school.”<br>• “Most important was trauma from close relatives” |

| | | | |
|---|---|---|---|
| | | | • “Deliberate suicide baiting by an abuser”<br>• “Relationship based abuse / recovery after”<br>• “Chronic nightmares ***(trauma-related)*** meaning I never got a rest/break”<br>• “Medical trauma: I gave birth during the pandemic and was alone in labour for an hour, no visitors, haemorrhaged etc etc”<br>• “In particular, my severe OCD which I had to get intensive care for. Also, ***trauma from being exposed to something I should never have seen as child (child porn)”***<br>• “Trauma from being ostracised where I lived”<br>• “I didn't have difficulties accepting my gender or sexuality, but it was the crippling dysphoria mixed with the ***child abuse*** that was most influential.”<br>• “***Childhood abuse*** from my brother and isolation”<br>• “I watched my dad wither and die from leukaemia age 6-9***, I was parentifie and tortured by my sister when mum wasn't there*** (she's diagnosed bipolar in her20s, now ASD going for ADHDat40). Bad therapists”<br>• “Abuse whilst in care.”<br>• “***Childhood neglect and abuse.*** Medical neglect (having symptoms worsen while being told nothing is wrong and denying access to tests).” |

| | | | |
|---|---|---|---|
| | | | • "I grew up in an abusive environment and this is probably the biggest factor for me"<br>• "In hindsight, overwhelm and lack of social, emotional and physical support have factored into every single suicide attempt as the main issues on top of ***past unresolved trauma***."<br>• "Childhood abuse"<br>• "***Birth trauma*** and then reaching out to the NHS for support and getting NONE"<br>• "Childhood trauma and emotional neglect."<br>• "Unable to communicate needs due to abandonment"<br>• "These factors stack up. I don't remember being truly happy since very early childhood. I'm certain I have complex PTSD from ***trauma*** but I don't have a diagnosis or support with it."<br>• "I have severe OCD. I also had to leave school very early due to my ASD & physical health because the school didn't know how to accommodate me. ***One parent was abusing my other parent."***<br>• "35 years of narcissistic and domestic abuse."<br>• "***Family abuse***, religious hate, sexism, fatphobia, misogyny, lesbophobia, managers abuse"<br>• "Being trapped in an abusive relationship" |

| | | | |
|---|---|---|---|
| | | | • "Available support was not helpful, and ended up reinforcing ***traumatic***/stigmatising experiences."<br>• "Thoughts of losing someone very close to me. ***Abuse*** from autistic partner"<br>• "The effects of ***child abuse*** by birth family that used my autism as an excuse to scapegoate me leading to a serious dissociative disorder."<br>• "Having Trauma therapy for a suicidal ideation episode brought on by having not had help for ***55 year old abuse*** from Father that surfaced."<br>• "Not being able to fully understand my mental health symptoms and being scared by psychosis. Trouble explaining to proffessionals. Being dismissed/gaslit. ***Child abuse*** being minimised/overlooked."<br>• "Daily Parental abuse (emotional, psychological, financial, neglect, and rarely but occasionally physical) that lasted years from the age of 8 until 19."<br>• "Being sexually and emotionally abused"<br>• "Childhood physical and emotional abuse - a lot of what I was 'disciplined' for were manifestations of my autistic traits and I was denied access to support by my parents (e.g. counselling)."<br>• "Inability to control own finances due to fear of reprisal/financial coercion (from parents). Religious coercion (also from parents)." |

| | | | |
|---|---|---|---|
| | | | • "Wanting to move away ***from family who covered up that my brother sexually abused me.*** Not being able to my mum was appointee and I was treated like I couldn't think for myself. So much more…"<br>• "Alcoholic mother"<br>• "Environment - home and work, inability to escape from relentless triggers causing ***ongoing trauma***, never able to relax"<br>• "Inability to verbalise ***abuse*** that was happening. Not being aware it was important to tell someone or that it was wrong." |
| | Ongoing and/or chronic stressors<br><br>*Stressors, conceived of broadly as environmental demands requiring adaptation, featured frequently in participant comments, as reflected in this subtheme.* | Personal and everyday worries, "stresses and strains"<br><br>*This sub-subtheme reflects daily hassles and sources of strain including work, relationship conflicts, caring roles, everyday responsibilities and aversive environments. Note that participants often allude to the feelings they experience in relation to these stressors, which are explicitly coded in Theme 3.*<br><br>Back to the top | • "Brother was murdered a few weeks ago. ***Wife is disabled. Eldest son is autistic. Youngest son is in and out of hospital with ongoing health issues. Work 60 hours a week to make ends meet."***<br>• "Caring responsibilities"<br>• "Young carer for mother while also having a complicated relationship"<br>• "Parenthood"<br>• "Not wanting to move back with family. ***Negative effects of family environment.*** Wanting to live alone but unable to afford it."<br>• "***Having family and romantic relationships which were toxic***. Being alone to ***manage everything***. Having no-one to go to for advice or support. So |

| | | | |
|---|---|---|---|
| | | | lonely. Such an outsider. Not diagnosed then. My Dr 👍”<br>• “Unexplained breakup led to complete loss of purpose & worst case scenario reasoning ***strained relationships with everyone else.*** Couldn't speak to her so didn't want to speak to anyone else.”<br>• “Loneliness, isolation and contributing ***stress*** factors all played heavily”<br>• “Going against my values (breaking a strike), due to ***financial worry***.”<br>• “Not understanding why i was feeling what i was feeling. (Very important). ***Constent stress and overwhelm over many years. (Very important).*** Getting the wrong help. (Very important)”<br>• “Asking wife what I'd done wrong in the relationship. Her reply. If you don't know I'm not going to tell you”<br>• “Clashing with family members”<br>• “Family problems”<br>• “***Falling out with one of my children*** without understanding the reason. She wouldn’t speak to me.”<br>• “difficulty ***managing day to day life***, issues around food, medical and psychiatric abuse, gender dysphoria”<br>• “Accommodation problems (the surrounding is too noisy, too crowded, people around are too stressed, etc.)” |

| | | | |
|---|---|---|---|
| | | | • “Chronic fatigue, ***stress***, bullying at school and then work, no access to support for mental health and waiting years for autism assessment”<br>• “I had a full mental breakdown in 2018 following ***stress*** and recurrent of my PTSD. It led to a month of being sure I would kill myself. Previous situations were only ideations but at that time I was sure”<br>• “Stress of raising small children without support”<br>• “parents divorce, parents mental health, the depression genes, seasonal, ***stress***/overwhelmed. i constantly feel like im in a rush just  to keep up”<br>• “Specifically the DWP universal credit people bludgeoning their agenda and not caring to listen. ***Year of extra stress adder a decade of repeated work stress and job loss.”***<br>• “ADHD Medication Shortages. ***No control over stressful big changes esp. sudden change***. Uncertainty anxiety inc. waiting for important decisions to be made by others, inconsistent/unpredictable support”<br>• “Responsibilities”<br>• “existential crisis - unable to see any point in living and so why bother putting up with the ***stresses and strains***”<br>• “Feeling overwhelmed and unable to deal with all my ***responsibilities”*** |

| | | | |
|---|---|---|---|
| | | | • “Feeling like I could lose my job or place at uni being unable to keep up with the ***demands*** that others found easy”<br>• “Being on schedules that I could not control and that required me to be around large numbers of people, in busy environments, for more hours per week than I could handle.”<br>• “Interacting with people and the ***daily demands on me”***<br>• “Being tired of the ***weight day to day activities or chores such as cooking meals or doing the dishes***.”<br>• “***basic daily tasks***/ executive challenges”<br>• “Other people constantly living in the house”<br>• “When I have felt desperately lonely and unhappy and ***unable to cope with life and its demands***. When I’ve had problems with my adult children and thought them better off without me.”<br>• “Hospitalisation and ***reacclimation to everyday life***”<br>• “Dealing with difficult people in a work environment”<br>• “Noisy neighbours from hell”<br>• “***Juggling too many conflicting demands*** at one time”<br>• “Inability to cope with the ***day to day*** realities of being a ***parent, a*** |

| | | | |
|---|---|---|---|
| | | | ***wife/girlfriend, and an employee***, feeling deeply overwhelmed by ***day to day tasks***."<br>• "Not being able to cope with the ***demands of life*** and not wanting to be reliant on family to care for me"<br>• "It's not necessarily specific items like the ones you've listed. ***Life is hard in general, day after day of supposedly simple tasks*** compared to other people that are difficult to do is exhausting."<br>• "I find myself ***struggling to cope with the constant changes in life***. I can become so exhausted from ***trying to function every day*** and the prospect of being the way I am for the rest of my life."<br>• "***My current job position is a poor fit and often unsafe on site***, but I feel trapped there since it's first one I've had that hasn't forced me to stand all day or do cold calls."<br>• "I have a ***lot of trouble performing ordinary tasks, which makes daily life feel very difficult or impossible***. Also I am more stressed out by social contact than the lack of it."<br>• "Not understanding why ***I find life so hard*** (as I wasn't diagnosed until adulthood)"<br>• Finding society overwhelming sensory wise: too loud, bright, etc and having trouble keeping up with the ***fast pace of life."***<br>• "Not being able to cope with expectations, feeling overwhelmed and unable to keep up |

| | | | |
|---|---|---|---|
| | | | with ***daily life,*** sensitivity to rejection/criticism."" |
| | | "The state of the world": Extrapersonal events<br><br>*This sub-subtheme reflected the impact of stressors occurring at a global, national and/or local level, rather than necessarily affecting participants themselves.*<br><br>Back to the top | • "***Being affected by suffering and injustice around me*** and not being able to do anything about it."<br>• "Feeling angry and helpless about the state of the world."<br>• "***Climate anxiety*** and change has been a huge problem. Feeling utterly powerless in the face of colossal disaster has hugely impacted my outlook on life."<br>• "Lately, the following are additional factors along with the above options: Being stuck on benefits with demeaning health reviews, ***Loss of local nature (spaces and species), Climate change"***<br>• "Effects of COVID lockdown, politics and global affairs"<br>• " Despair at the state of the wider world"<br>• " Uncertainty relating to COVID lockdown and aftermath. Politics and global affairs."<br>• "Climate anxiety"<br>• "State of world/world events - not having a hopeful future because climate change, war etc."<br>• "Covid-19 Lockdown"<br>• "My concerns around the ***climate crisis*** and governments inability to do anything about it have significantly affected my mental health. This is now something I think about everyday. Also PMDD!" |

| | | | |
|---|---|---|---|
| | | | • "***So much suffering in this world with animals.*** Also, being lost in a neurotypical world BEING SWALLOWED up. No help or understanding either b4 diagnosis late in life!! Should b protected. Invisible"<br>• "Covid lockdown"<br>• "***Worried about economy*** and job prospects/unemployment in the future."<br>• "My housemates moved out to live with other friends during the ***lockdown*** and I was home alone and on furlough for the whole time"<br>• "Capitalism"<br>• "***Covid climate change*** pmdd"<br>• "lockdown isolation"<br>• "2005 Following a friend's suicide. 2015 During episode of severe depression. 2020 In abusive relationship ***during lockdown***" |
| | "Lack of understanding, lack of respect"<br><br>*This subtheme reflects instances where participants were misunderstood, invalidated, victimised, ostracised and/or discriminated against. These instances sometimes occurred in the context of insufficient awareness and resources, but other times reflected more malignant dismissal and disregard of autistic people* | "Lack of social, emotional and physical support"<br><br>*This sub-subtheme reflects instances where suicidal thoughts and feelings arose from the absence or inadequacy of support and accommodations.*<br><br>Back to the top | • "I was ***denied access to reasonable adjustments*** I needed to fly to see an elderly relative before she died."<br>• "Systemic: only between ages 13 and 15, I was homeless (couch surfing), ***unable to get social service input b.c not abused enough***. Highly intelligent (now PhD), unable to go to school b.c systemic facto"<br>• "Stress of raising small children without support"<br>• "I was sexually abused by my ex boyfriend and w***asnt able to access support*** i was also being bullied by students and teachers at |

| | | | |
|---|---|---|---|
| | *(whether or not they were known to be autistic)..* | | my school my autism and adhd wasnt diagnosed at the time i was spiralling”<br>• “***Support networks not available.*** Weather/time of year - March/April.”<br>• “Birth trauma and then ***reaching out to the NHS for support and getting NONE”***<br>• “Having family and romantic relationships which were toxic. Being alone to manage everything. ***Having no-one to go to for advice or support***. So lonely. Such an outsider. Not diagnosed then. My Dr 👍”<br>• “***no support at all with not understanding social interactions*** so i didnt know how to ask for any help or interact with any peer”<br>• “Undiagnosed AuDHD pubescent child starting at elite private school on scholarship, ***no accommodations,*** with undiagnosed neurodivergent single mother on low income constantly criticising (& chronic pain)”<br>• “Chronic fatigue, stress, bullying at school and then work, ***no access to support for mental health and waiting years for autism assessment***”<br>• “In hindsight, overwhelm and ***lack of social, emotional and physical support*** have factored into every single suicide attempt as the main issues on top of past unresolved trauma.”<br>• “These factors stack up. I don't remember being truly happy since very early |

| | | | |
|---|---|---|---|
| | | | childhood. I'm certain I have complex PTSD from trauma but I ***don't have a diagnosis or support with it***."<br>• " I have severe OCD. I also ***had to leave school very early due to my ASD & physical health because the school didn't know how to accommodate me***. One parent was abusing my other parent."<br>• "***Available support was not helpful***, and ended up reinforcing traumatic/stigmatising experiences."<br>• "Having Trauma therapy for a suicidal ideation episode brought on by ***having not had help*** for 55 year old abuse from Father that surfaced."<br>• "***Not enough mental health information to be easily found on which therapies will help,*** leaving me feeling hopeless, like a complete alien and that no one has ever had my mental health issues before."<br>• "My Dad commited suicide when I was 16 and I was raped at 15 and ***I didn't have adequate help to cope*** - I wasn't diagnosed with autism until I was almost 30 and trying to get help with my mental health"<br>• "ADHD Medication Shortages. No control over stressful big changes esp. sudden change. Uncertainty anxiety inc. waiting for important decisions to be made by others, ***inconsistent/unpredictable support"***<br>• "Becoming overwhelmed particularly emotionally often ***following contact with*** |

| | | | |
|---|---|---|---|
| | | | ***services (cmht, camhs, social services) when they do r understand my needs/how to communicate”***<br>• “Gender Dysphoria making existing difficult. Constantly being uncomfortable. ***Not being able to get help to be happy.”***<br>• “Not understanding why i was feeling what i was feeling. (Very important). Constent stress and overwhelm over many years. (Very important). ***Getting the wrong help. (Very important)”***<br>• “Thinking that getting an autism diagnosis would ***unlock help/support/understanding (it didn't).”***<br>• “***Complete lack of support and support being taken away from me for ‘not engaging’ but they don’t have any understanding of autism at all***. I am non verbal and they don’t take that into account”<br>• “Prolonged Autistic Burnout and ***lack of social/emotional support*** have played a huge part.”<br>• “Miscommunication, loneliness, unprocessed feelings, ***no help”***<br>• “***Lack of GP support***, was referred to a website and offered an anti-depressant.”<br>• “***Social services lack of funding to give me the care I need***. Not feeling safe in my own home or out in the community”<br>• “Being misunderstood and ***unsupported***” |

| | | | |
|---|---|---|---|
| | | | • "***Being dumped on my aging parents*** to be my carers when I was in my mid-30s & guilt of ruining their lives and their retirement"<br>• "I watched my dad wither and die from leukaemia age 6-9, I was parentifie and tortured by my sister when mum wasn't there (she's diagnosed bipolar in her20s, now ASD going for ADHDat40). ***Bad therapists***"<br>• "dealing with the benefit service since 2010, atos, universal credit, housing benefit etc"<br>• "Not being believed. Having to fight two tribunals against my employer and the DWP. Feeling alien. ***Feeling abandoned by health services."***<br>• "Omnipresent stigma requiring me to "mask". ***Can't take transport bcs of autism + health.*** Not being believed by doctors. ***Doctors unaware of autistic health***. Pple around me don't know what autism is"<br>• "Having incorrect medical advice pushed on me by family because my physio missed that I had more issues than they realized and the exercises I was given made the issues worse."<br>• "***A lack of understanding of autism within the NHS,*** and past refusal of access to an autism assessment."<br>• "Gender dysphoria, and ***having to wait years to get gender affirming care"*** |

| | | | |
|---|---|---|---|
| | | | • ***"NHS GIC waiting list/lack of informed consent system for HRT. being unable to convince my parents to consent to CAMHS referring me to the GIC.*** unexplained autism symptoms derailing my education." |
| | | "The immeasurable pain of thinking I'm safe and discovering I'm really unsafe"<br><br>*This sub-subtheme reflects experiences of antagonistic and uncaring systems and professionals. In contrast to absent/inadequate support in the previous subtheme, these are instances in which participants experienced invalidation or mistreatment by individuals in positions of responsibility and/or authority.*<br><br>Back to the top | • "Cannot progress to postgrad as 85% unemployment rate yet every Masters/PhD requires work experience / references. ***blanket HE gatekeeping is #1 limiting factor to our (autistic) success in employment."***<br>• "***Specifically the DWP universal credit people bludgeoning their agenda and not caring to listen***. Year of extra stress adder a decade of repeated work stress and job loss."<br>• "***Not being believed. Having to fight two tribunals against my employer and the DWP.*** Feeling alien. Feeling abandoned by health services."<br>• "***Asking for help and being denied it,*** feeling unable to cope with life, like the panic wouldnt ever stop."<br>• "I've never been able to cope with working full time so I am always having the stress of applying for benefits. ***The benefits system has been the thing that makes me most suicidal. It is cruel."***<br>• "Lately, the following are additional factors along with the above options: ***Being stuck on benefits with demeaning health*** |

| | | | |
|---|---|---|---|
| | | | ***reviews,*** Loss of local nature (spaces and species), climate change"<br>• "A lack of understanding of autism within the NHS, and ***past refusal of access to an autism assessment."***<br>• "Difficulties dealing with benefit system."<br>• "Feeling trapped in a situation with no exit- Incapacity assessment for DEP (ESA and PIP)."<br>• "A lot of sadness for many is the way you're looked upon by others, and then ***the government treat people disgusting by interrogation for benefits***. It has to stop for people with autism."<br>• "abusive neighbours, and ***mental health services telling me i was paranoid and delusional."***<br>• "***The way school system handled me over the years completely ruined every relationship*** they even apologised and tried letting me back"<br>• "I was sexually abused by my ex boyfriend and wasnt able to access support i was also ***being bullied by students and teachers at my school*** my autism and adhd wasnt diagnosed at the time i was spiralling"<br>• "Childhood neglect and abuse. ***Medical neglect (having symptoms worsen while being told nothing is wrong and denying access to tests)."*** |

| | | | |
|---|---|---|---|
| | | | • ***"People (NHS) not believing anything I said*** + very bad reaction to medication especially SSRIs and anti psychotics"<br>• "Not being able to fully understand my mental health symptoms and being scared by psychosis. ***Trouble explaining to proffessionals. Being dismissed/gaslit. Child abuse being minimised/overlooked."***<br>• "difficulty managing day to day life, issues around food, ***medical and psychiatric abuse***, gender dysphoria"<br>• "At aged 7, I was bullied for bing posh and digitally raped by a bully. He gathered 20 boys to gut punch me often. ***Headmaster lied about me to my parents. Kept rape a secret from them***. i cared for dad re"<br>• "***Really abusive therapy***. I cannot even call a domestic abuse hotline when I was raped because I could not mask during a panic attack"<br>• ***"***Bullied at work [redacted] for being autistic & ***threatened by line manager when I spoke to HR about it***, the immeasurable pain of thinking I'm safe and discovering I'm really unsafe."<br>• "Feeling I didn't "belong". Feeling people did not understand me, nor wanted to. ***Feeling those in positions of power were more interested in personal gain than helping, on both small and large scale***."<br>• "Omnipresent stigma requiring me to "mask". Can't take transport bcs of autism + |

| | | | |
|---|---|---|---|
| | | | health. ***Not being believed by doctors.*** Doctors unaware of autistic health. Pple around me don't know what autism is”<br>• “Medical gaslighting and bullying from health workers on more than one occasion. Was terrified and in pain. No one believed me. Had to pay for a diagnosis which was also dismissed by my GP”<br>• “ My environment was extremly toxic, I was bullied because I was different, ***even my teacher called me weird on a meeting with parents (mine were not there, she just was speaking about me to others)”***<br>• “Spending my whole life masking my autistic traits to survive but then ***not being believed when I ask for help later on after burn out***. ***Not getting help, being ignored, nowhere to go.”***<br>• “Feeling unheard/ignored/not taken seriously that I was suffering by family, friends, GPs”<br>• “***CAMHS was actively detrimental and dehumanising to me and the constant stress of their presence and control over my life*** with people who generally treated me like this made me want out”<br>• “NOT BEING BELIEVED BY NHS DR'S THAT I HAD AUTISM, ME/CFS AND PCOS. NOT BEING BELIEVED BY TEACHERS I WAS BEING BULLIED ABOUT MY FAITH AND LOOKS.” |

| | | | |
|---|---|---|---|
| | | | • “Civil servants, doctors, job centre staff etc. not being trauma sensitive and not informed about autism (or ANY mental health issues), resulting in malpractice, medical gaslighting, sanctions etc.”<br>• “Being misunderstood by everyone ***especially professionals especially teachers, doctors, consultants***”<br>• “Fear of losing loved ones. ***Mental health services not understanding my needs as an autistic individual”***<br>• “Counselling did not understand my thoughts and tried to talk me out of the thought. Saying I didn't mean it when I did! They asked me to change my GAD scores to reflect this.”<br>• “Despite my diagnosis was told to "Try Google" when I reached out to my GP for support for my condition”<br>• “Rejection by NHS mental health services due to BPD label - felt written off.”<br>• “Being academically intelligent but have a learning difficulty with Maths. ***No support with bullying at work. Had reprisals due to reporting it.”***<br>• “Not being understood by managers at work”<br>• “I had been in hospital for 1yr and ***told I was making up all my issues and being difficult*** with what I now recognise were meltdowns and sensory overwhelm.” |

| | | | |
|---|---|---|---|
| | | | • "medical personal misunderstandings that related to physiological injury but was perceived as mental health problem at that time. also, was accused of stuff that did not partake but was un-dx autist ." |
| | | "An uneducated and unempathetic society"<br><br>*This sub-subtheme reflects instances where participants were misunderstood, invalidated, treated unfairly, ostracised or victimised by peers, family, colleagues and society broadly.*<br><br>Back to the top | • "Neuro-Typicals"<br>• "Trauma from ***being ostracised*** where I lived"<br>• " Being disbelieved and misunderstood."<br>• "***Omnipresent stigma requiring me to "mask".*** Can't take transport bcs of autism + health. Not being believed by doctors. Doctors unaware of autistic health. ***Pple around me don't know what autism is"***<br>• "Having to mask, identity crisis, feeling like I always have to lie to get by - ***lack of understanding of autism and people constantly thinking it's the autistic person that has to change"***<br>• "I had trouble sleeping. ***People weren't listening to me when I tried to explain the issues I was having difficulty with."***<br>• ***"Not being taken seriously"***<br>• ***"Not being believed or listened to"***<br>• ***"Not being believed when I said I was struggling"***<br>• ***"Having my struggles invalidated/mocked or being gaslighted by abusive people."***<br>• "There have been 3 distinct episodes of not wanting to be here anymore for me. ***Age 8-*** |

| | | | |
|---|---|---|---|
| | | | ***badly bullied at school.*** 17-23, feeling like a failure socially. 26-30 ex separated me from my family"<br>• "***Never having the luxury of being properly understood. The constant negative assumptions*** made about my behaviour that were so hurtful and traumatising. Feeling outnumbered and unable to defend myself"<br>• ***"A lot of sadness for many is the way you're looked upon by others***, and then the government treat people disgusting by interrogation for benefits. It has to stop for people with autism."<br>• "Trying to fit into society's rules and expectations"<br>• "Feeling like I wasn't a real person. This is mental health but also ***people's prejudice."***<br>• "Had hard time growing up. ***Always made to feel like there's something wrong with you and that it's your fault you can't just get on in life."***<br>• ***"***The inability of others accepting I'm Autistic negative bias as I don't apparently look autistic- working environments injustices- people not accepting and believing my pain still today is difficult"<br>• "Not being bullied, but being excluded and ignored."<br>• "Social services lack of funding to give me the care I need. ***Not feeling safe in my own home or out in the community"*** |

| | | | |
|---|---|---|---|
| | | | • "Being ***misunderstood*** and unsupported"<br>• "I was ***used*** as a shield and I accepted that, to have somewhere live. I am still a tool today"<br>• "Parents overly questioning over my gender transition/ feelings and autism diagnosis/identity."<br>• "I needed a rest after a large family gathering and my family did not show any understanding for this (i.e. blamed me for being exhausted)."<br>• "***The lived experience of autism is to feel like you are being gaslit by society, constantly questioning your sense and perspectives***. The trauma of my brain injury combined with autism reinforces this."<br>• "***abusive neighbours***, and mental health services telling me i was paranoid and delusional."<br>• "Systemic: only between ages 13 and 15, I was homeless (couch surfing), unable to get social service input b.c not abused enough. ***Highly intelligent (now PhD), unable to go to school b.c systemic factors"***<br>• "I was sexually abused by my ex boyfriend and wasnt able to access support i was also ***being bullied by students*** and teachers at my school my autism and adhd wasnt diagnosed at the time i was spiralling" |

| | | | |
|---|---|---|---|
| | | | • "Family abuse, religious hate, sexism, fatphobia, misogyny, lesbophobia, managers abuse"<br>• "Available support was not helpful, and ended up reinforcing traumatic/***stigmatising experiences***."<br>• "At aged 7, I was ***bullied*** for bing posh and digitally raped by a bully. ***He gathered 20 boys to gut punch me often.*** Headmaster lied about me to my parents.Kept rape a secret from them.i cared for dad re"<br>• "Sexual assault, ***peers didn't believe me and abandoned me***. Severe meltdown/flashback"<br>• "Chronic fatigue, stress, ***bullying at school and then work***, no access to support for mental health and waiting years for autism assessment"<br>• "General fears related to being trans in the current political climate"<br>• "Not being understood by managers at work"<br>• "Hearing about discrimination against trans people and autistic people"<br>• "So much suffering in this world with animals. Also, ***being lost in a neurotypical world BEING SWALLOWED up.*** No help or understanding either b4 diagnosis late in life!! ***Should b protected. Invisible"***<br>• ***"Bullied at work*** [redacted] for being autistic & threatened by line manager when I spoke to HR about it, the immeasurable |

| | | | |
|---|---|---|---|
| | | | pain of thinking I'm safe and discovering I'm really unsafe.”<br>• “Helplessness: under constant sensory assault by several other degraded people, being 5 years ***set aside in a residence for the concentration of discarded by-products ("supported living")”***<br>• “Social anxiety: unable to communicate properly, social isolation, ***being made fun of***”<br>• “***Ostracisation and marginalisation*** (related mainly to being a single parent and having to home educate my son)”<br>• “***Discrimination*** lasts 6 months, the law should be changed to protect people under the equality act regarding unlawful practices at Universities and Businesses.”<br>• “Unfair dismissal”<br>• “Unable to keep a job and have good healthy relationships through ***lack of understanding, lack of respect and bullying gets me down all the time”***<br>• “Feeling of isolated from other autistic people, struggling with years of ***internalised ableism***. (Military)”<br>• “Always, and increasingly aware that for all my intelligence and creatve abilities, ***the world is not designed for people like me to prosper.”***<br>• “Isolation, I was ***bullied*** and as a result got homeschooled my days became |

| | | | |
|---|---|---|---|
| | | | monotonous and I couldn't and didn't have the opportunity to integrate with people”<br>• “Not knowing the cause of my difficulties: I was diagnosed just a few years ago, after I had become distressed about continually being ***used and then rejected by people***.”<br>• “I have a twin brother who is, as far as we know, neuro-typical. He is an over-acheiver and I ***have been held to his standards,*** despite having much more to overcome.”<br>• “***Pressure to fit in and be successful*** academically.”<br>• “***We have a society uneducated and unempathic towards autistic people***. Consistently masking everytime I go out in public is exhausting and makes you feel like you’re consistently playing a character.”<br>• “Constant emotional exhaustion from having to mask my autistic traits every waking moment and ***still being viewed negativity despite all my efforts.*** I just wanted a reprieve.”<br>• “***Trying to live up to other peoples expectations, who I am was not acceptable when I was growing up*** so I learned very quickly to mask to fit in.”<br>• “a pervasive feeling that no matter how hard I learn and try to make my way in life, I can never be fully accepted or successful on the terms that I feel that I should be (I'm aware of my arrogance...)” |

| | | | |
|---|---|---|---|
| | | | • "My workplace was conducting a neurodiversity campaign. I felt like I was being 'studied' or 'examined' it's almost like they knew I was ND before I did. I only found out last year"<br>• "Lack of respect from others"<br>• "discrimination on the basis of my autism"<br>• "Not being believed"<br>• ***"Not being able to live to society standards in many ways*** not being able to tolerate the world sensory"<br>• "Systemic ableism and being viewed as subhuman just for existing wrong"<br>• "Feeling unheard/ignored/not taken seriously that I was suffering by family, friends, GPs"<br>• "feeling ***misunderstood or like i wasnt being listened to***/barriers with how to express and explain what i was feeling to others/***my struggles being shrugged off as being nothing or no big deal etc"***<br>• "The times I have tried to end my life. I was tired of ***not being understood/being rejected,*** felt redundant, and I was physically exhausted. I wanted to switch off."<br>• "***Being falsely accused*** of intending to access obscene material via corporate internet access - the material was erotic but had already been broadcast on C4! Having suspected prostate cancer." |

| | | | |
|---|---|---|---|
| | | | • "I have tried  to kill myself because I didn't know why I was so different and ***everything I did upset people or made them hate me."***<br>• "Inability to form friendships and/ or romantic relationships, feeling that I could not be my true self/ would never be accepted for my true self, being ***misunderstood by others"***<br>• "***Not being understood*** and thinking differently from the vast majority of people."<br>• "I have experienced gender discrimination for being a woman in STEM."<br>• "my suicidal ideation is all historic but relates to growing up gay in the 80s in the middle of the Aids epidemic...at a time when hate crime was part of everyday life"<br>• "transphobia"<br>• "Being black in the UK and dealing with discrimination"<br>• "Sexism/Misogny"<br>• "I was treated for severe depression, developing into anxiety**.** I was on long term sick leave and had been subjected to ***unfair treatment***. My daughter had just been diagnosed ASD. I was but unaware of it"<br>• "Partly as a consequence of my autism diagnosis, the notion this suggested that there was something fundamentally wrong with me and that those ***people who had been awful to me*** were right somehow." |

| | | | |
|---|---|---|---|
| | | | • "***medical personal misunderstandings*** that related to physiological injury but was perceived as mental health problem at that time. also, ***was accused of stuff that did not partake*** but was un-dx autist ."<br>• "***Being groomed online*** at a young age (about 14) Undiagnosed neurodiverse and didn't realise what was happening and how I was treated."<br>• "***Being mis judged*** around having undiagnosed autism until it was recognised a year ago. Eg an autistic meltdown has been ***seen as aggressive, violent, attention seeking"***<br>• "People in my life who ***will not accept diagnosis*** if I did get one, causing me to mask more and be in a spiral of despair"<br>• "Undiagnosed autism, and misdiagnosis and ***mistreatment*** as a result." |
| Theme 3: Difficult thoughts and feelings<br><br>*This theme reflects the feelings and emotions that participants linked to suicidal thoughts and feelings. These were often mentioned in combination with* | "Uncertainty of the future"<br><br>*This subtheme reflects fears of death, loss, and of future events certain or possible.*<br><br>Back to the top | | • "***Fear of what would*** happen when I left school or if my parents die as ***I don't think I could live without the structure of education or without my parents"***<br>• "Being unable to deal with heat, smells, noises, lack of peace, lack of head space, and low energy. ***Awareness of consequences in old age of not being able to have children."***<br>• "Main problem being ***fear of losing job*** due to autistic traits as some skills are harder than others for this reason and can affect |

| | | | |
|---|---|---|---|
| *major and minor stressors (Theme 2), and/or challenges associated with neurodivergence or health conditions (Theme 1).* | | | how I can cope with my job and studying for professional exams"<br>• "***Feeling like I could lose my job or place at uni*** being unable to keep up with the demands that others found easy"<br>• "Chronic pain/declining health/***fear of getting ill"***<br>• "***Thoughts of losing someone very close to me.*** Abuse from autistic partner"<br>• "Worried about economy and ***job prospects/unemployment in the future."***<br>• ***"Fear of losing loved ones.*** Mental health services not understanding my needs as an autistic individual"<br>• "The impeding fear that myself and everyone around me will die one day."<br>• "Fear of the future (losing those I love, illness, loneliness)"<br>• "fear of death"<br>• "Fear of the Future"<br>• "Fear of the future"<br>• "Uncertainty of the future when thoughts that bad things could happen to us come up"<br>• "Fear of abandonment"<br>• "Existential dread"<br>• "Existential anxiety" |
| | "So lonely. Such an outsider."<br><br>*This subtheme covers feelings of disconnection, loneliness, feeling* | | • "***Inability to form friendships and/ or romantic relationships, feeling that I could not be my true self/ would never be*** |

| | | | |
|---|---|---|---|
| | *different to others and unaccepted/unloved.*<br><br>Back to the top | | ***accepted for my true self***, being misunderstood by others"<br>• "Feeling like I don't exist/invisible"<br>• "Having no friends just acquaintances"<br>• "I have tried  to kill myself because ***I didn't know why I was so different*** and everything I did upset people or made them hate me."<br>• "never feeling like I belonged anywhere that would be accepting of me as I am"<br>• "Not enough mental health information to be easily found on which therapies will help, leaving me feeling hopeless, ***like a complete alien and that no one has ever had my mental health issues before***."<br>• "Not being understood and ***thinking differently from the vast majority of people."***<br>• "***Feeling like an alien in a human world and feeling so different that the world seemed a crazy place to me.*** Also that I seemed to hurt people when I was only trying to be good.  It felt better to go"<br>• "Feeling like no one is ever going to understand me."<br>• "being overwhelmed! I had just left home, studying for A levels, very little money, ***no idea why I couldn't do the things other people did. socially isolated.*** childhood physical abuse."<br>• "Nobody cares" |

| | | | |
|---|---|---|---|
| | | | • “***Feeling I didn't "belong". Feeling people did not understand me, nor wanted to.*** Feeling those in positions of power were more interested in personal gain than helping, on both small and large scale.”<br>• “Also, Experiencing/ having Acute sensory afflictions brings with it challenges beyond anyone's understanding, causing a ***massive disconnect*** within society, but not nature. ***I feel I'm from another world”***<br>• “Feeling abandoned”<br>• “Difficulty fitting into society, particularly in romance and work as well as my peer group at school.”<br>• “Feeling unwanted and not loved”<br>• “Worry of People not liking me and not having friends”<br>• “Unable to live totally alone”<br>• "Relating to other people - Very important"<br>• “***I've never fitted in anywhere. I'm only ever seen as useful and have never been loved by an adult human since my nan died.*** I can't access any help and I don't know why nobody cares.”<br>• “A lot of uncertainty, moving homes, schools, being separated from family for a long time, ***feeling unloved by family***, feeling confused over low feelings”<br>• “***Not fitting in, family rejection***, homelessness and the death of one of my few friends.” |

| | | | |
|---|---|---|---|
| | | | • "***Isolation***, I was bullied and as a result got homeschooled ***my days became monotonous and I couldn't and didn't have the opportunity to integrate with people"***<br>• "Difficulty fitting into society, particularly in romance and work as well as my peer group at school."<br>• "Losing my dad to breast cancer then finding out I had the BRCA2 gene. Getting married too quickly then divorced a year later, plus feeling like a failure and ***an outsider my whole life."***<br>• "no support at all with not understanding social interactions so ***i didnt know how to ask for any help or interact with any peer"***<br>• "Inability to properly socially connect with others and the effort required to do so, and still not getting it right often enough to have friends very often."<br>• "***Feeling like no one understands me***. Unable to have a grip on reality. Just feeling very tired/overestimated. ***Felt that I wasn't built for this world/I don't fit in it."***<br>• "Feeling of ***isolated from other autistic people***, struggling with years of internalised ableism. (Military)"<br>• ***"***Leaving my religion (Islam) when I was 36 years of age and ***being shunned and rejected by family and friends - a sense of loss of identity, purpose and belonging.*** Also great disappointment & regrets." |

| | | | |
|---|---|---|---|
| | | | • "Difficulties in obtaining a support network"<br>• "***Loneliness, isolation*** and contributing ***stress*** factors all played heavily"<br>• "***I remember each time feeling very alone and as though the world was an alien world I could not understand or navigate*** and I was only being a burden to others or getting myself into pickles"<br>• "Not being believed. Having to fight two tribunals against my employer and the DWP. ***Feeling alien.*** Feeling abandoned by health services."<br>• "Not being able to be alone."<br>• "Feeling inadequate as a person, like I don't matter, and not understanding why - confusion about ***why I don't fit in*** and feeling 'less than' other people"<br>• "General struggles especially ***socially***, and dealing with mental health . I have drug resistant depression which I have been diagnosed with since 14."<br>• "There have been 3 distinct episodes of not wanting to be here anymore for me. Age 8- badly bullied at school. ***17-23, feeling like a failure socially. 26-30 ex separated me from my family"***<br>• ***"Not knowing what I did wrong or how to fix it. Having no one I felt I could talk to,*** feeling the Samaritans etc would laugh at me." |

| | | | |
|---|---|---|---|
| | | | • "Miscommunication, ***loneliness***, unprocessed feelings, no help"<br>• "Feeling as though I am generally ***a failure in social settings***. Replaying conversations obsessively to try and understand ***why I don't fit in."***<br>• "A common theme was ***feeling disconnected*** and incompatible with the expectations of being alive as a human being."<br>• "No clear path or place for me in the world"<br>• "Just a combination of everything. ***Feeling as though I don't fit into this world*** but knowing I have no choice. I can't continue to do what's expected. ***Feeling that I can't talk to anybody.*** Hopelessness"<br>• "I felt like I ***couldn't connect with other people. I felt like people could tell that there was something 'wrong' with me.*** I was also really frustrated about failing at things others found easy"<br>• "Feeling not good enough***. Feeling differently to other people. Not considered "normal""***<br>• "I have no goal and I am useless. I can not do anything. I achieved nothing at school, can't work or get married, ***I have no friends***. I have behavioural issues."<br>• "Social anxiety: unable to communicate properly, ***social isolation,*** being made fun of" |

| | | | |
|---|---|---|---|
| | | | • "Childhood abuse from my brother and ***isolation***"<br>• "***Very isolated childhood*** my mother was emotionally abusive and I learned to cope from being a very young child suppressing emotions I was ***very shy and unable to connect or build relationships"***<br>• "Social aspect of school and feeling like an ***outsider / not being 'normal'*** in comparison to peers."<br>• ***"***I was raped as a child. In addition, I was undiagnosed autistic and ***struggled hugely understanding why I felt so different to everyone else.*** I felt stupid, clumsy, awkward, couldn't communicate."<br>• "Comparison - ***feeling like I was finding life much more difficult than others*** and the shame that comes from that. Feeling like I wasn't "allowed" to struggle because I'm intelligent."<br>• "When I have felt ***desperately lonely*** and unhappy and unable to cope with life and its demands. When I've had problems with my adult children and thought them better off without me."<br>• "I have only in the last 12 months come to realise I am autistic (I'm high masking/low needs) and ***did not understand why I was not normal***, nor how to look after myself as an autistic." |

| | | | |
|---|---|---|---|
| | | | • “Having family and romantic relationships which were toxic. ***Being alone to manage everything. Having no-one to go to for advice or support. So lonely. Such an outsider***. Not diagnosed then. My Dr 👍”<br>• “Managing ADHD alongside autism (while not knowing I had the condition until about 22). The feeling ***like I'm behind my peers*** in terms of mental”<br>• “I believe drugs enhanced my feelings of ***loneliness and hopelessness*** as well as suicidal thoughts.”<br>• “isolation”<br>• “Strong feeling that I don't belong in this world”<br>• “I ***didn't fit in at school*** and I was in an immense amount of physical pain due to growing too quickly and not being given the proper diagnosis/meds by doctors as they just thought it was growing pains”<br>• “Not knowing I was autistic and therefore not even knowing what is was that needed to be accommodated. ***Feeling broken and like a failure for not being able to do or be like other around me”***<br>• ***“I knew I was different, you liked me or didn't.*** I had a lovely husband and ***my differences really have resurfaced.*** Diagnosed autistic on my 61st Birthday” |

| | | | |
|---|---|---|---|
| | | | • ***"Thinking I wasn't made right/ too different to others*** due to not knowing I am autistic"<br>• "I had not recived an autism diagnoses until I was an adult, ***I felt like id never fit anywhere, I didnt understand why I found things difficult."***<br>• "Not having an autism diagnosis and ***not knowing why I was different and why I couldn't fit in"***<br>• "Not knowing why things were wrong. ***Wanting to be/feel normal. Not feeling able to be myself."***<br>• "I was undiagnosed until my late 50's- I ***always knew I was different/had issues*** but never understood what they were"<br>• "not understanding or realising that I'm autistic, but ***trying to be as "normal" as possible"*** |
| | "Too defective to live"<br><br>*This subtheme reflects feelings of worthlessness (or being worth less than other people), self-hatred, and/or burdensomeness.*<br><br>Back to the top | | • ***"hating myself, feeling like a burden, believing it would be better for everyone***, feeling trapped in my own thoughts"<br>• "An all pervasive feeling that I was ***wrong, bad, and a failure at life*** and a deep crushing sense of ***shame*** around who I was."<br>• "Losing my dad to breast cancer then finding out I had the BRCA2 gene.  Getting married too quickly then divorced a year |

| | | | |
|---|---|---|---|
| | | | later, plus ***feeling like a failure*** and an outsider my whole life."<br>• "Comparison - feeling like I was finding life much more difficult than others and the ***shame that comes from that***. Feeling like I wasn't "allowed" to struggle because I'm intelligent."<br>• "Helplessness: under constant sensory assault by several other ***degraded people***, being 5 years set aside in a residence for the concentration of ***discarded*** by-products ("supported living")"<br>• "Feeling like an alien in a human world and feeling so different that the world seemed a crazy place to me. Also that ***I seemed to hurt people when I was only trying to be good. It felt better to go"***<br>• "Feeling ***inadequate as a person, like I don't matter,*** and not understanding why - confusion about why I don't fit in and ***feeling 'less than' other people"***<br>• "Felt ***too defective to live***."<br>• "overwhelming feeling of ***self-hatred"***<br>• ***"Feeling not good enough***. Feeling differently to other people. Not considered "normal""<br>• "A common theme was feeling disconnected and ***incompatible with the expectations of being alive as a human being***."<br>• "Having lots of intense emotions. Having Big emotions. Needing things to stop. |

| | | | |
|---|---|---|---|
| | | | Needing a brake from me. Injustice's. ***Feeling unfixable."***<br>• "When I have felt desperately lonely and unhappy and unable to cope with life and its demands. ***When I've had problems with my adult children and thought them better off without me."***<br>• "Not being able to cope with the demands of life and ***not wanting to be reliant on family to care for me***"<br>• "Being ***dumped on*** my aging parents to be my carers when I was in my mid-30s & ***guilt of ruining their lives and their retirement***"<br>• "I remember each time feeling very alone and as though the world was an alien world I could not understand or navigate and ***I was only being a burden to others or getting myself into pickles"***<br>• "I felt like I couldn't connect with other people. I felt like people could tell that there was ***something 'wrong' with me.*** I was also really frustrated about ***failing*** at things others found easy"<br>• "I have no goal and ***I am useless. I can not do anything***. ***I achieved nothing at school,*** can't work or get married, I have no friends. I have behavioural issues."<br>• "The times I have tried to end my life. I was tired of not being understood/being rejected, ***felt redundant***, and I was |

| | | | |
|---|---|---|---|
| | | | physically exhausted. I wanted to switch off.”<br>• “Addiction to self harm, loosing my independence due to debilitating OCD for a time ***and needing to depend on others”***<br>• “Not realising I was autistic and that's why I was struggling to cope, ***feeling I was stupid and a failure.*** Formal diagnosis age 56.”<br>• “Undiagnosed PTSD - treatments for other disorders not helping or making me feel worse (due to triggers) and ***feeling like a failure and its my fault***”<br>• “Depression was the dominant factor. ***Feeling that everyone would be better off if I wasn't around.”***<br>• “Not knowing I was autistic and therefore not even knowing what is was that needed to be accommodated. ***Feeling broken and like a failure*** for not being able to do or be like other around me”<br>• “Partly as a consequence of my autism diagnosis, the notion this suggested that ***there was something fundamentally wrong with me and that those people who had been awful to me were right*** somehow.”<br>• “I just always felt so ***worthless and disgusting and weird***. I ***hated myself*** as a teen & thought I was ***evil*** for having meltdowns and getting mad at my loved ones.” |

| | | | |
|---|---|---|---|
| | | | • "Feeling like a burden to my spouse because I cannot work much"<br>• "Being so sensitive to the energy around me I can feel every mood in the room. Especially negative and it is a lot to process when I feel like the ***negative moods people are in is because of me somehow***"<br>• "Being undiagnosed and so not understanding ***why I didn't measure up.*** Setting too high a standard for my. Others doing that. Trying to be what everyone one wanted. Struggling financially despite doing b"<br>• "Existential nihilism, antinatalism (not anti-natalism), misanthropy, ***dislike of self***, dislike of being alive. All ranked as Very Important"<br>• "Not sure if this is covered under 'hopelessness' above but a ***complete feeling of worthlessnes*** - very much feeling 'what's the point?'"<br>• "Had hard time growing up. Always made to feel like there's s***omething wrong with you and that it's your fault*** you can't just get on in life."<br>• "Feeling ***shame and guilt and overwhelmed with self hate and hopelessness*** that I can never have good stable relationships with friends or partners and ***feeling like I hurt everyone I love***"<br>• "Feeling unable to be independent"<br>• "Unable to live totally alone" |

| | | | |
|---|---|---|---|
| | | | • "Not being able to be alone."<br>• "The ways in which my death would benefit my child."<br>• "Low self esteem"<br>• "***Self hatred*** and feeling overwhelmed"<br>• ***"Feeling like a burden/*** not being able to cope with things that others seemed to find easy"<br>• "Brought up with a sense of ***shame***"<br>• "Feeling like I am a ***bother***"<br>• "Feeling a ***burden to others***"<br>• "I felt like I was disappointing everyone I cared about - very important. Nothing could help me- moderately important"<br>• "***Feeling like everyone around me would be better off without me*** in their lives (my parents, brother, friends and children, all on seperate occasions)"<br>• "Feeling ***like a burden*** to those around me."<br>• "Just being able to accept myself" |
| | "Feeling like my life isn't going anywhere"<br><br>*This subtheme describes feelings of existential meaningless; where participants felt they lacked purpose, meaning, and reasons for living that could offset the difficulty of living.* | | • ***"Feeling stuck/feeling like my life isn't going anywhere"***<br>• "Unexplained breakup led to ***complete loss of purpose*** & worst case scenario reasoning strained relationships with everyone else. Couldn't speak to her so didn't want to speak to anyone else."<br>• "***I have no goal*** and I am useless. I can not do anything. I achieved nothing at school, |

| | | | |
|---|---|---|---|
| | Back to the top | | can't work or get married, I have no friends. I have behavioural issues."<br>• "Not knowing I was autistic/had autism. ***No interest in future.*** not make sense of emotion's, identify or explain. Made sense via a positive / negative boundary of feeling sick vs not feeling sick."<br>• "Leaving my religion (Islam) when I was 36 years of age and being shunned and rejected by family and friends - a sense of ***loss of*** identity, ***purpose*** and belonging. Also great disappointment & regrets."<br>• "***existential crisis - unable to see any point in living*** and so why bother putting up with the stresses and strains"<br>• "***Existential nihilism***, antinatalism (not anti-natalism), misanthropy, dislike of self, ***dislike of being alive***. All ranked as Very Important"<br>• ***"Life is difficult and not very enjoyable"***<br>• "The prospect of having to live my life barely scraping by, with no energy or will left to enjoy anything, ***seems so completely unappealing***, and I can't see that it can be any different."<br>• "***No clear path*** or place for me in the world"<br>• "Not sure if this is covered under 'hopelessness' above but a complete feeling of worthlessnes - ***very much feeling 'what's the point?'"*** |

| | | | |
|---|---|---|---|
| | | | • "a sense that ***no longer existing might be a rational solution to the problems inherent in existing***"<br>• "Felt like ***nothing more would happen with my life***."<br>• "***Lack of a sense of purpose*** and no hope for the future" |
| | "Deeply overwhelmed" with "no apparent escape"<br><br>*In this subtheme, participants describe feeling unable to cope, feeling 'overwhelm' and exhaustion. These quotes appear to reflect defeat and entrapment; entrapment emerges in both internal ('trapped in my thoughts') and external forms ('* ***trapped in a situation with no exit***- Incapacity assessment for DEP [ESA and PIP]."). *Sometimes, participants reflect hopelessness in relation to this.*<br><br>Back to the top | | • "***Overwhelm*** -- not exactly the same thing as feeling hopeless or worthless, but facing too many stressors in too short/pressing a timeframe with no apparent escape or break other than death"<br>• "Overwhelm"<br>• " I think I felt very ***overwhelmed and trapped***"<br>• "parents divorce, parents mental health, the depression genes, seasonal, stress/***overwhelmed. i constantly feel like im in a rush just to keep up"***<br>• "***Inability to cope*** with the day to day realities of being a parent, a wife/girlfriend, and an employee, feeling ***deeply overwhelmed*** by day to day tasks."<br>• "Not realising I was autistic and that's why I was ***struggling to cope***, feeling I was stupid and a failure. Formal diagnosis age 56."<br>• "***Not being able to cope*** with the demands of life and not wanting to be reliant on family to care for me" |

| | | | |
|---|---|---|---|
| | | | • "Feeling like a burden/ ***not being able to cope*** with things that others seemed to find easy"<br>• "Being ***so confused and overwhelmed at just existing in general that I couldn't cope***"<br>• "Domestic abuse, partner with mental health, ***exhaustion***, self harm"<br>• "Constant emotional exhaustion from having to mask my autistic traits every waking moment and still being viewed negativity despite all my efforts. ***I just wanted a reprieve."***<br>• "I think it links back to hopelessness but every attempt was definitely motivated by a state of frustration and overwhelm, feeling like I can never be helped and I'm ***doomed to struggle my whole life."***<br>• "I was ***used*** as a shield and ***I accepted that***, to have somewhere live. I am still a tool today"<br>• "***Being disempowered***, loss of agency and autonomy - particularly in ways others do not - are key underlying to all mentioned above"<br>• "Chronic exhaustion"<br>• "Exhaustion"<br>• "Feeling unable to be independent"<br>• "Feeling like I could lose my job or place at uni ***being unable to keep up*** with the demands that others found easy" |

| | | | |
|---|---|---|---|
| | | | • "People in my life who will not accept diagnosis if I did get one, causing me to mask more and ***be in a spiral of despair"***<br>• ***"Feeling of unable to cope and overwhelm*** and feeling unwell with fatigue"<br>• "Feeling like no one understands me. ***Unable to have a grip on reality. Just feeling very tired/overestimated***. Felt that I wasn't built for this world/I don't fit in it."<br>• "Self hatred and ***feeling overwhelmed"***<br>• "Being on schedules that I ***could not control*** and that required me to be around large numbers of people, in busy environments, ***for more hours per week than I could handle."***<br>• "***Helplessness***: under constant sensory assault by several other degraded people, being 5 years set aside in a residence for the concentration of discarded by-products ("supported living")"<br>• "Just being ***totally overwhelmed*** and not being able to understand my situation or why I was struggling. My living situation was chaotic and ***I had no safe space***"<br>• "It's not necessarily specific items like the ones you've listed. Life is hard in general, day after day of supposedly simple tasks compared to other people that are difficult to do is ***exhausting***."<br>• "People were trying to make me go to a special school but ***i really didn't want or need to***." |

| | | | |
|---|---|---|---|
| | | | • "Addiction to self harm, ***loosing my independence*** due to debilitating OCD for a time ***and needing to depend on others"***<br>• "Feeling ***trapped in a situation with no exit***- Incapacity assessment for DEP (ESA and PIP)."<br>• "When I have felt desperately lonely and unhappy and ***unable to cope with life and its demands***. When I've had problems with my adult children and thought them better off without me."<br>• "Spending my whole life masking my autistic traits to survive but then not being believed when I ask for help later on after burn out. Not getting help, being ignored, ***nowhere to go."***<br>• ***"***Never having the luxury of being properly understood. The constant negative assumptions made about my behaviour that were so hurtful and traumatising. ***Feeling outnumbered and unable to defend myself"***<br>• "CAMHS was actively detrimental and dehumanising to me and the constant stress of their presence and ***control over my life*** with people who generally treated me like this ***made me want out"***<br>• ***"An inability to escape difficult circumstances and no prospects to be able to find an escape route.*** And domestic abuse" |

| | | | |
|---|---|---|---|
| | | | • ***"Feeling overwhelmed*** and ***unable to deal with*** all my responsibilities"<br>• ***" just complete overwhelm from life.*** Especially after an adhd high"<br>• ***"***Burnout / ***exhaustion***, alcoholism, forced drug use"<br>• "Having lots of intense emotions. Having Big emotions. ***Needing things to stop. Needing a brake from me.*** Injustice's. Feeling unfixable."<br>• "Being ***tired*** of the weight day to day activities or chores such as cooking meals or doing the dishes."<br>• "I find myself struggling to cope with the constant changes in life. I can become so ***exhausted*** from trying to function every day and ***the prospect of being the way I am for the rest of my life."***<br>• "I have a lot of trouble performing ordinary tasks, which makes ***daily life feel very difficult or impossible***. Also I am more stressed out by social contact than the lack of it."<br>• "***being overwhelmed***! I had just left home, studying for A levels, very little money, no idea why I couldn't do the things other people did. socially isolated. childhood physical abuse."<br>• "In hindsight, ***overwhelm*** and lack of social, emotional and physical support have factored into every single suicide attempt as |

| | | | |
|---|---|---|---|
| | | | the main issues on top of past unresolved trauma."<br>• " Being ***trapped*** in an abusive relationship"<br>• "***Wanting to move away from family who covered up that my brother sexually abused me. Not being able to my mum was appointee and I was treated like I couldn't think for myself.*** So much more…"<br>• "ADHD Medication Shortages. No control over stressful big changes esp. sudden change. ***Uncertainty anxiety inc. waiting for important decisions to be made by others, inconsistent/unpredictable support"***<br>• "Being affected by suffering and injustice around me and ***not being able to do anything about it."***<br>• "Feeling angry and ***helpless*** about the state of the world."<br>• "Climate anxiety and change has been a huge problem. ***Feeling utterly powerless*** in the face of colossal disaster has hugely impacted my outlook on life."<br>• "Burnout and ***feeling unable to deal with the complicated world I live in."***<br>• "A large factor was the sense of responsibility I felt for all my actions. I felt ***exhausted*** by having to take responsibility for my own actions, and I wanted to ***get rid of*** that sense of responsibility." |

| | | | |
|---|---|---|---|
| | | | • "Asking for help and being denied it, ***feeling unable to cope with life, like the panic wouldnt ever stop."***<br>• "***Not being able to cope with expectations, feeling overwhelmed and unable to keep up with daily life***, sensitivity to rejection/criticism."<br>• "Environment - home and work, ***inability to escape from relentless triggers causing ongoing trauma***, never able to relax"<br>• "Not wanting to move back with family. Negative effects of family environment. ***Wanting to live alone but unable to afford it."***<br>• "My current job position is a poor fit and often unsafe on site, but I feel ***trapped*** there since it's first one I've had that hasn't forced me to stand all day or do cold calls."<br>• "no physical space to ***escape*** sensory overload: perfume and laundry fragrances of neighbours pushing through closed doors, traffic noise, constant seizure triggering, no sleep, ***trapped inside***."<br>• "Just a combination of everything. Feeling as though I don't fit into this world but knowing ***I have no choice. I can't continue to do what's expected.*** Feeling that I can't talk to anybody. Hopelessness***"***<br>• "The times I have tried to end my life. I was tired of not being understood/being rejected, felt redundant, and ***I was*** |

| | | | |
|---|---|---|---|
| | | | ***physically exhausted. I wanted to switch off."***<br>• "***Feeling trapped*** and being a known figure in the community - minister's wife. Undiagnosed autism. Feeling like the GP couldn't help - being passed from one professional to another."<br>• "***Having no actionable plan to escape*** relentless overwhelm of sensory and social experience which would also ***ensure ongoing relief***. As a teenager not knowing how to unmask - ***suicide only option."***<br>• "Wanting the feeling of constant burn out ***to just stop***."<br>• "Having problems that I can see the solution to but ***can't influence that decision to be made.*** Spiraling thinking chaotic thoughts."<br>• "hating myself, feeling like a burden, believing it would be better for everyone, ***feeling trapped in my own thoughts"***<br>• "Being noticeably much more intelligent than the other humans but ***not being able to use it to achieve anything, and not being able to stop*** smoking cannabis (addicted for 20 years)"<br>• "That I live in this country I hate it and I am not from here but I ***can't escape*** because of my partners job."<br>• "***Inability to control own finances*** due to fear of reprisal/financial coercion (from |

| | | | |
|---|---|---|---|
| | | | parents). Religious coercion (also from parents).”<br>• “Feeling ***overwhelmed*** in general”<br>• “***Not being able to cope with expectations, feeling overwhelmed and unable to keep up*** with daily life, sensitivity to rejection/criticism.”<br>• “The points where I have attempted suicide were usually when big changes going on ***and I just couldn't cope***. I still get very low thoughts and consider it but don't action, ***this is when too overwhelmed”***<br>• “Having run out of energy or hope for change” |
| Theme 3: Difficult thoughts and feelings<br><br>*There were some additional thoughts and emotions which did not occur with sufficient frequency to justify a separate subtheme. These include feelings of guilt, depersonalisation, and hopelessness; the latter emerged frequently in relation to the mental states* | | | • “***Guilt.”***<br>• “***Guilt*** and wanting to end my life so the people I've hurt felt rest-assured that I am not in this world any longer to hurt them or anybody else. I still feel this way.”<br>• “Depression kept coming back every time I thought I'd solved it and moved on. It made me feel ***hopeless about the future.”***<br>• “Feeling like ***things will never get better/easier for me.”***<br>• “Breakdown in family communication due to my failure to respond. Moved away from home, felt stranded and ***as if I had abandoned family***. Partner’s family high expectations due to masking.” |

| | | | |
|---|---|---|---|
| *reflected in other themes, but less frequently by itself.* | | | • ***"Perceived loss of my Christian faith and related anxiety.*** Leaving school (was in a gap year job) contributed."<br>• ***"***Leaving my religion (Islam) when I was 36 years of age and being shunned and rejected by family and friends - a sense of ***loss of identity***, purpose and belonging. Also ***great disappointment & regrets."***<br>• "Life not really having time to pause / stop and ***feeling left behind*** due to burnout***"***<br>• ***"Symptoms of hopelessness*** got worse as I got older and I kept going through breakdowns. That was before my diagnosis at the age of 51"<br>• "Health decline over many years making life more and more difficult to endure. Pain and suffering is constant. There is continued torment in every moment of every day. ***Loss of 'self' also."***<br>• "The complexity of having an autistic sibling with higher needs. I'm pressured to be silent about how they mistreated me as a kid. I feel ***guilty*** for being unable to look after them, unlike my sister."<br>• "***Feeling like I wasn't a real person.*** This is mental health but also people's prejudice."<br>• "Feeling ***like I wasn't a person or real"***<br>• "***Feeling numb*** and exhausted by living" |
| Theme 4: "I was lost" | | | • "Thinking everything would get better after school/bullying and I would be a new |

| | | | |
|---|---|---|---|
| *This theme reflects instances where late diagnosis was explicitly or implicitly linked to suicidal thoughts and feelings. Late diagnosis frequently intruded into other themes: it was, for instance, frequently alluded to in the context of victimisation, stress and trauma (Theme 2); to feelings of difference, helplessness, overwhelm and worthlessness (Theme 3); and to not understanding the features of their neurodivergence (Theme 1). In relation to late diagnosis, participants often mentioned confusion as a dominant experience.* | | | happy and confident person once I went to university. But ***I was still having problems and didn't understand why."***<br>• " Not having any 'trigger' or cause for my depression ***until being late diagnosed with autism and, sometime after, that that was the primary cause***."<br>• ***" Not understanding my neurodivergence,*** not recognizing that my mental health was in crisis until I hit the bottom"<br>• ***"***being overwhelmed! I had just left home, studying for A levels, very little money, ***no idea why I couldn't do the things other people did.*** socially isolated. childhood physical abuse."<br>• "***Not knowing I was autistic/had autism.*** No interest in future. not make sense of emotion's, identify or explain. Made sense via a positive / negative boundary of feeling sick vs not feeling sick."<br>• "***Before ASD diagnosis*** the ever increasing doses of mental health medication - horrendous side effects and worse withdrawal. ***Not acknowledged by relevant medical professionals who just kept prescribing."***<br>• "I had been in hospital for 1yr and told I was making up all my issues and being difficult ***with what I now recognise were meltdowns and sensory overwhelm***."<br>• "Feeling inadequate as a person, like I don't matter, and ***not understanding why*** - |

| | | | |
|---|---|---|---|
| [Back to the top](#) | | | ***confusion about why I don't fit in*** and feeling 'less than' other people"<br>• "***Being diagnosed at 63yrs*** as I have been misunderstood by GP's as they made unvalid assumptions about me so didn't get treated well with medical conditions such as skin problems & hormonal issues"<br>• "Not being diagnosed until my last year of secondary school."<br>• "I didn't know I am Autistic"<br>• "Not even considering that I might be autistic."<br>• "***Being undiagnosed autistic with adhd*** and dealing with feelings of confusion and distress"<br>• "***Thinking that getting an autism diagnosis*** would unlock help/support/understanding (it didn't)."<br>• "Just being totally overwhelmed and ***not being able to understand my situation or why I was struggling.*** My living situation was chaotic and I had no safe space"<br>• "***Undiagnosed AuDHD*** pubescent child starting at elite private school on scholarship, ***no accommodations,*** with undiagnosed neurodivergent single mother on low income constantly criticising (& chronic pain)"<br>• "I was sexually abused by my ex boyfriend and wasnt able to access support i was also being bullied by students and teachers at |

| | | | |
|---|---|---|---|
| | | | my school ***my autism and adhd wasnt diagnosed at the time*** i was spiralling”<br>• “I was raped as a child. In addition, I was ***undiagnosed autistic and struggled hugely understanding why I felt so different to everyone else. I felt stupid, clumsy, awkward, couldn’t communicate.***”<br>• “***When younger not understanding my undiagnosed condition and thinking I was mental because of my thoughts and behaviours***. Later due to domestic violence.”<br>• “Symptoms of hopelessness got worse as I got older and I kept going through breakdowns. ***That was before my diagnosis at the age of 51”***<br>• “I was treated for severe depression, developing into anxiety**.** I was on long term sick leave and had been subjected to unfair treatment. My daughter had just been diagnosed ASD. ***I was but unaware of it”***<br>• “***Not knowing why things were wrong.*** Wanting to be/feel normal. Not feeling able to be myself.”<br>• “My workplace was conducting a neurodiversity campaign. I felt like I was being ‘studied’ or ‘examined’ it’s almost like they knew I was ND before I did. ***I only found out last year”***<br>• “I didn't fit in at school and I was in an immense amount of physical pain due to growing too quickly and ***not being given*** |

| | | | |
|---|---|---|---|
| | | | ***the proper diagnosis***/meds by doctors as they just thought it was growing pains”<br>• “My Dad commited suicide when I was 16 and I was raped at 15 and I didn't have adequate help to cope - ***I wasn't diagnosed with autism until I was almost 30 and trying to get help with my mental health”***<br>• “Chronic fatigue, stress, bullying at school and then work, no access to support for mental health and ***waiting years for autism assessment”***<br>• “So much suffering in this world with animals. Also, being lost in a neurotypical world BEING SWALLOWED up. ***No help or understanding either b4 diagnosis late in life!!*** Should b protected. Invisible”<br>• “Having family and romantic relationships which were toxic. Being alone to manage everything. Having no-one to go to for advice or support. So lonely. Such an outsider. Not diagnosed then. My Dr 👍”<br>• “***Not knowing the cause of my difficulties: I was diagnosed just a few years ago,*** after I had become distressed about continually being used and then rejected by people.”<br>• “***Managing ADHD alongside autism (while not knowing I had the condition until about 22).*** The feeling like I'm behind my peers in terms of mental”<br>• “Undiagnosed adhd. Zero self awareness or understanding  (above). Undiagnosed |

| | | | |
|---|---|---|---|
| | | | autism. Niall quinn diabetes clinic experience despite seeing a psychologist aligned with clinic, traits not picked up on."<br>• "NHS GIC waiting list/lack of informed consent system for HRT. being unable to convince my parents to consent to CAMHS referring me to the GIC. ***unexplained autism symptoms derailing my education."***<br>• ***"I have only in the last 12 months come to realise I am autistic*** (I'm high masking/low needs) and ***did not understand*** why I was not normal, nor how to look after myself as an autistic."<br>• "***Not knowing I was autistic*** and therefore not even knowing what is was that needed to be accommodated. Feeling broken and like a failure for not being able to do or be like other around me"<br>• "I knew I was different, you liked me or didn't. I had a lovely husband and my differences really have resurfaced. ***Diagnosed autistic on my 61st Birthday***"<br>• "***Not understanding*** who I was or that I was autistic was probably a huge factor."<br>• "I have tried to kill myself because I ***didn't know why*** I was so different and everything I did upset people or made them hate me."<br>• "Not realising I was autistic and that's why I was struggling to cope, feeling I was stupid and a failure. Formal diagnosis age 56." |

| | | | |
|---|---|---|---|
| | | | • "Thinking I wasn't made right/ too different to others due to not knowing I am autistic"<br>• "I was not diagnosed until I was 59 years old. Many of my emotional problems were related to ***not knowing*** why I am like I am."<br>• "I had not recived an autism diagnoses until I was an adult, I felt like id never fit anywhere, I ***didnt understand*** why I found things difficult."<br>• "***Not having an autism diagnosis*** and ***not knowing*** why I was different and why I couldn't fit in"<br>• "***Not understanding*** why I find life so hard (as I wasn't diagnosed until adulthood)"<br>• "Unable to understand self and having no answers"<br>• "***Partly as a consequence of my autism diagnosis***, the notion this suggested that there was something fundamentally wrong with me and that those people who had been awful to me were right somehow."<br>• ***"I was undiagnosed until my late 50's***- I always knew I was different/had issues but neve***r understood*** what they were"<br>• "Undiagnosed autism, and misdiagnosis and mistreatment as a result."<br>• "Not being diagnosed, just told I was wierd."<br>• "medical personal misunderstandings that related  to physiological injury but was |

| | | | |
|---|---|---|---|
| | | | perceived as mental health problem at that time. also, was accused of stuff that did not partake but was un-dx autist ."<br>• "Being groomed online at a young age (about 14) Undiagnosed neurodiverse and didn't realise what was happening and how I was treated."<br>• "Being mis judged around having undiagnosed autism until it was recognised a year ago. Eg an autistic meltdown has been seen as aggressive, violent, attention seeking"<br>• "Undiagnosed autism"<br>• "Primarily sensory overwhelm. I needed noise cancelling headphones and assistance but ***my parents did not know I was autistic.*** They tried their best but their attempts at helping nearly killed me."<br>• "Being diagnosed as autistic at 61 but family do not relate."<br>• "People in my life who will not accept diagnosis if I did get one, causing me to mask more and be in a spiral of despair"<br>• "Childhood physical and emotional abuse - a lot of what I was 'disciplined' for were ***manifestations of my autistic traits*** and I was denied access to support by my parents (e.g. counselling)."<br>• "Autism had not been diagnosed at this point so my symptoms were not addressed properly." |

| | | | |
|---|---|---|---|
| | | | • "Feeling trapped and being a known figure in the community - minister's wife. ***Undiagnosed autism***. Feeling like the GP couldn't help - being passed from one professional to another." <br>• "I was lost. I wasn't diagnosed until 41 after years of repeated GP visits with low mood and repetitive thoughts. Tried counselling and ant-depressants - ***nothing worked of course - I was autistic"*** <br>• ***"Being undiagnosed*** and so not understanding why I didn't measure up. Setting too high a standard for my. Others doing that. Trying to be what everyone one wanted. Struggling financially despite doing b" <br>• "not understanding or realising that I'm autistic, but ***trying to be as "normal" as possible"*** <br>• "Not knowing what my condition was... Frustration" <br>• "Not understanding why i was feeling what i was feeling. (Very important). Constent stress and overwhelm over many years. (Very important). Getting the wrong help. (Very important)" <br>• "I just always felt so worthless and disgusting and weird. I hated myself as a teen & ***thought I was evil for having meltdowns and getting mad at my loved ones.***" |

**<u>Supplementary item 5: ANOVA of contributing factors to suicidal thoughts and feelings: statistical notations for covariate effects and three-way interaction</u>**

**Effects of covariates**

In our mixed ANOVA, we examined within-subject differences in contributing factors (a within-subjects variable with 19 levels) as a function of between-subjects variables gender and age. To control for their effects, we included as covariates ethnicity (2 levels), autistic status (2 levels), educational attainment (5 levels) and employment (4 levels). Although between-subject effects suggested highest level of educational attainment (F [1, 1307] = 22.13, p < .001), current employment status (F [1, 1307] = 14.95, p < .001) and ethnicity (F [1, 1307] = 5.90, p = .015) exert some degree of effect which was controlled for, examination of these factors individually revealed that ratings of contributing factors did not differ significantly as a function of these covariates. Participant status as formally diagnosed or self-identifying did not significantly affect ratings of contributing factors (F [1, 1307] = 1.49, p = .222), suggesting that formally diagnosed autistic and possibly autistic participants responded similarly to the factors which might contribute to suicidal thoughts and feelings.

**Three-way interaction of contributing factors, age group, and gender**

There was a significant three-way interaction of contributing factors, age group and gender (F [80.83, 17608.42] =1.37, p = .016). To explore this interaction, we examined effects of age group on ratings of contributing factors in the cisgender women and cisgender men groups separately, controlling for the same covariates. Unfortunately, due to the heavily skewed distribution of ages in the transgender, gender-divergent and gender-questioning group (where 184 participants were aged 25 and under, 93 were aged between 26-40, 33 were aged

between 41-55, and only 9 were aged 56 or above), we were unable to examine effects of age in this group.

***Cisgender women (n=693)***

Among cisgender female participants, 173 were aged 25 and under, 233 were aged 26-40, 198 were aged 41-55, and 89 were aged 56 or older. With sphericity violated, Greenhouse-Geisser-adjusted values are reported. Statistics reported herein as significant were confirmed as significant at a false discovery rate (FDR) of $p < .05$.

As per analyses presented in the main paper, we observed a main effect of contributing factors, indicating simply that participants varied in their ratings of these factors ($F$ [13.24, 9069.91] = 21.06, $p < .001$, $\eta^{2part} = .03$), and an interaction of age group by contributing factors ($F$ [39.72, 9069.91] = 7.92, $p < .001$, $\eta^{2part} = .03$). There were, as above, significant main effects of covariates ethnicity ($F$ [1, 685] = 11.36, $p < .001$, $\eta^{2part} = .02$), educational attainment ($F$ [1, 685] = 12.14, $p < .001$, $\eta^{2part} = .02$) and employment status ($F$ [1, 685] = 21.15, $p < .001$, $\eta^{2part} = .03$), but not autism diagnostic status ($F$ [1, 685] =1.98, $p = .160$). These effects indicated that despite minor impacts of ethnicity, educational attainment and employment which were controlled for, effects of age group were still significant.

Disentangling the two-way interaction by looking at contributing factors separately, we found that autistic women of different ages only differed in their ratings for certain factors, as depicted in Part A of Supplementary Figure 1:

**Supplementary Figure 1**

*Main effects of age in A) cisgender autistic women and B) cisgender autistic men*

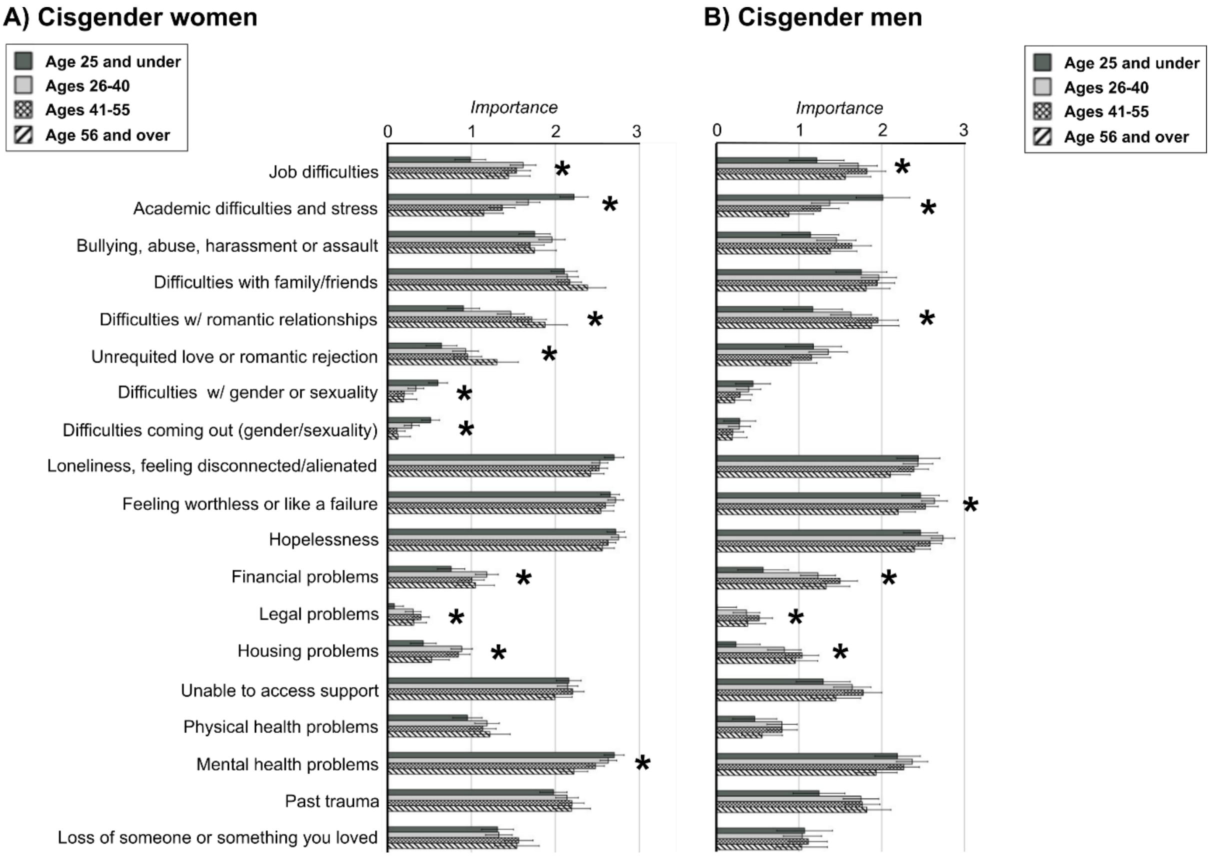


*Note*. Part A depicts main effects of age group on contributing factors to suicidal thoughts and feelings in cisgender autistic women; Part B depicts main effects in cisgender autistic men. Asterisks indicate differences significant at an FDR-corrected p value of p < .05.

As per the main analysis, autistic women of different ages varied in their importance ratings for job difficulties, academic difficulties and stress, difficulties with romantic relationships, difficulties accepting or understanding their gender/sexuality, difficulties coming out to others, financial problems, legal problems, housing problems, and mental health problems. *Unlike* the main analysis, autistic women did *not* differ in their importance ratings for hopelessness, physical health problems, past trauma and loss of someone or something they

loved, suggesting these factors were rated similarly important across women of different ages. Moreover, in contrast to the *absence* of significant age differences for unrequited love in the main analysis with all participants, this factor was differentially important to autistic women.

***Cisgender men (n=311)***

With fewer cisgender male participants, age groups were smaller: 49 participants were aged 25 and under, 101 were aged 26-40, 102 were aged 41-55, and 59 were aged 56 or older. These results should, as such, be interpreted with caution. As before, we report Greenhouse-Geisser-adjusted values and statistics significant at a false discovery rate (FDR[9]) of $p < .05$. We observed just the same effects in cisgender men: a main effect of contributing factors, ($F$ [13.30, 4030.01] = 6.05, $p < .001$, $\eta^{2part} = .02$), and an interaction of age group by contributing factors ($F$ [39.90, 4030.01] = 3.02, $p < .001$, $\eta^{2part} = .03$). Among covariates, only current employment ($F$ [1, 303] = 7.87, $p = .005$, $\eta^{2part} = .03$) had had an effect which had been controlled for. Effects of ethnicity ($F$ [1, 303] = 1.05, $p = .307$, $\eta^{2part} = .003$), educational attainment ($F$ [1, 303] = .55, $p = .459$, $\eta^{2part} = .002$) and autism diagnostic status ($F$ [1, 303] = .22, $p = .642$) were non-significant.

Further exploration of the two-way interaction by examination of individual contributing factors suggested, again, that autistic men of different ages differed in their importance ratings for some but not all factors, as depicted in Part B of Supplementary Figure 1.

Just like autistic women and all participants in the main analysis, autistic men of different ages varied significantly in their importance ratings for job difficulties, academic difficulties and stress, financial problems, legal problems, and housing problems. *Unlike* autistic women, autistic men did *not* differ in their importance ratings for unrequited love, difficulties with romantic relationships, difficulties accepting or understanding their gender/sexuality,

difficulties coming out to others, or mental health. However, unlike women, they differed in their importance ratings for feelings of worthlessness/feeling like a failure, which were greatest in the 26-40 age group.

**Supplementary item 6: Statistical notations for planned comparisons of gender and age effects on contributing factors to suicidal thoughts and feelings**

## Planned comparisons for gender

| Factors in suicidal thoughts and feelings | Main effect | Cisgender men (n=311) (*REF.*) | | Cisgender women (n=693) | | | Transgender / gender-divergent / gender-questioning (n=319) | | |
|---|---|---|---|---|---|---|---|---|---|
| | | ***M*** | ***SD*** | ***M*** | ***SD*** | **Contrast with ref.** **(mean difference)** | ***M*** | ***SD*** | **Contrast with ref.** **(mean difference)** |
| Job difficulties | $F(2, 1343) = 2.59, p = .075, \eta^{2part} = .004$ | 1.65 | 1.17 | 1.42 | 1.22 | -.18, p = .031 | 1.13 | 1.18 | -.21, p = .118 |
| Academic difficulties and stress | $F(2, 1344) = 5.71, p = .003, \eta^{2part} = .01$ | 1.37 | 1.15 | 1.67 | 1.15 | .24, p = .002 | 1.90 | 1.06 | .31, p = .012 |
| Bullying, abuse, harassment and assault | $F(2, 1342) = 16.41, p < .001, \eta^{2part} = .02$ | 1.45 | 1.20 | 1.82 | 1.22 | .43, p < .001 | 1.88 | 1.15 | .63, p < .001 |
| Difficulties with family/friends | $F(2, 1343) = 13.09, p < .001, \eta^{2part} = .02$ | 1.91 | 1.09 | 2.18 | 1.00 | .34, p < .001 | 2.19 | 1.00 | .45, p < .001 |
| Difficulties with romantic relationships | $F(2, 1342) = 4.59, p = .010, \eta^{2part} = .01$ | 1.70 | 1.28 | 1.47 | 1.28 | -.12, p = .158 | 1.03 | 1.18 | -.43, p = .003 |
| Unrequited love or romantic rejection | $F(2, 1340) = 4.58, p = .010, \eta^{2part} = .01$ | 1.17 | 1.91 | .92 | 1.20 | -.17, p = .037 | .63 | 1.00 | -.38, p = .004 |
| Difficulty understanding or accepting gender or sexuality | $F(2, 1339) = 43.44, p < .001, \eta^{2part} = .06$ | .34 | .75 | .35 | .75 | -.01, p = .925 | 1.25 | 1.11 | .82, p < .001 |
| Difficulty coming out to others | $F(2, 1339) = 31.60, p < .001, \eta^{2part} = .05$ | .25 | .70 | .27 | .70 | .01, p = .936 | 1.15 | 1.08 | .67, p < .001 |
| Loneliness or feeling disconnected/alienated | $F(2, 1349) = 5.25, p = .005, \eta^{2part} = .01$ | 2.37 | .91 | 2.55 | .75 | .18, p = .001 | 2.59 | .75 | .11, p = .222 |
| Feeling worthless or like a failure | $F(2, 1346) = 6.11, p = .002, \eta^{2part} = .01$ | 2.50 | .81 | 2.64 | .72 | .18, p < .001 | 2.66 | .68 | .17, p = .084 |
| Hopelessness | $F(2, 1344) = 8.12, p < .001, \eta^{2part} = .01$ | 2.59 | .72 | 2.69 | .67 | .13, p = .005 | 2.79 | .55 | .29, p < .001 |
| Financial problems | $F(2, 1337) = 1.58, p < .207, \eta^{2part} = .002$ | 1.23 | 1.12 | 1.02 | 1.09 | -.18, p = .133 | 1.01 | 1.09 | -.11, p = .135 |
| Legal problems | $F(2, 1337) = .165, p = .848, \eta^{2part} = .0001$ | .37 | .85 | .28 | .73 | -.02, p = .672 | .15 | .52 | -.04, p = .595 |
| Housing problems | $F(2, 1341) = .339, p = .712, \eta^{2part} = .001$ | .82 | 1.09 | .72 | 1.03 | -.05, p = .445 | .71 | 1.03 | -.01, p = .965 |
| Unable to access support | $F(2, 1339) = 37.49, p < .001, \eta^{2part} = .05$ | 1.61 | 1.17 | 2.14 | .98 | .60, p < .001 | 2.22 | .97 | .66, p < .001 |
| Physical health | $F(2, 1343) = 22.40, p < .001, \eta^{2part} = .03$ | .73 | .98 | 1.13 | 1.17 | .43, p < .001 | 1.03 | 1.07 | .51, p < .001 |
| Mental health problems | $F(2, 1346) = 17.35, p < .001, \eta^{2part} = .03$ | 2.24 | .97 | 2.56 | .77 | .33, p < .001 | 2.61 | .72 | .29, p = .001 |
| Past trauma | $F(2, 1341) = 24.44, p < .001, \eta^{2part} = .04$ | 1.70 | 1.12 | 2.13 | 1.05 | .59, p < .001 | 2.07 | 1.09 | .51, p < .001 |
| Losing someone/something you loved | $F(2, 1341) = 13.00, p < .001, \eta^{2part} = .02$ | 1.05 | 1.19 | 1.42 | 1.24 | .43, p < .001 | 1.05 | 1.17 | .21, p = .129 |

*Note*. Table displays main effects of gender for each contributing factor to suicidal thoughts and feelings. Effects that were non-significant at a false discovery rate (FDR)-corrected alpha level of $p < .05$ are shaded in grey.

## Planned comparisons for age

| Factors in suicidal thoughts and feelings | Main effect | 25 and under (n=406) *(REF.)* | | 26 to 40 (n=427) | | | 41 to 55 (n=333) | | | 56 and above (n=157) | | |
|---|---|---|---|---|---|---|---|---|---|---|---|---|
| | | ***M*** | ***SD*** | ***M*** | ***SD*** | **Contrast with ref.** (mean difference) | ***M*** | ***SD*** | **Contrast with ref.** (mean difference) | ***M*** | ***SD*** | **Contrast with ref.** (mean difference) |
| Job difficulties | F(3, 1343) = 18.03, p < .001, $\eta^{2part}$ = .04 | .91 | 1.10 | 1.64 | 1.18 | .63, p < .001 | 1.64 | 1.21 | .65, p < .001 | 1.54 | 1.20 | .47, p = .004 |
| Academic difficulties and stress | F(3, 1344) = 24.31, p < .001, $\eta^{2part}$ = .05 | 2.17 | .96 | 1.60 | 1.10 | -.51, p < .001 | 1.33 | 1.16 | -.78, p < .001 | 1.14 | 1.17 | -.82, p < .001 |
| Bullying, abuse, harassment and assault | F(3, 1342) = 2.13, p = .095, $\eta^{2part}$ = .01 | 1.76 | 1.18 | 1.78 | 1.20 | .14, p =.096 | 1.72 | 1.26 | .28, p = .014 | 1.68 | 1.22 | .21, p = .209 |
| Difficulties with family/friends | F(3, 1343) = 2.42, p = .064, $\eta^{2part}$ = .01 | 2.11 | 1.10 | 2.10 | 1.03 | .09, p = .249 | 2.12 | 1.05 | .17, p = .082 | 2.19 | 1.03 | .354, p = .013 |
| Difficulties with romantic relationships | F(3, 1342) = 20.24, p < .001, $\eta^{2part}$ = .04 | .94 | 1.17 | 1.43 | 1.25 | .44, p < .001 | 1.81 | 1.26 | .87, p < .001 | 1.82 | 1.28 | .67, p < .001 |
| Unrequited love or romantic rejection | F(3, 1340) = 2.89, p = .034, $\eta^{2part}$ = .01 | .68 | 1.05 | .96 | 1.16 | .20, p = .033 | 1.01 | 1.20 | .30, p = .006 | 1.12 | 1.28 | .25, p = .119 |
| Difficulty understanding or accepting gender or sexuality | F(3, 1339) = 8.05, p < .001, $\eta^{2part}$ = .02 | .94 | 1.08 | .53 | .90 | -.20, p = .004 | .29 | .70 | -.39, p < .001 | .27 | .69 | -.24, p = .042 |
| Difficulty coming out to others | F(3, 1339) = 9.30, p < .001, $\eta^{2part}$ = .02 | .83 | 1.07 | .47 | .88 | -.12, p = .070 | .19 | .56 | -.37, p < .001 | .20 | .66 | -.32, p = .004 |
| Loneliness or feeling disconnected/alienated | F(3, 1349) = 3.34, p = .019, $\eta^{2part}$ = .01 | 2.64 | .69 | 2.52 | .77 | -.07, p = .063 | 2.47 | .83 | -.15, p = .074 | 2.29 | .98 | -.32, p = .004 |
| Feeling worthless or like a failure | F(3, 1346) = 2.43, p = .064, $\eta^{2part}$ = .01 | 2.64 | .68 | 2.68 | .67 | .08, p = .059 | 2.58 | .77 | .02, p = .826 | 2.41 | .91 | -.18, p = .080 |
| Hopelessness | F(3, 1344) = 3.94, p = .008, $\eta^{2part}$ = .01 | 2.71 | .63 | 2.77 | .57 | .16, p = .002 | 2.63 | .70 | .03, p = .636 | 2.54 | .81 | .02, p = .847 |
| Financial problems | F(3, 1337) = 20.19, p < .001, $\eta^{2part}$ = .04 | .76 | .97 | 1.21 | 1.11 | .54, p < .001 | 1.21 | 1.15 | .69, p <.001 | 1.18 | 1.12 | .20, p = .185 |
| Legal problems | F(3, 1335) = 15.38, p < .001, $\eta^{2part}$ = .03 | .09 | .38 | .28 | .73 | .26, p < .001 | .42 | .87 | .43, p < .001 | .40 | .90 | .33, p < .001 |
| Housing problems | F(3, 1341) = 21.06, p < .001, $\eta^{2part}$ = .05 | .44 | .82 | .88 | 1.08 | .56, p < .001 | .91 | 1.15 | .63, p < .001 | .79 | 1.07 | .27, p = .056 |
| Unable to access support | F(3, 1339) = 2.45, p = .062, $\eta^{2part}$ = .01 | 2.09 | 1.02 | 2.01 | 1.04 | .13, p = .122 | 2.05 | 1.06 | .24, p = .012 | 1.94 | 1.16 | -.01, p = .928 |
| Physical health | F(3, 1343) = 5.18, p < .001, $\eta^{2part}$ = .01 | .90 | 1.05 | 1.05 | 1.12 | .28, p = .001 | 1.05 | 1.17 | .36, p < .001 | 1.14 | 1.14 | .20, p = .196 |
| Mental health problems | F(3, 1346) = 5.38, p = .001, $\eta^{2part}$ = .01 | 2.63 | .70 | 2.55 | .79 | .05, p = .489 | 2.43 | .87 | -.02, p = .748 | 2.15 | .97 | -.39, p < .001 |
| Past trauma | F(3, 1341) = 6.71, p < .001, $\eta^{2part}$ = .02 | 1.90 | 1.13 | 2.03 | 1.08 | .29, p <.001 | 2.06 | 1.09 | .36, p < .001 | 2.14 | 1.00 | .49, p = .001 |
| Losing someone/something you loved | F(3, 1341) = 4.29, p = .005, $\eta^{2part}$ = .01 | 1.15 | 1.21 | 1.14 | 1.19 | .01, p = .942 | 1.41 | 1.24 | .34, p = .002 | 1.41 | 1.26 | .23, p = .183 |

*Note*. Table displays main effects of age group for each contributing factor to suicidal thoughts and feelings. Effects that were non-significant at a false discovery rate (FDR)-corrected alpha level of $p < .05$ are shaded in grey.

**Supplementary item 7: Full statistical notations for multinomial regression, including replication with covariates**

Full statistical notations for Table 2 from the manuscript are presented here, indicating both significant and non-significant associations between lifetime suicidality and perceived importance of each contributing factor:

| Contributing factors as predictors of lifetime suicidality | | | | | | |
|---|---|---|---|---|---|---|
| Contrast with suicide attempts (reference category) | Variables | B (SE) | Wald $\chi^2$ | *p* | OR of being in the suicide attempts group | 95% CI for OR |
| 1. Passing thoughts | Importance of academic difficulties/stress | .01 (.24) | .002 | .964 | 1.01 | .64, .161 |
| | **Importance of bullying** | **.61 (.25)** | **6.04** | **.014** | **1.84** | **1.13, 2.98** |
| | Importance of friend/family difficulties | .27 (.22) | 1.44 | .230 | 1.31 | .84, 2.04 |
| | Importance of loneliness | -.11 (.23) | .23 | .633 | .90 | .57, 1.41 |
| | Importance of worthlessness/failure | .12 (.25) | .24 | .627 | 1.13 | .69, 1.87 |
| | **Importance of hopelessness** | **1.08 (.26)** | **16.91** | **< .001** | **2.95** | **1.76, 4.94** |
| | Importance of being unable to access support | .51 (.24) | 4.67 | .031 | 1.67 | 1.05, 2.66 |
| | **Importance of mental health problems** | **1.10 (.22)** | **23.92** | **< .001** | **3.00** | **1.93, 4.66** |
| | Importance of past trauma | .27 (.24) | 1.18 | .277 | 1.30 | .81, 2.11 |
| 2. Suicide ideation without plans or attempts | Importance of academic difficulties/stress | -.08 (.17) | .26 | .613 | .92 | .66, 1.27 |
| | **Importance of bullying** | **.56 (.17)** | **10.73** | **.001** | **1.76** | **1.25, 2.47** |
| | Importance of friend/family difficulties | .01 (.16) | .001 | .973 | 1.01 | .73, 1.38 |
| | Importance of loneliness | -.12 (.18) | .44 | .509 | .89 | .63, 1.26 |
| | Importance of worthlessness/failure | -.04 (.20) | .03 | .854 | .96 | .65, 1.42 |
| | Importance of hopelessness | .41 (.22) | 3.67 | .055 | 1.51 | .99, 2.30 |

| | | | | | | |
|---|---|---|---|---|---|---|
| | **Importance of being unable to access support** | **.54 (.16)** | **10.98** | **.001** | **1.72** | **1.25, 2.38** |
| | Importance of mental health problems | .33 (.17) | 3.81 | .051 | 1.40 | 1.00, 1.95 |
| | **Importance of past trauma** | **.52 (.17)** | **9.05** | **.003** | **1.68** | **1.20, 2.35** |
| 3. Suicide plans | Importance of academic difficulties/stress | .03 (.15) | .04 | .851 | 1.03 | .76, 1.38 |
| | Importance of bullying | .02 (.15) | .02 | .896 | 1.02 | .76, 1.37 |
| | Importance of friend/family difficulties | .04 (.15) | .07 | .785 | 1.04 | .78, 1.39 |
| | Importance of loneliness | -.03 (.16) | .04 | .841 | .97 | .70, 1.33 |
| | Importance of worthlessness/failure | -.11 (.19) | .35 | .556 | .90 | .62, 1.29 |
| | Importance of hopelessness | .22 (.21) | 1.17 | .279 | 1.25 | .83, 1.88 |
| | **Importance of being unable to access support** | **.56 (.15)** | **14.32** | **< .001** | **1.76** | **1.31, 2.35** |
| | Importance of mental health problems | .18 (.16) | 1.27 | .260 | 1.20 | .87, 1.64 |
| | **Importance of past trauma** | **.47 (.16)** | **9.25** | **.002** | **1.61** | **1.18, 2.18** |
| **Main effects** | Importance of academic difficulties/stress: $\chi^2(3) = .457$, p = .928<br>**Importance of bullying: $\chi^2(3) = 16.43$, p < .001**<br>Importance of friend/family difficulties: $\chi^2(3) = 1.58$, p = .664<br>Importance of loneliness: $\chi^2(3) = .54$, p = .911<br>Importance of worthlessness/failure: $\chi^2(3) = .93$, p = .818<br>**Importance of hopelessness: $\chi^2(3) = 17.70$, p < .001**<br>**Importance of being unable to access support: $\chi^2(3) = 18.88$, p < .001**<br>**Importance of mental health problems: $\chi^2(3) = 25.04$, p < .001**<br>**Importance of past trauma: $\chi^2(3) = 13.12$, p = .004** | | | | | |

We repeated the multinomial regression presented in the paper with the inclusion of gender, age (continuous) and covariates. As per that analysis, suicide attempts were the reference group; OR and their CIs are presented for likelihood of being in the suicide attempts group. For gender,

cisgender men were the reference group against which cisgender women and transgender, gender-divergent and gender-questioning participants were compared. In relation to covariates: for autistic status, possibly autistic was the reference group; for ethnicity, minority ethnicity was the reference group; for highest educational attainment, postgraduate qualifications were the reference group; for employment, retired/undisclosed were the reference group. As such, negative *B* coefficients for gender (transgender/gender-divergent/gender-questioning), autistic status (formally diagnosed) and education (AS/A-Level) reflect *lower likelihood* of those group, compared with the respective reference groups of these variables, being in the passing thoughts, suicide ideation, or suicide plans group rather than the suicide attempt group.

While increasing the variance explained by the model ($\chi^2(63) = 289.74$, $p < 0.001$; Nagelkerke $R^2 = .21$), the addition of gender, age and covariates in the second model did not change the aforementioned main effects of contributing factors Bullying, Hopelessness, Being unable to access support, Mental health problems and Past trauma.

| Model: Contributing factors (9), gender, age and covariates as predictors of lifetime suicidality | | | | | | |
|---|---|---|---|---|---|---|
| Contrast with suicide attempts (reference category) | Variables | B (SE) | Wald $\chi^2$ | p | OR of being in the suicide attempts group | 95% CI for OR |
| 1. Passing thoughts | Importance of academic difficulties/stress | .04 (.25) | .02 | .883 | 1.04 | .64, 1.68 |
| | **Importance of bullying** | **.58 (.25)** | **5.32** | **.021** | **1.79** | **1.09, 2.93** |
| | Importance of friend/family difficulties | .28 (.23) | 1.46 | .227 | 1.32 | .84, 2.08 |
| | Importance of loneliness | -.05 (.24) | .05 | .816 | .95 | .60, 1.50 |
| | Importance of worthlessness/failure | .17 (.26) | .42 | .517 | 1.18 | .71, 1.97 |
| | **Importance of hopelessness** | **.97 (.27)** | **13.09** | **< .001** | **2.64** | **1.56, 4.48** |

| | | | | | | |
|---|---|---|---|---|---|---|
| | Importance of being unable to access support | .44 (.25) | 3.17 | .075 | 1.55 | .96, 2.51 |
| | **Importance of mental health problems** | **1.08 (.23)** | **21.22** | **< .001** | **2.93** | **1.86, 4.64** |
| | Importance of past trauma | .34 (.25) | 1.85 | .174 | 1.41 | .86, 2.30 |
| | **Gender (transgender, gender-divergent or gender-questioning vs. cisgender men [r])** | **-1.18 (.37)** | **10.45** | **.001** | **.31** | **.15, .63** |
| | Gender (cisgender women vs. cisgender men [r]) | .05 (.26) | .04 | .841 | 1.05 | .64, 1.74 |
| | Age | -.01 (.01) | 1.23 | .268 | .99 | .97, 1.01 |
| | Ethnicity (white vs minority ethnicity [r]) | -.33 (.33) | .99 | .319 | .72 | .37, 1.38 |
| | **Autistic status (formally diagnosed vs. possibly autistic [r])** | **.57 (.21)** | **6.99** | **.008** | **1.76** | **1.16, 2.68** |
| | Educational attainment (rather not say vs. postgraduate [r]) | .60 (.77) | .61 | .435 | 1.82 | .40, 8.27 |
| | Educational attainment (GCSE-level vs. postgraduate [r]) | -.16 (.34) | .23 | .631 | .85 | .44, 1.64 |
| | Educational attainment (A/AS-Level vs. postgraduate [r]) | -.70 (.40) | 3.13 | .077 | .50 | .23, 1.08 |
| | Educational attainment (diploma/degree vs. postgraduate [r]) | .28 (.27) | 1.05 | .306 | 1.32 | .77, 2.26 |
| | Current employment (employed/student vs. retired/undisclosed [r]) | -.04 (.50) | .01 | .941 | .96 | .36, 2.56 |
| | Current employment (carer/voluntary vs. retired/unknown [r]) | .20 (.59) | .11 | .739 | 1.22 | .38, 3.88 |
| | Current employment (unemployed vs. retired/undisclosed [r]) | -.28 (.53) | .28 | .597 | .76 | .27, 2.18 |
| 2. Suicide ideation without plans or attempts | Importance of academic difficulties/stress | -.10 (.17) | .37 | .543 | .90 | .64, 1.26, |
| | **Importance of bullying** | **.57 (.18)** | **10.43** | **.001** | **1.76** | **1.25, 2.48** |
| | Importance of friend/family difficulties | .02 (.16) | .02 | .900 | 1.02 | .74, 1.41 |
| | Importance of loneliness | -.08 (.18) | .20 | .654 | .92 | .65, 1.31 |
| | Importance of worthlessness/failure | -.02 (.20) | .01 | .925 | .98 | .66, 1.46 |
| | Importance of hopelessness | .35 (.22) | 2.51 | .113 | 1.41 | .92, 2.17 |
| | **Importance of being unable to access support** | **.43 (.17)** | **6.44** | **.011** | **1.54** | **1.10, 2.14** |
| | Importance of mental health problems | .32 (.18) | 3.29 | .070 | 1.38 | .97, 1.95 |
| | **Importance of past trauma** | **.53 (.18)** | **8.94** | **.003** | **1.70** | **1.20, 2.40** |
| | **Gender (transgender, gender-divergent or gender-questioning vs. cisgender men [r])** | **-.83 (.24)** | **11.70** | **.001** | **.44** | **.27, .70** |

| | | | | | | |
|---|---|---|---|---|---|---|
| | Gender (cisgender women vs. cisgender men [r]) | -.27 (.20) | 1.89 | .169 | .76 | .52, 1.12 |
| | Age | -.003 (.01) | .38 | .536 | 1.00 | .98, 1.01 |
| | Ethnicity (white vs minority ethnicity [r]) | -.19 (.25) | .58 | .446 | .83 | .51, 1.34 |
| | **Autistic status (formally diagnosed vs. possibly autistic [r])** | **.47 (.16)** | **8.73** | **.003** | **1.60** | **1.17, 2.19** |
| | Educational attainment (rather not say vs. postgraduate [r]) | .46 (.55) | .70 | .403 | 1.59 | .54, 4.67 |
| | Educational attainment (GCSE-level vs. postgraduate [r]) | -.06 (.23) | .07 | .786 | .94 | .59, 1.48 |
| | Educational attainment (A/AS-Level vs. postgraduate [r]) | -.60 (.27) | 4.98 | .026 | .55 | .33, .93 |
| | Educational attainment (diploma/degree vs. postgraduate [r]) | -.20 (.21) | .93 | .335 | .82 | .55, 1.23 |
| | Current employment (employed/student vs. retired/undisclosed [r]) | .52 (.41) | 1.60 | .205 | 1.68 | .75, 3.77 |
| | Current employment (carer/voluntary vs. retired/unknown [r]) | .05 (.50) | .01 | .922 | 1.05 | .39, 2.82 |
| | Current employment (unemployed vs. retired/undisclosed [r]) | .11 (.43) | .07 | .797 | 1.12 | .48, 2.58 |
| 3. Suicide plans | Importance of academic difficulties/stress | -.07 (.16) | .18 | .676 | .94 | .69, 1.28 |
| | Importance of bullying | -.01 (.16) | .00 | .974 | .99 | .73, 1.35 |
| | Importance of friend/family difficulties | .09 (.15) | .39 | .532 | 1.10 | .82, 1.48 |
| | Importance of loneliness | -.01 (.17) | .01 | .930 | .99 | .71, 1.37 |
| | Importance of worthlessness/failure | -.12 (.19) | .40 | .529 | .89 | .61, 1.29 |
| | Importance of hopelessness | .17 (.21) | .63 | .427 | 1.18 | .78, 1.79 |
| | **Importance of being unable to access support** | **.47 (.16)** | **9.06** | **.003** | **1.60** | **1.18, 2.16** |
| | Importance of mental health problems | .06 (.17) | .11 | .736 | 1.06 | .76, 1.47 |
| | **Importance of past trauma** | **.52 (.16)** | **10.18** | **.001** | **1.68** | **1.22, 2.30** |
| | **Gender (transgender, gender-divergent or gender-questioning vs. cisgender men [r])** | **-.64 (.22)** | **8.46** | **.004** | **.53** | **.34, .81** |
| | Gender (cisgender women vs. cisgender men [r]) | -.39 (.18) | 4.54 | .033 | .68 | .47, .97 |
| | Age | .01 (.01) | 1.05 | .305 | 1.01 | .99, 1.02 |
| | Ethnicity (white vs minority ethnicity [r]) | -.28 (.22) | 1.58 | .209 | .76 | .49, 1.17 |
| | **Autistic status (formally diagnosed vs. possibly autistic [r])** | **.56 (.15)** | **14.22** | **< .001** | **1.74** | **1.31, 2.33** |
| | Educational attainment (rather not say vs. postgraduate [r]) | .30 (.50) | .37 | .541 | 1.35 | .51, 3.58 |

| | | | | | | |
|---|---|---|---|---|---|---|
| | Educational attainment (GCSE-level vs. postgraduate [r]) | -.14 (.22) | .42 | .515 | .87 | .57, 1.33 |
| | Educational attainment (A/AS-Level vs. postgraduate [r]) | -.55 (.24) | 5.20 | .023 | .57 | .36, .92 |
| | Educational attainment (diploma/degree vs. postgraduate [r]) | -.20 (.19) | 1.13 | .287 | .82 | .56, 1.18 |
| | Current employment (employed/student vs. retired/undisclosed [r]) | -.12 (.32) | .13 | .721 | .89 | .47, 1.68 |
| | Current employment (carer/voluntary vs. retired/unknown [r]) | -.88 (.44) | 4.04 | .044 | .41 | .17, .98 |
| | Current employment (unemployed vs. retired/undisclosed [r]) | -.68 (.34) | 4.00 | .046 | .50 | .26, .99 |
| Main effects | Importance of academic difficulties/stress: $\chi^2(3) = .56, p = .906$<br>**Importance of bullying: $\chi^2(3) = 16.19, p = .001$**<br>Importance of friend/family difficulties: $\chi^2(3) = 1.69, p = .639$<br>Importance of loneliness: $\chi^2(3) = .23, p = .972$<br>Importance of worthlessness/failure: $\chi^2(3) = 1.32, p = .725$<br>**Importance of hopelessness: $\chi^2(3) = 13.85, p = .003$**<br>**Importance of being unable to access support: $\chi^2(3) = 11.57, p = .009$**<br>**Importance of mental health problems: $\chi^2(3) = 24.24, p < .001$**<br>**Importance of past trauma: $\chi^2(3) = 13.56, p = .004$**<br>**Gender: $\chi^2(6) = 28.31, p < .001$**<br>Age: $\chi^2(3) = 4.27, p = .234$ | *Covariates*<br>Ethnicity: $\chi^2(3) = 1.95, p = .583$<br>**Autistic status: $\chi^2(3) = 17.54, p < .001$**<br>Educational attainment: $\chi^2(12) = 18.27, p = .108$<br>**Current employment: $\chi^2(9) = 21.00, p = .013$** | | | | |

**Supplementary item 8: Confirmatory logistic regression differentiating between participants with and without lifetime suicide attempts**

To corroborate multinomial regressions with greater power and more clearly delineate participants with and without lifetime suicide attempts, we performed a binary regression categorising participants by the absence or presence of lifetime suicide attempts (reference category). Here, we entered contributing factors in the first block of the model, gender and age in the second block, and covariates in the third.

| Block | Variables | B (SE) | Wald $\chi^2$ | p | OR of being in the suicide attempts group | CI for OR |
|---|---|---|---|---|---|---|
| Block 1: Contributing factors only | Importance of academic difficulties/stress | -.01 (.13) | .01 | .912 | .99 | .77, 1.27 |
| | Importance of bullying | .29 (.13) | 4.87 | .027 | 1.33 | 1.03, 1.72 |
| | Importance of friend/family difficulties | .06 (.13) | .200 | .655 | 1.06 | .83, 1.36 |
| | Importance of loneliness | -.08 (.14) | .290 | .590 | .93 | .70, 1.22 |
| | Importance of worthlessness/failure | -.04 (.16) | .07 | .790 | 1.58 | .70, 1.31 |
| | **Importance of hopelessness** | **.46 (.18)** | **6.65** | **.010** | **1.58** | **1.12, 2.23** |
| | **Importance of being unable to access support** | **.55 (.13)** | **18.73** | **< .001** | **1.73** | **1.35, 2.22** |
| | **Importance of mental health problems** | **.38 (.14)** | **7.68** | **.006** | **1.46** | **1.12, 1.92** |
| | **Importance of past trauma** | **.46 (.13)** | **12.22** | **< .001** | **1.59** | **1.23, 2.06** |
| Block 2: Contributing factors, gender and age | Importance of academic difficulties/stress | -.04 (.13) | .10 | .748 | .96 | .74, 1.24 |
| | Importance of bullying | .29 (.13) | 4.83 | .028 | 1.33 | 1.03, 1.7 |
| | Importance of friend/family difficulties | .05 (.13) | .13 | .724 | 1.05 | .82, 1.34 |
| | Importance of loneliness | -.09 (.14) | .40 | .527 | .91 | .69, 1.21 |
| | Importance of worthlessness/failure | -.02 (.16) | .02 | .881 | .98 | .71, 1.34 |
| | **Importance of hopelessness** | **.43 (.18)** | **5.72** | **.017** | **1.53** | **1.08, 2.17** |
| | **Importance of being unable to access support** | **.52 (.13)** | **16.06** | **.000** | **1.67** | **1.30, 2.15** |

| | | | | | | |
|---|---|---|---|---|---|---|
| | **Importance of mental health problems** | **.36 (.14)** | **6.44** | **.011** | **1.43** | **1.08, 1.88** |
| | **Importance of past trauma** | **.45 (.13)** | **11.30** | **.001** | **1.57** | **1.21, 2.05** |
| | **Gender (transgender, gender-divergent or gender-questioning** | **.67 (.19)** | **12.64** | **< .001** | **1.95** | **1.35, 2.81** |
| | **vs. cisgender men [r])** | .22 (.16) | 1.96 | .162 | 1.24 | .92, 1.69 |
| | Gender (cisgender women vs. cisgender men [r]) | .0002 (.001) | .002 | .964 | 1.00 | .99, 1.01 |
| | Age | | | | | |
| Block 3: Contributing factors, gender, age and covariates | Importance of academic difficulties/stress | -.07 (.14) | .26 | .609 | .93 | .72, 1.22 |
| | Importance of bullying | .28 (.13) | 4.29 | .038 | 1.32 | 1.01, 1.72 |
| | Importance of friend/family difficulties | .09 (.13) | .45 | .504 | 1.09 | .85, 1.41 |
| | Importance of loneliness | -.05 (.14) | .12 | .729 | .95 | .72, 1.26 |
| | Importance of worthlessness/failure | -.03 (.17) | .03 | .852 | .97 | .70, 1.34 |
| | Importance of hopelessness | .40 (.18) | 4.74 | .029 | 1.49 | 1.04, 2.12 |
| | **Importance of being unable to access support** | **.45 (.13)** | **11.31** | **.001** | **1.56** | **1.20, 2.02** |
| | Importance of mental health problems | .32 (.14) | 4.91 | .027 | 1.37 | 1.04, 1.82 |
| | **Importance of past trauma** | **.50 (.14)** | **12.90** | **< .001** | **1.64** | **1.25, 2.15** |
| | **Gender (transgender, gender-divergent or gender-questioning vs. cisgender men [r])** | **.77 (.19)** | **16.07** | **< .001** | **2.16** | **1.48, 3.14** |
| | Gender (cisgender women vs. cisgender men [r]) | .28 (.16) | 3.14 | .076 | 1.33 | .97, 1.82 |
| | Age | .00 (.01) | .001 | .979 | 1.00 | .99, 1.01 |
| | Ethnicity (white vs minority [r]) | .25 (.19) | 1.62 | .203 | 1.28 | .87, 1.88 |
| | **Autistic status (undiagnosed vs. formally diagnosed [r])** | **-.52 (.13)** | **16.56** | **< .001** | **.59** | **.46, .76** |
| | Educational attainment (rather not say vs. postgraduate [r]) | -.40 (.44) | .82 | .367 | .67 | .29, 1.59 |
| | Educational attainment (GCSE-level vs. postgraduate [r]) | .11 (.19) | .36 | .548 | 1.12 | .77, 1.62 |
| | **Educational attainment (A/AS-Level vs. postgraduate [r])** | **.58 (.21)** | **7.66** | **.006** | **1.79** | **1.18, 2.70** |
| | Educational attainment (diploma/degree vs. postgraduate [r]) | .13 (.17) | .59 | .442 | 1.14 | .82, 1.57 |
| | Current employment (employed/student vs. retired/unknown [r]) | -.07 (.30) | .06 | .805 | .93 | .52, 1.66 |

| | | | | | | |
|---|---|---|---|---|---|---|
| | Current employment (carer/voluntary vs. retired/unknown [r]) | .39 (.37) | 1.11 | .292 | 1.47 | .72, 3.04 |
| | Current employment (unemployed vs. retired/unknown [r]) | .39 (.31) | 1.62 | .204 | 1.48 | .81, 2.71 |
| Block 1 Model: | $\chi^2(9) = 120.34$, $p < .001$; Nagelkerke $R^2 = .11$); 65.6% of cases categorised correctly. | | | | | |
| Block 2 Model: | $\chi^2(12) = 136.74$, $p < .001$; Nagelkerke $R^2 = .13$); 65.7% of cases categorised correctly. | | | | | |
| Block 3 Model: | $\chi^2(21) = 182.96$, $p < .001$; Nagelkerke $R^2 = .17$); 67.6% of cases categorised correctly. | | | | | |

For illustrative purposes, the supplementary figure below depicts average importance ratings of contributing factors as a function of a) lifetime STB (as per Supplementary item 7), and b) whether or not participants had ever attempted suicide.

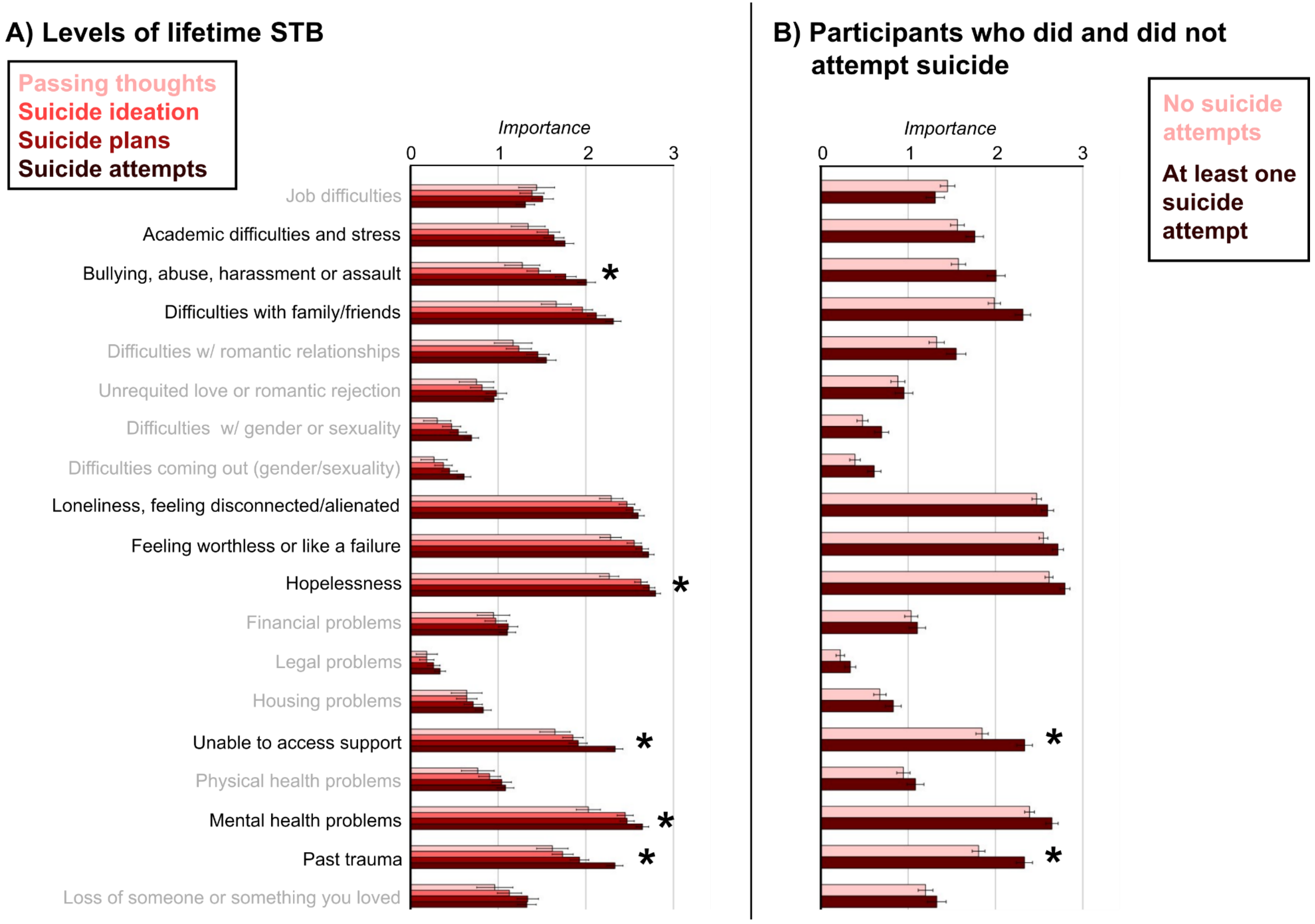


*Note*. This figure is not illustrative of inferential analyses, but depicts, for participants with different degrees of STB, average ratings of importance for all contributing factors. In Part A, these are plotted in relation to different degrees of STB; in Part B, these are plotted in relation to whether or not participants had ever attempted suicide. Contributing factors greyed out were not included as binary predictors in regression analysis, due to being rated as less important on average. Asterisks reflect main effects, still significant once gender, age and covariates were modelled, which differentiated between participants in the multinomial regression (A) and logistic regression (B).

*Note*. This figure is not illustrative of inferential analyses, but depicts, for participants with different degrees of STB, average ratings of importance for all contributing factors. In Part A, these are plotted in relation to different degrees of STB; in Part B, these are plotted in relation to whether or not participants had ever attempted suicide. Contributing factors greyed out were not included as binary predictors in regression analysis, due to being rated as less important on average. Asterisks reflect main effects, still significant once gender, age and covariates were modelled, which differentiated between participants in the multinomial regression (A) and logistic regression (B).

**References**


1. Cassidy S, Bradley L, Shaw R, Baron-Cohen S. Risk markers for suicidality in autistic adults. *Mol Autism.* 2018;9(1):42-42.
2. Jachyra P, Lai M-C, Zaheer J, et al. Suicidal thoughts and behaviours among autistic adults presenting to the psychiatric emergency department: an exploratory chart review. *J Autism Dev Disord.* 2021:1-9.
3. Goodall C. 'I felt closed in and like I couldn't breathe': A qualitative study exploring the mainstream educational experiences of autistic young people. *Autism & Developmental Language Impairments.* 2018;3:2396941518804407.
4. Ng-Cordell E, Rai A, Peracha H, et al. A qualitative study of self and caregiver perspectives on how autistic individuals cope with trauma. *Frontiers in Psychiatry.* 2022;13:825008.
5. Pelton M, Crawford H, Bul K, et al. The role of anxiety and depression in suicidal thoughts for autistic and non-autistic people: a theory-driven network analysis. *Suicide and Life-Threatening Behavior.* 2023;53(3):426-442.
6. Pelton MK, Crawford H, Robertson AE, Rodgers J, Baron-Cohen S, Cassidy S. Understanding Suicide Risk in Autistic Adults: Comparing the Interpersonal Theory of Suicide in Autistic and Non-autistic Samples. *J Autism Dev Disord.* 2020:1-18.
7. Moseley R, Gregory NJ, Smith P, Allison C, Cassidy S, Baron-Cohen S. The relevance of the interpersonal theory of suicide for predicting past-year and lifetime suicidality in autistic adults. *Mol Autism.* 2022;13(1):1-17.
8. Hedley D, Uljarevic M, Wilmot M, Richdale A, Dissanayake C. Understanding depression and thoughts of self-harm in autism: a potential mechanism involving loneliness. *Res Autism Spectr Disord.* 2018;46:1-7.

9. Benjamini Y, Yekutieli D. The control of the false discovery rate in multiple testing under dependency. *Annals of Statistics.* 2001:1165-1188.
